# Stereoselective Synthesis of 2-Deoxythiosugars from Glycals

**DOI:** 10.3390/molecules27227979

**Published:** 2022-11-17

**Authors:** Xueying You, Yifei Cai, Chenyu Xiao, Lijuan Ma, Yong Wei, Tianpeng Xie, Lei Chen, Hui Yao

**Affiliations:** 1 Hubei Key Laboratory of Natural Products Research and Development, Key Laboratory of Functional Yeast (China National Light Industry), College of Biological and Pharmaceutical Sciences, China Three Gorges University, Yichang 443002, China; 2 Yichang Humanwell Pharmaceutical Co., Ltd., Yichang 443000, China

**Keywords:** 2-deoxythioglycosides, glycosylation, silver triflate, stereoselective synthesis, glycals

## Abstract

2-deoxythiosugars are more stable than 2-deoxysugars occurring broadly in bioactive natural products and pharmaceutical agents. An effective and direct methodology to stereoselectively synthesize α-2-deoxythioglycosides catalyzed by AgOTf has been developed. Various alkyl thiols and thiophenols were explored and the desired products were formed in good yields with excellent α-selectivity. This method was further applied to the syntheses of *S*-linked disaccharides and late-stage 2-deoxyglycosylation of estrogen, L-menthol, and zingerone thiols successfully.

## 1. Introduction

**Scheme 1:**
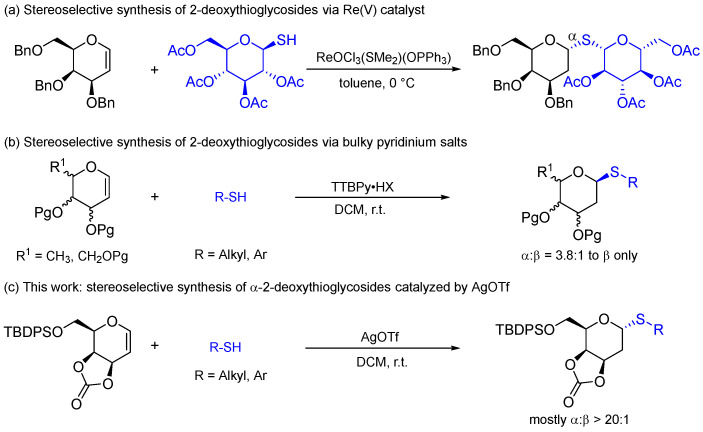
Synthesis of 2-deoxythioglycosides from glycal donors.

2-deoxysugars occur broadly in bioactive natural products and pharmaceutical agents [1,2,3]. They have been applied as clinical drugs in treating various diseases, including heart failure, cancers, and bacterial and viral infections [4,5,6,7]. However, the glycosidic bond of 2-deoxysugar is easily hydrolyzed by enzymes or acids, resulting in a short half-life in vivo, which limits its application in drug development [8,9]. As sulfur is in the same group as oxygen, it is often used as a bioisostere to replace oxygen atoms in medicinal chemistry, indicating a longer half-life and better biological activity [10,11,12,13]. Stereoselective synthesis of 2-deoxythiosugar is quite challenging because there is no C2-group to direct the anomeric selectivity through the neighboring participation effect. Many efforts have been made to develop the strategies of 2-deoxythioglycosylation. Conventionally, 2-deoxysugar synthesis studies could be performed with saturated glycosyl donors, the high stereoselectivity of which relied on a specific structure of a well-assembled glycosyl donor [14,15]. On the other hand, unsaturated glycosyl donors (glycals) could be applied directly to construct 2-deoxysugars stereoselectively [16,17]. Toste and coworkers developed an effective synthesis of 2-deoxyglycosides from glycal donors mediated by a catalytic Re(V)-oxo complex [18]. Recently, Wan′s group successfully achieved access to α-1,1′-2-deoxy thioglycosides stereoselectively catalyzed by ReOCl_3_(SMe_2_)(OPPh_3_) as shown in Scheme 1a [19]. With the development of organocatalysis, Kancharla’s group utilized a bulky pyridinium salt 2,4,6-tri-*tert*-butylpyridine-hydrochloric acid (TTBPy·HCl) to catalyze the reaction between glycals and thiols giving 2-deoxy-β-galactosides with an α/β ratio from 3.8:1 to β only (Scheme 1b) [20]. However, rhenium catalysts are quite expensive and the synthesis of organocatalysts is tedious. Therefore, the stereoselective synthesis of 2-deoxythioglycosides is still highly challenging. In view of our long-standing interest in exploring 3,4-*O*-carbonate-glycal donors [21,22,23,24,25,26,27], herein, we report an effective and stereoselective 2-deoxythioglycosylation catalyzed by the commercially available catalyst silver trifluoromethanesulfonate (AgOTf) as shown in Scheme 1c. The target α-2-deoxythiosugars were able to be obtained in good yields with high stereoselectivity under mild conditions.

## 2. Results

First, 3,4-*O*-carbonate galactal **1a** was adopted as a glycosyl donor and *p*-methylthiophenol **2a** was adopted as a glycosyl acceptor to optimize the conditions for 2-deoxythioglycosylation (Table 1). Initially, Cu(OTf)_2_ was examined as the catalyst, giving 2-deoxysugar **3a** in a yield of 32% with high α-selectivity (α/β > 20:1) in dichloromethane (entry 1). Then, various Lewis acids were screened (entries 2–8). For example, Hg(OTf)_2_ could increase the yield to 59% (entry 2), while Yb(OTf)_3_ decreased the yield to 30% (entry 3). Fe(OTf)_3_ was also able to catalyze this reaction to give the desired product in a 64% yield (entry 4). There was no reaction when Ni(OTf)_2_ and Dy(OTf)_3_ were examined (entries 5, 7). AgOTf could increase the yield to 80% (entry 8), which was the best catalyst for this reaction. Then various solvents were examined, including toluene, THF, MeCN, dimethyl carbonate (DMC), and diethyl carbonate (DEC) (entries 9–15). The yield was lower (43%) in toluene (entry 9), while no reaction was observed in THF (entry 10), acetonitrile (entry 11), DMF (entry 14), and DMSO (entry 15). The green solvent (DMC, entry 12) was applied, and the yield decreased to 57%, while DEC (entry 13) was also attempted to proceed with the reaction, but only a 33% yield was observed. Therefore, the condition was finalized as Ag(OTf) as the catalyst in DCM at room temperature.

**Scheme 2:**
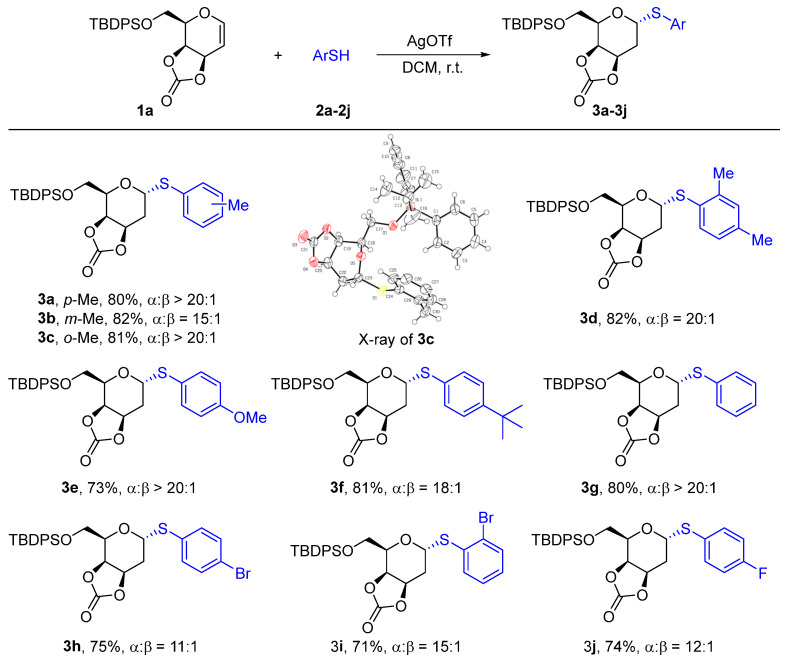
Substrate scope of aryl 2-deoxythioglycosides.

With the optimized condition in hand, the substrate scope was first explored using thiophenols (Scheme 2). The aryl 2-deoxythioglycosides (**3a**-**3c**) were obtained using *p*-, *m*- and *o*-thiocresols as acceptors in yields of 80–82% and with an α/β ratio from 15:1 to > 20:1 determined by ^1^H NMR. Fortunately, the crystal of **3c** was obtained and the configuration was confirmed as α-2-deoxy-D-galactoside by single-crystal X-ray diffraction. 3,4-Dimethylthiophenol also worked well giving **3d** in an 82% yield and with a ratio of α/β = 20:1. Besides methyl substitution thiophenols, 4-methoxythiophenol reacted with 3,4-*O*-carbonate-D-galactal **1a** to form **3e** in a 73% yield with a ratio of α/β > 20:1. The bulkier substitute group on the phenyl ring also tolerated this method well. For example, 4-*tert*-butylthiophenol was applied to generate **3f** in an 81% yield and with an α/β ratio of 18:1. Thiophenol 2-deoxysugar **3g** was obtained stereoselectively (α/β > 20:1) in an 80% yield. When thiophenols were substituted by electron-withdrawing groups, the desired products (**3h**-**3j**) could still be formed in good yields but stereoselectivity decreased slightly. The 2-deoxythiosugar **3h** was formed in a yield of 75% with a ratio of α:β = 11:1 using 4-bromothiophenol as the acceptor. 2-Bromothiophenol and 4-fluorothiophenol were examined and gave target products in a 71–74% yield at the ratios of α:β = 15:1 and α:β = 12:1, respectively.

**Scheme 3:**
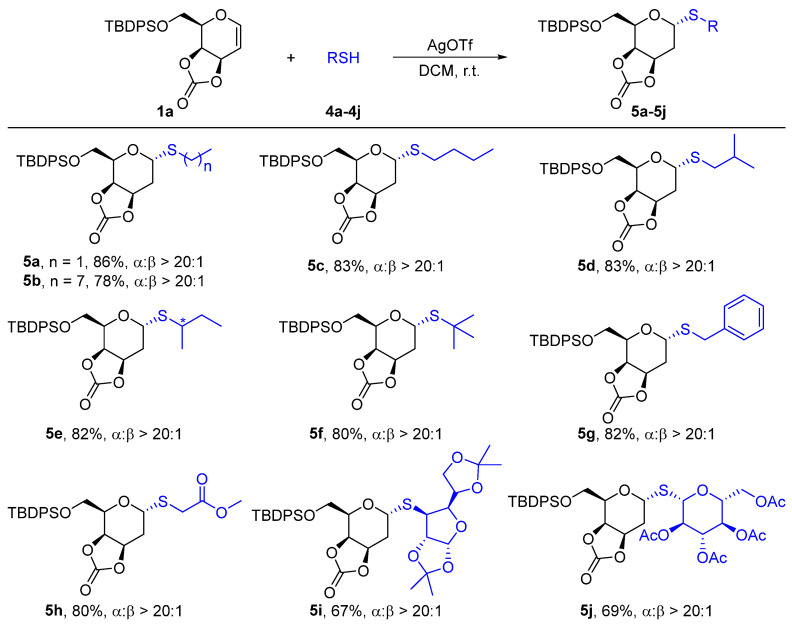
Substrate scope of alkyl 2-deoxythioglycosides.

Besides the substrate study of the thiophenol acceptors, the alkyl thiols were also employed to synthesize 2-deoxythioglycosides, including *S*-linked disaccharides. As shown in Scheme 3, two primary thiols ethyl and *n*-octyl mercaptan were utilized to react with glycal **1a** to form 2-deoxythiosugars **5a** and **5b** in high yields (78–86%) with excellent stereoselectivity (α/β > 20:1). When *n*-butyl, *iso*-butyl, and *sec*-butyl mercaptan were employed, the corresponding α-2-deoxythioglycosides **5c**-**5e** were all able to be generated in high yields (>80%). It is worth noting that the bulky *tert*-butyl mercaptan could also be well compatible with this method and gave **5f** in an 80% yield with exclusive α-selectivity, indicating that the strategy was less affected by steric hindrance. Benzyl mercaptan was applied to form the 2-deoxythiogalactoside **5g** in a yield of 82% with a ratio of α/β > 20:1. Methyl thioglycolate was also applied as a glycosyl acceptor and the desired product **5h** was obtained in an 80% yield. Encouraged by these observations, we continued to study the synthesis of *S*-linked α-disaccharides. For example, **5i** was achieved successfully from 3,4-*O*-carbonate-galactal **1** and 1,2:5,6-di-*O*-isopropylidene-α-D-allofuranose in a yield of 67% with exclusive α-selectivity. The α,β-1,1′-2-deoxythioglycosides **5j** was obtained from glycal **1** and 1-thio-β-D-glucose tetraacetate in a yield of 69% with excellent stereoselectivity (α/β > 20:1).

**Scheme 4:**
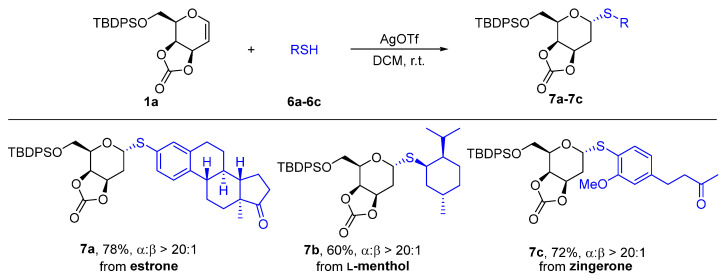
Late-stage 2-deoxythioglycosylation of natural product thiols.

These successes stimulated us to further apply this 2-deoxythioglycosylation methodology to the modification of bioactive natural products (In the Supplementary Materials). Estrogen is a female sex hormone, one of the three main endogenous estrogens, which plays a vital role in human life and is also used as a medicine in clinical treatment [28,29,30]. S-Linked estrone 2-deoxygalactoside **7a** was successfully achieved by AgOTf at room temperature with a 78% yield and excellent α-selectivity (Scheme 4). L-Menthol is the main component of peppermint and also demonstrates analgesic, antibacterial, and anti-inflammatory effects [31]. L-Menthol-2-deoxythioglycoside **7b** was generated in a 60% yield with high α-selectivity. Zingerone extracted from ginger indicates antioxidant, anti-inflammatory and anti-cancer, and antibacterial bioactivity [32,33,34], and it was also converted to be a thiol to react with glycal **1a** giving 2-deoxythiosugar **7c** in a 72% yield with a ratio of α/β > 20:1.

**Scheme 5:**
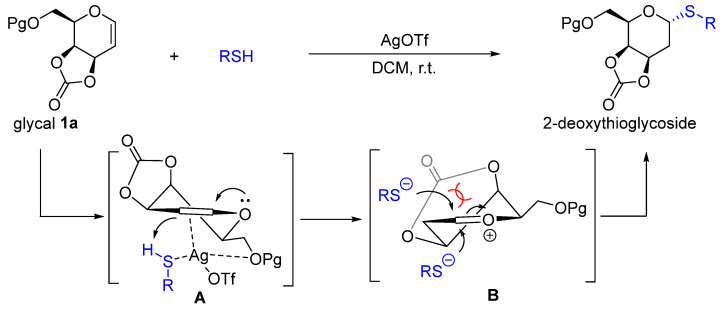
Proposed mechanism.

Based on the results and literature research, a possible mechanism of AgOTf-catalyzed 2-deoxythioglycosylation was proposed as shown in Scheme 5. Silver triflate coordinated with the double bond of glycal from the bottom face to form intermediate **A** because of the steric effect [35,36]. After the pronation of the reactive olefin, the oxocarbenium ion **B** would be generated [1,4,37]. Alkylthio anion (RS^-^) would attack the anomeric position from the bottom face to yield α-2-deoxythioglycosides because the 3,4-*O*-carbonate ring would block the upper face.

## 3. Conclusions

In conclusion, we have developed an effective strategy to synthesize 2-deoxythioglycosides using 3,4-*O*-carbonate glycal donors catalyzed by silver triflate in mild conditions. This reaction could tolerate various alkyl thiols and thiophenols, all the target products were obtained in moderate to good yields with excellent α-selectivity. *S*-Linked disaccharides and late-stage functionalization of natural product thiols were achieved successfully as well. The results of this study suggest that this method may be a promising alternative way to access the 2-deoxythioglycosides applied in natural product synthesis and drug development.

## 4. Materials and Methods

General Procedure. The 3,4-*O*-carbonate glycal donor (0.100 mmol) and thiol reagent (0.110 mmol) were added to anhydrous dichloromethane (2.00 mL) in a Schlenk tube, followed by adding silver triflate (0.01 mmol) under N_2_ atmosphere. The reaction mixture was stirred at room temperature and monitored by TLC. Then, aqueous sodium bicarbonate was added to quench the reaction, extracted with dichloromethane, washed by aqueous sodium bicarbonate and dried by sodium sulfate. The organic layer was collected and removed under reduced pressure to afford a crude product which was purified by silica gel flash chromatography with a gradient solvent system (petroleum ether/ethyl acetate as eluent) to yield 2-deoxythioglycosides.

*4-Methylphenyl-1-thio-6-O*-*(tert-butyldiphenylsilyl)-3,4-O-carbonate-2-deoxy-α-D-galactopyranoside***(3a).** The title compound was prepared according to the general procedure of 2-deoxythioglycosides synthesis and purified by flash column chromatography giving colorless oil 42.7 mg, yield 80%; ^1^H NMR (400 MHz, CDCl_3_) *δ* 7.73–7.63 (m, 4H, Ar-H), 7.47–7.39 (m, 6H, Ar-H), 7.33–7.27 (m, 2H, Ar-H), 7.01–7.03 (m, 2H, Ar-H), 5.45 (dd, *J* = 9.8, 6.5 Hz, 1H, H-1), 5.05 (ddd, *J* = 8.8, 4.4, 2.0 Hz, 1H, H-3), 4.90 (dd, *J* = 8.6, 1.6 Hz, 1H, H-4), 4.16 (ddd, *J* = 7.7, 6.0, 1.6 Hz, 1H, H-5), 3.84 (dd, *J* = 10.2, 7.6 Hz, 1H, H-6), 3.77 (dd, *J* = 10.2, 6.2 Hz, 1H, H-6’), 2.64 (ddd, *J* = 15.8, 6.5, 3.5 Hz, 1H, H-2), 2.29 (s, 3H, PhC*H_3_*), 1.88 (ddd, *J* = 15.9, 9.8, 3.0 Hz, 1H, H-2’), 1.06 (s, 9H, Si-*^t^*Bu). ^13^C NMR (100 MHz, CDCl_3_) *δ* 154.2, 138.4, 135.7, 135.6, 133.2, 133.1, 132.8, 130.1, 130.0, 129.9, 129.4, 128.0, 80.8, 73.5, 72.4, 68.5, 61.6, 28.9, 26.9, 21.3, 19.4. HRMS (ESI) m/z: calcd. for C_30_H_34_O_5_SSiNa^+^ (M + Na)^+^ 557.1788, found 557.1786; ${\left[\alpha \right]}_{D}^{25}$ = +45.7 (c = 1.0, CHCl_3_).

*3-Methylphenyl-1-thio-6-O*-*(tert-butyldiphenylsilyl)-3,4-O-carbonate-2-deoxy-α-D-galactopyranoside***(3b).** The title compound was prepared according to the general procedure of 2-deoxythioglycosides synthesis and purified by flash column chromatography giving colorless oil 43.8 mg, yield 82%; ^1^H NMR (400 MHz, CDCl_3_) *δ* 7.69 (ddd, *J* = 8.0, 5.2, 1.6 Hz, 4H, Ar-H), 7.58–7.34 (m, 6H, Ar-H), 7.27–7.20 (m, 2H, Ar-H), 7.19–7.03 (m, 2H, Ar-H), 5.56 (dd, *J* = 9.8, 6.5 Hz, 1H, H-1), 5.08 (ddd, *J* = 8.4, 4.0, 2.0 Hz, 1H, H-3), 4.93 (dd, *J* = 8.6, 1.6 Hz, 1H, H-4), 4.19 (ddd, *J* = 7.7, 6.0, 1.6 Hz, 1H, H-5), 3.87 (dd, *J* = 10.2, 7.7 Hz, 1H, H-6), 3.80 (dd, *J* = 10.2, 6.1 Hz, 1H, H-6’), 2.68 (ddd, *J* = 15.8, 6.5, 3.5 Hz, 1H, H-2), 2.27 (s, 3H, PhC*H_3_*), 1.92 (ddd, *J* = 15.9, 9.8, 3.0 Hz, 1H, H-2’), 1.08 (s, 9H, Si-*^t^*Bu). ^13^C NMR (100 MHz, CDCl_3_) *δ* 154.2, 138.9, 135.7, 135.6, 133.2, 133.1, 132.9, 132.8, 130.1, 130.0, 129.3, 128.9, 128.9, 128.0, 80.5, 73.4, 72.4, 68.6, 61.5, 29.0, 26.9, 21.4, 19.4. HRMS (ESI) m/z: calcd. for C_30_H_34_O_5_SSiNa^+^ (M + Na)^+^ 557.1788, found 557.1790; ${\left[\alpha \right]}_{D}^{25}$ = +62.2 (c = 1.0, CHCl_3_).

*2-Methylphenyl-1-thio-6-O*-*(tert-butyldiphenylsilyl)-3,4-O-carbonate-*2*-deoxy-α-D-galactopyranoside***(3c).** The title compound was prepared according to the general procedure of 2-deoxythioglycosides synthesis and purified by flash column chromatography giving white solid 43.2 mg, yield 81%; m. p.: 155.7–157.1 °C. ^1^H NMR (400 MHz, CDCl_3_) *δ* 7.64–7.67 (m, 4H, Ar-H), 7.73–7.45 (m, 7H, Ar-H), 7.20–7.09 (m, 2H, Ar-H), 7.03–7.07 (m, 1H, Ar-H), 5.53 (dd, *J* = 9.8, 6.4 Hz, 1H, H-1), 5.07 (ddd, *J* = 8.6, 4.2, 2.0 Hz, 1H, H-3), 4.93 (dd, *J* = 8.7, 1.6 Hz, 1H, H-4), 4.17 (ddd, *J* = 8.0, 6.0, 1.7 Hz, 1H, H-5), 3.83 (dd, *J* = 10.1, 8.0 Hz, 1H, H-6), 3.75 (dd, *J* = 10.1, 6.0 Hz, 1H, H-6’), 2.67 (ddd, *J* = 15.8, 6.5, 3.5 Hz, 1H, H-2), 2.33(s, 3H, PhC*H_3_*), 1.96 (ddd, *J* = 15.9, 9.8, 2.9 Hz, 1H, H-2’), 1.04 (s, 9H, Si-*^t^*Bu). ^13^C NMR (100 MHz, CDCl_3_) *δ* 154.2, 139.4, 135.7, 135.6, 133.2, 132.8, 132.8, 130.3, 130.1, 130.0, 128.0, 128.0, 126.8, 79.8, 73.4, 72.4, 68.6, 61.4, 29.2, 26.9, 20.9, 19.4. HRMS (ESI) m/z: calcd. for C_30_H_34_O_5_SSiNa^+^ (M + Na)^+^ 557.1788, found 557.1793; ${\left[\alpha \right]}_{D}^{25}$ = +43.2 (c = 1.0, CHCl_3_)

*2,4-Methoxylphenyl-1-thio-6-O*-*(tert-butyldiphenylsilyl)-3,4-O-carbonate-2-deoxy-D-galactopyranoside* (**3d**). The title compound was prepared according to the general procedure of 2-deoxythioglycosides synthesis and purified by flash column chromatography giving colorless oil 44.9 mg, yield 82%; ^1^H NMR (400 MHz, CDCl_3_) *δ* 7.77–7.64 (m, 4H, Ar-H), 7.52–7.40 (m, 6H, Ar-H), 7.31–7.33 (m, 1H, Ar-H), 7.03–6.99 (m, 1H, Ar-H), 6.85–6.88 (m, 1H, Ar-H), 5.45 (dd, *J* = 9.8, 6.4 Hz, 1H, H-1), 5.13–5.03 (m, 1H, H-3), 4.96 (dd, *J* = 8.6, 1.6 Hz, 1H, H-4), 4.18 (ddd, *J* = 7.8, 5.8, 1.6 Hz, 1H, H-5), 3.86 (dd, *J* = 10.1, 8.2 Hz, 1H, H-6), 3.76 (dd, *J* = 10.0, 5.9 Hz, 1H, H-6’), 2.68 (ddd, *J* = 15.8, 6.4, 3.5 Hz, 1H, H-2), 2.33 (s, 3H, PhC*H_3_*), 2.28 (s, 3H, PhC*H_3_*), 1.96 (ddd, *J* = 15.9, 9.9, 2.9 Hz, 1H, H-2’), 1.08 (s, 9H, Si-*^t^*Bu). ^13^C NMR (100 MHz, CDCl_3_) *δ* 154.3, 140.0, 138.5, 135.7, 135.6, 134.0, 133.2, 132.8, 131.3, 130.1, 130.0, 128.7, 128.0, 127.5, 80.2, 73.4, 72.4, 68.4, 61.4, 29.1, 26.9, 21.2, 21.0, 19.4. HRMS (ESI) m/z: calcd. for C_31_H_36_O_5_SSiNa^+^ (M + Na)^+^ 571.1945, found 571.1941; ${\left[\alpha \right]}_{D}^{25}$ = +68.4 (c = 1.0, CHCl_3_).

*4-Methoxylphenyl-1-thio-6-O*-*(tert-butyldiphenylsilyl)-3,4-O-carbonate-2-deoxy-α-D-galactopyranside* (**3e**). The title compound was prepared according to the general procedure of 2-deoxythioglycosides synthesis and purified by flash column chromatography giving colorless oil 40.1 mg, yield 73%; ^1^H NMR (400 MHz, CDCl_3_) *δ* 7.69–7.72 (m, 4H, Ar-H), 7.41–7.48 (m, 6H, Ar-H), 7.35–7.38 (m, 2H, Ar-H), 6.74–6.76 (m, 2H, Ar-H), 5.38 (dd, *J* = 9.8, 6.4 Hz, 1H, H-1), 5.06 (ddd *J* = 8.6, 4.4, 2.2Hz, 1H, H-3), 4.91 (dd, *J* = 8.6, 1.6 Hz, 1H, H-4), 4.18 (ddd, *J* = 7.7, 6.1, 1.6 Hz, 1H, H-5), 3.87 (dd, *J* = 10.3, 7.5 Hz, 1H, H-6), 3.80 (dd, *J* = 9.4, 5.3 Hz, 1H, H-6’), 3.77 (s, 3H, PhOC*H*_3_), 2.65 (ddd, *J* = 15.9, 6.5, 3.5 Hz, 1H, H-2), 1.89 (ddd, *J* = 15.8, 9.8, 3.0 Hz, 1H, H-2’), 1.10 (s, 9H, Si-*^t^*Bu).^13^C NMR (100 MHz, CDCl_3_) *δ* 160.2, 154.2, 135.7, 135.7, 135.6, 133.2, 132.8, 130.1, 130.0, 128.0, 123.3, 114.7, 81.2, 73.5, 72.5, 68.5, 61.7, 55.4, 28.9, 27.0, 19.4. HRMS (ESI) m/z: calcd. for C_30_H_34_O_6_SSiNa^+^ (M + Na)^+^ 573.1738, found 573.1739; ${\left[\alpha \right]}_{D}^{25}$ = +90.6 (c = 1.0, CHCl_3_).

*4-tert-Butylphenyl-1-thio-6-O*-*(tert-butyldiphenylsilyl)-3,4-O-carbonate-2-deoxy-α-D-galactopyranoside* (**3f**). The title compound was prepared according to the general procedure of 2-deoxythioglycosides synthesis and purified by flash column chromatography giving colorless oil 46.7 mg, yield 81%; ^1^H NMR (400 MHz, CDCl_3_) *δ* 7.69–7.72 (m, 4H, Ar-H), 7.52–7.38 (m, 6H, Ar-H), 7.41–7.34 (m, 2H, Ar-H), 7.30–7.22 (m, 2H, Ar-H), 5.51 (dd, *J* = 9.7, 6.5 Hz, 1H, H-1), 5.07 (ddd, *J* = 8.6, 4.2, 2.2 Hz, 1H, H-3), 4.92 (dd, *J* = 8.7, 1.7 Hz, 1H, H-4), 4.22 (ddd, *J* = 7.6, 6.2, 1.6 Hz, 1H, H-5), 3.88 (dd, *J* = 10.2, 7.5 Hz, 1H, H-6), 3.82 (dd, *J* = 10.2, 6.2 Hz, 1H, H-6’), 2.67 (ddd, *J* = 15.9, 6.5, 3.5 Hz, 1H, H-2), 1.91 (ddd, *J* = 15.9, 9.8, 3.0 Hz, 1H, H-2’), 1.29 (s, 9H, C-*^t^*Bu), 1.09 (s, 9H, Si-*^t^*Bu).^13^C NMR (100 MHz, CDCl_3_) *δ* 154.2, 151.4, 135.7, 135.6, 133.2, 132.8, 132.6, 130.1, 130.0, 129.6, 128.0, 126.2, 80.7, 73.5, 72.4, 68.5, 61.6, 34.7, 31.3, 29.0, 26.9, 19.4. HRMS (ESI) m/z: calcd. for C_33_H_40_O_5_SSiNa^+^ (M + Na)^+^ 599.2258, found 599.2259; ${\left[\alpha \right]}_{D}^{25}$ = +104.8 (c = 1.0, CHCl_3_).

*Phenyl-1-thio-6-O*-*(tert-butyldiphenylsilyl)-3,4-O-carbonate-2-deoxy-α-D-galactopyranoside* (**3g**). The title compound was prepared according to the general procedure of 2-deoxythioglycosides synthesis and purified by flash column chromatography giving colorless oil 41.6 mg, yield 80%; ^1^H NMR (400 MHz, CDCl_3_) *δ* 7.70 (ddd, *J* = 8.0, 4.0, 1.7 Hz, 4H, Ar-H), 7.53–7.34 (m, 8H, Ar-H), 7.27–7.21 (m, 3H, Ar-H), 5.57 (dd, *J* = 9.7, 6.5 Hz, 1H, H-1), 5.08 (ddd, *J* = 8.4, 4.0, 2.4 Hz, 1H, H-3), 4.92 (dd, *J* = 8.6, 1.6 Hz, 1H, H-4), 4.20 (ddd, *J* = 7.6, 6.2, 1.7 Hz, 1H, H-5), 3.87 (dd, *J* = 10.2, 7.5 Hz, 1H, H-6), 3.81 (dd, *J* = 10.3, 6.2 Hz, 1H, H-6’), 2.69 (ddd, *J* = 15.9, 6.5, 3.5 Hz, 1H, H-2), 1.92 (ddd, *J* = 15.8, 9.8, 3.0 Hz, 1H, H-2’), 1.08 (s, 9H, Si-*^t^*Bu).^13^C NMR (100 MHz, CDCl_3_) *δ* 154.2, 135.7, 135.6, 133.4, 133.2, 132.8, 132.3, 130.1, 130.0, 129.1, 128.0, 80.5, 73.4, 72.4, 68.6, 61.6, 29.0, 26.9, 19.4. HRMS (ESI) m/z: calcd. for C_29_H_32_O_5_SSiNa^+^ (M + Na)^+^ 543.1632, found 543.1642; ${\left[\alpha \right]}_{D}^{25}$ = +67.9 (c = 1.0, CHCl_3_).

*4-Bromophenyl-1-thio-6-O*-*(tert-butyldiphenylsilyl)-3,4-O-carbonate-2-deoxy-α-D-galactopyranoside* (**3h**). The title compound was prepared according to the general procedure of 2-deoxythioglycosides synthesis and purified by flash column chromatography giving colorless oil 46.0 mg, yield 75%; ^1^H NMR (400 MHz, CDCl_3_) *δ* 7.66–7.70 (m, 4H, Ar-H), 7.51–7.39 (m, 6H, Ar-H), 7.36–7.20 (m, 3H, Ar-H), 7.29 (d, *J* = 2.4 Hz, 1H, Ar-H), 5.54 (dd, *J* = 9.8, 6.5 Hz, 1H, H-1), 5.07 (ddd, *J* = 8.6, 4.4, 2.2 Hz, 1H, H-3), 4.90 (dd, *J* = 8.6, 1.7 Hz, 1H, H-4), 4.15–4.19 (m, 1H, H-5), 3.87 (dd, *J* = 10.4, 7.1 Hz, 1H, H-6), 3.79 (dd, *J* = 10.3, 6.3 Hz, 1H, H-6’), 2.69 (ddd, *J* = 15.9, 6.5, 3.5 Hz, 1H, H-2), 1.89 (ddd, *J* = 15.9, 9.9, 3.0 Hz, 1H, H-2’), 1.07 (s, 9H, Si-*^t^*Bu).^13^C NMR (100 MHz, CDCl_3_) *δ* 154.1, 135.7, 135.6, 133.7, 133.6, 133.1, 132.8, 132.6, 132.3, 132.2, 130.1, 130.0, 128.0, 122.3, 80.5, 73.4, 72.3, 68.8, 61.6, 28.8, 26.9, 19.4. HRMS (ESI) m/z: calcd. for C_29_H_31_BrO_5_SSiNa^+^ (M + Na)^+^ 621.0737, found 621.0734; ${\left[\alpha \right]}_{D}^{25}$ = +45.6 (c = 1.0, CHCl_3_).

*2-Bromophenyl-1-thio-6-O*-*(tert-butyldiphenylsilyl)-3,4-O-carbonate-2-deoxy-α-D-galactopyranoside* (**3i**). The title compound was prepared according to the general procedure of 2-deoxythioglycosides synthesis and purified by flash column chromatography giving colorless oil 43.5 mg, yield 71%; ^1^H NMR (400 MHz, CDCl_3_) *δ* 7.64–7.69 (m, 4H, Ar-H), 7.55 (ddd, *J* = 7.9, 2.5, 1.5 Hz, 2H, Ar-H), 7.50–7.35 (m, 6H, Ar-H), 7.17–7.20 (m,1H, Ar-H), 7.06–7.11 (m, 1H, Ar-H), 5.71 (dd, *J* = 9.8, 6.5 Hz, 1H, H-1), 5.12 (ddd, *J* = 8.6, 4.0, 2.2 Hz, 1H, H-3), 4.95 (dd, *J* = 8.6, 1.6 Hz, 1H, H-4), 4.21 (ddd, *J* = 7.8, 6.3, 1.7 Hz, 1H, H-5), 3.85 (dd, *J* = 10.2, 7.5 Hz, 1H, H-6), 3.79 (dd, *J* = 10.2, 6.3 Hz, 1H, H-6’), 2.74 (ddd, *J* = 15.9, 6.6, 3.5 Hz, 1H, H-2), 1.98 (ddd, *J* = 15.8, 9.8, 2.9 Hz, 1H, H-2’), 1.04 (s, 9H, Si-*^t^*Bu).^13^C NMR (100 MHz, CDCl_3_) *δ* 154.2, 135.7, 135.6, 135.2, 133.1, 132.8, 132.0, 130.1, 130.0, 128.6, 128.20, 128.0, 125.2, 79.3, 76.8, 72.3, 68.9, 61.3, 28.6, 26.9, 19.3. HRMS (ESI) m/z: calcd. for C_29_H_31_BrO_5_SSiNa^+^ (M + Na)^+^ 621.0737, found 621.0735; ${\left[\alpha \right]}_{D}^{25}$ = +82.4 (c = 1.0, CHCl_3_).

*4-Fluorophenyl-1-thio-6-O*-*(tert-butyldiphenylsilyl)-3,4-O-carbonate-2-deoxy-α-D-galactopyranoside* (**3j**). The title compound was prepared according to the general procedure of 2-deoxythioglycosides synthesis and purified by flash column chromatography giving colorless oil 39.8 mg, yield 74%; ^1^H NMR (400 MHz, CDCl_3_) *δ* 7.79–7.67 (m, 4H, Ar-H), 7.56–7.40 (m, 8H, Ar-H), 6.90–6.93 (m, 2H, Ar-H), 5.46 (dd, *J* = 9.9, 6.5 Hz, 1H, H-1), 5.07 (ddd, *J* = 8.6, 4.2, 2.0 Hz, 1H, H-3), 4.90 (dd, *J* = 8.7, 1.6 Hz, 1H, H-4), 4.16–4.20 (m, 1H, H-5), 3.87 (dd, *J* = 10.4, 7.2 Hz, 1H, H-6), 3.80 (dd, *J* = 10.3, 6.3 Hz, 1H, H-6’), 2.68 (ddd, *J* = 15.9, 6.5, 3.4 Hz, 1H, H-2), 1.89 (ddd, *J* = 15.8, 9.8, 2.9 Hz, 1H, H-2’), 1.09 (s, 9H, Si-*^t^*Bu).^13^C NMR (100 MHz, CDCl_3_) *δ* 163.0 (d, *J* = 246.7 Hz), 154.1, 135.7, 135.6, 135.1 (d, *J* = 8.2 Hz), 133.1, 132.8, 130.1, 130.0, 128.0, 116.2 (d, *J* = 21.6 Hz), 81.0, 73.4, 72.4, 68.7, 61.7, 28.8, 26.9, 19.4. HRMS (ESI) m/z: calcd. for C_29_H_31_FO_5_SSiNa^+^ (M + Na)^+^ 561.1538, found 561.1541; ${\left[\alpha \right]}_{D}^{25}$ = +86.3 (c = 1.0, CHCl_3_).

*Ethyl-1-thio-6-O*-*(tert-butyldiphenylsilyl)-3,4-O-carbonate-2-deoxy-α-D-galactopyranoside* (**5a**). The title compound was prepared according to the general procedure of 2-deoxythioglycosides synthesis and purified by flash column chromatography giving colorless oil 40.6 mg, yield 86%; ^1^H NMR (400 MHz, CDCl_3_) *δ* 7.67–7.71 (m, 4H, Ar-H), 7.60–7.36 (m, 6H, Ar-H), 5.39 (dd, *J* = 9.3, 6.5 Hz, 1H, H-1), 5.02 (ddd, *J* = 8.4, 4.8, 2.4 Hz, 1H, H-3), 4.86 (dd, *J* = 8.5, 1.7 Hz, 1H, H-4), 4.23–4.01 (m, 1H, H-5), 3.85 (dd, *J* = 10.3, 7.3 Hz, 1H, H-6), 3.78 (dd, *J* = 10.3, 6.4 Hz, 1H, H-6’), 2.67–2.75 (m, 1H, H-2), 2.63–2.49 (m, 2H, C*H_2_*CH_3_), 1.79 (ddd, *J* = 15.8, 9.3, 3.2 Hz, 1H, H-2’), 1.24 (dd, *J* = 13.4, 6.7 Hz, 3H, CH_2_C*H_3_*), 1.08 (s, 9H, Si-*^t^*Bu). ^13^C NMR (100 MHz, CDCl_3_) *δ* 154.4, 135.7, 135.6, 133.2, 132.9, 130.1, 130.0, 128.0, 127.9, 76.7, 73.7, 72.5, 68.0, 61.7, 29.2, 26.9, 24.7, 19.3, 15.0. HRMS (ESI) m/z: calcd. For C_25_H_32_O_5_SsiNa^+^ (M + Na)^+^ 495.1632, found 495.1634; ${\left[\alpha \right]}_{D}^{25}$ = +54.2 (c = 1.0, CHCl_3_).

*Octyl-1-thio-6-O*-*(tert-butyldiphenylsilyl)-3,4-O-carbonate-2-deoxy-α-D-galactopyranoside* (**5b**). The title compound was prepared according to the general procedure of 2-deoxythioglycosides synthesis and purified by flash column chromatography giving colorless oil 43.3 mg, yield 78%; ^1^H NMR (400 MHz, CDCl_3_) *δ* 7.68–7.72 (m, 4H, Ar-H), 7.40–7.50 (m, 6H, Ar-H), 5.35 (dd, *J* = 9.3, 6.5 Hz, 1H, H-1), 5.02 (ddd, *J* = 8.4, 4.5, 2.3 Hz, 1H, H-3), 4.87 (dd, *J* = 8.5, 1.7 Hz, 1H, H-4), 4.13 (ddd, *J* = 7.4, 6.9, 1.6 Hz, 1H, H-5), 3.85 (dd, *J* = 10.2, 7.3 Hz, 1H, H-6), 3.78 (dd, *J* = 10.2, 6.4 Hz, 1H, H-6’), 2.68 (ddd, *J* = 12.9, 8.1, 6.6 Hz, 1H, H-2), 2.57–2.41 (m, 2H), 1.79 (ddd, *J* = 15.8, 9.3, 3.1 Hz, 1H, H-2’), 1.59–1.51 (m, 2H, CH_2_), 1.41–1.12 (m, 10H, CH_2_), 1.08 (s, 9H, Si-*^t^*Bu), 0.89 (dd, *J* = 7.6, 5.7 Hz, 3H, CH_3_). ^13^C NMR (100 MHz, CDCl_3_) *δ* 154.4, 135.7, 135.6, 133.2, 132.9, 130.1, 130.0, 128.0, 127.9, 77.1, 73.7, 72.5, 67.9, 61.7, 31.9, 30.7, 29.9, 29.3, 29.2, 29.2, 29.0, 26.9, 22.8, 19.3, 14.2. HRMS (ESI) m/z: calcd. for C_31_H_44_O_5_SSiNa^+^ (M + Na)^+^ 579.2571, found 579.2556; ${\left[\alpha \right]}_{D}^{25}$ = +51.4 (c = 1.0, CHCl_3_).

*n-Buty-1-thio-6-O*-*(tert-butyldiphenylsilyl)-3,4-O-carbonate-2-deoxy-α-D-galactopyranoside* (**5c**). The title compound was prepared according to the general procedure of 2-deoxythioglycosides synthesis and purified by flash column chromatography giving colorless oil 41.5 mg, yield 83%; ^1^H NMR (400 MHz, CDCl_3_) *δ* 7.48–7.71 (m, 4H, Ar-H), 7.55–7.36 (m, 6H, Ar-H), 5.35 (dd, *J* = 9.3, 6.5 Hz, 1H, H-1), 5.02 (ddd, *J* = 8.5, 4.4, 2.4 Hz, 1H, H-3), 4.87 (dd, *J* = 8.5, 1.7 Hz, 1H, H-4), 4.13 (ddd, *J* = 8.3, 6.4, 1.6 Hz, 1H, H-5), 3.85 (dd, *J* = 10.2, 7.4 Hz, 1H, H-6), 3.77 (dd, *J* = 10.2, 6.3 Hz, 1H, H-6’), 2.69 (ddd, *J* = 14.7, 8.1, 6.7 Hz, 1H, H-2), 2.60–2.46 (m, 2H, CH_2_), 1.78 (ddd, *J* = 15.7, 9.3, 3.1 Hz, 1H, H-2’), 1.60–1.49 (m, 2H, CH_2_), 1.41–1.32 (m, 2H, CH_2_), 1.08 (s, 9H, Si-*^t^*Bu), 0.87 (dd, *J* = 14.6, 6.9 Hz, 3H, CH_3_). ^13^C NMR (100 MHz, CDCl_3_) *δ* 154.4, 135.7, 135.6, 133.2, 132.9, 130.1, 130.0, 128.0, 127.9, 77.1, 73.7, 72.5, 67.9, 61.6, 31.9, 30.4, 29.2, 26.9, 22.0, 19.4, 13.7. HRMS (ESI) m/z: calcd. for C_27_H_36_O_5_SSiNa^+^ (M + Na)^+^ 523.1945, found 523.1945; ${\left[\alpha \right]}_{D}^{25}$ = +36.4 (c = 1.0, CHCl_3_).

*Isobutyl-1-thio-6-O*-*(tert-butyldiphenylsilyl)-3,4-O-carbonate-2-deoxy-α-D-galactopyranoside* (**5d**). The title compound was prepared according to the general procedure of 2-deoxythioglycosides synthesis and purified by flash column chromatography giving colorless oil 41.5 mg, yield 83%; ^1^H NMR (400 MHz, CDCl_3_) *δ* 7.67–7.71(m, 4H, Ar-H), 7.54–7.34 (m, 6H, Ar-H), 5.31 (dd, *J* = 9.3, 6.5 Hz, 1H, H-1), 5.02 (ddd, 8.6, 4.6, 2.4, 1H, H-3), 4.88 (dd, *J* = 8.5, 1.6 Hz, 1H, H-4), 4.19–4.04 (m, 1H, H-5), 3.85 (dd, *J* = 10.2, 7.5 Hz, 1H, H-6), 3.77 (dd, *J* = 10.2, 6.3 Hz, 1H, H-6’), 2.79–2.50 (m, 2H, H-2, C*H*HCH(CH_3_)_2_), 2.42 (dd, *J* = 12.9, 7.2 Hz, 1H, CH*H*CH(CH_3_)_2_), 1.84–1.74 (m, 2H, H-2’, CH_2_C*H*(CH_3_)_2_), 1.08 (s, 9H, Si-*^t^*Bu), 0.94 (dd, *J* = 6.6, 2.7 Hz, 6H, C*H_3_*CHC*H_3_*). ^13^C NMR (100 MHz, CDCl_3_) *δ* 154.4, 135.7, 135.6, 135.6, 133.2, 133.0, 130.1, 130.0, 128.0, 127.9, 77.7, 73.7, 72.5, 67.9, 61.7, 39.7, 29.4, 28.8, 26.9, 22.1, 22.0, 19.4. HRMS (ESI) m/z: calcd. for C_27_H_36_O_5_SSiNa^+^ (M + Na)^+^ 523.1945, found 523.1942; ${\left[\alpha \right]}_{D}^{25}$ = +63.3 (c = 1.0, CHCl_3_).

*sec-Butyl-1-thio-6-O*-*(tert-butyldiphenylsilyl)-3,4-O-carbonate-2-deoxy-α-D-galactopyranoside* (**5e**). The title compound was prepared according to the general procedure of 2-deoxythioglycosides synthesis and purified by flash column chromatography giving colorless oil 41.0 mg, yield 82%; ^1^H NMR (400 MHz, CDCl_3_) *δ* 7.67–7.71 (m, 4H, Ar-H), 7.53–7.37 (m, 6H, Ar-H), 5.43 (dd, *J* = 9.3, 6.5 Hz, 1H, H-1), 5.00–5.05 (m, 1H, H-3), 4.89 (ddd, *J* = 8.3, 6.1, 1.7 Hz, 1H, H-4), 4.13–4.17 (m, 1H, H-5), 3.92–3.81 (m, 1H, H-6), 3.75 (ddd, *J* = 10.1, 6.1, 4.0 Hz, 1H, H-6’), 2.86–2.95 (m, 1H, C*H*CH_2_CH_3_), 2.53 (dddd, *J* = 15.7, 6.2, 3.8, 2.1 Hz, 1H, H-2), 1.81 (dddd, *J* = 15.8, 9.3, 4.5, 3.2 Hz, 1H, H-2’), 1.57–1.41 (m, 2H, CHC*H_2_*CH_3_), 1.29 (d, *J* = 6.9 Hz, 2H, CHC*H_2_*H), 1.20 (d, *J* = 7.0 Hz, 1H, CHCH_2_*H*), 1.08 (s, 9H, Si-*^t^*Bu), 0.91 (dt, *J* = 23.3, 7.4 Hz, 3H, CHCH_2_C*H_3_*). ^13^C NMR (100 MHz, CDCl_3_) *δ* 154.4, 135.7, 135.7, 135.6, 135.6, 133.2, 130.1, 130.0, 128.0, 127.9, 76.6, 73.6, 72.5, 67.8, 61.6, 41.8, 40.0, 30.2, 26.9, 21.3, 19.4, 11.4. ^13^C NMR (100 MHz, CDCl_3_) *δ.*154.4, 135.7, 135.7, 135.6, 135.6, 133.2, 130.1, 130.0, 128.0, 127.9, 75.8, 73.6, 72.5, 67.8, 61.6, 41.2, 39.3, 29.4, 26.9, 21.3, 19.4, 11.3. HRMS (ESI) m/z: calcd. for C_27_H_36_O_5_SSiNa^+^ (M + Na)^+^ 523.1945, found 523.1946; ${\left[\alpha \right]}_{D}^{25}$ = +35.6 (c = 1.0, CHCl_3_).

*tert-Butyl-1-thio-6-O*-*(tert-butyldiphenylsilyl)-3,4-O-carbonate-2-deoxy-α-D-galactopyranoside* (**5f**). The title compound was prepared according to the general procedure of 2-deoxythioglycosides synthesis and purified by flash column chromatography giving colorless oil 40.0 mg, yield 80%; ^1^H NMR (400 MHz, CDCl_3_) *δ* 7.69–7.72 (m, 4H, Ar-H), 7.56–7.37 (m, 6H, Ar-H), 5.56 (dd, *J* = 9.4, 6.5 Hz, 1H, H-1), 5.03 (ddd, *J* = 8.4, 4.8, 2.4 Hz, 1H, H-3), 4.91 (dd, *J* = 8.5, 1.6 Hz, 1H, H-4), 4.13 (ddd, *J* = 7.9, 5.7, 1.6 Hz, 1H, H-5), 3.85 (dd, *J* = 10.0, 8.3 Hz, 1H, H-6), 3.72 (dd, *J* = 10.0, 5.8 Hz, 1H, H-6’), 2.49 (ddd, *J* = 15.7, 6.6, 3.8 Hz, 1H, H-2), 1.81 (ddd, *J* = 15.8, 9.4, 3.1 Hz, 1H, H-2’), 1.31 (s, 9H, C-*^t^*Bu), 1.07 (s, 9H, Si-*^t^*Bu).^13^C NMR (100 MHz, CDCl_3_) *δ* 154.6, 135.7, 135.6, 133.2, 132.8, 130.1, 130.0, 128.0, 127.9, 75.4, 73.5, 72.6, 67.6, 61.2, 44.5, 31.8, 29.4, 26.9, 19.4. HRMS (ESI) m/z: calcd. for C_27_H_36_O_5_SSiNa^+^ (M + Na)^+^ 523.1945, found 523.1949; ${\left[\alpha \right]}_{D}^{25}$ = +40.2 (c = 1.0, CHCl_3_).

*Benzyl-1-thio-6-O*-*(tert-butyldiphenylsilyl)-3,4-O-carbonate-2-deoxy-α-D-galactopyranoside* (**5g**). The title compound was prepared according to the general procedure of 2-deoxythioglycosides synthesis and purified by flash column chromatography giving colorless oil 43.8 mg, yield 82%; ^1^H NMR (400 MHz, CDCl_3_) *δ* 7.70–7.73 (m, 4H, Ar-H), 7.55–7.33 (m, 6H, Ar-H), 7.30–7.10 (m, 5H, Ar-H), 5.21 (dd, *J* = 9.2, 6.6 Hz, 1H, H-1), 5.00 (ddd, *J* = 8.4, 4.6, 2,4 Hz, 1H, H-3), 4.86 (dd, *J* = 8.5, 1.7 Hz, 1H, H-4), 4.14–4.18(m, 1H, H-5), 3.92–3.83 (m, 2H, H-6, PhC*H*H), 3.76 (dd, *J* = 10.3, 6.4 Hz, 1H, H-6’), 3.66 (d, *J* = 13.6 Hz, 1H, PhCH*H*), 2.46 (ddd, *J* = 15.7, 6.6, 3.8 Hz, 1H, H-2), 1.77 (ddd, *J* = 15.8, 9.2, 3.2 Hz, 1H, H-2’), 1.11 (s, 9H, Si-*^t^*Bu).^13^C NMR (100 MHz, CDCl_3_) *δ* 154.3, 137.9, 135.7, 135.6, 133.2, 132.9, 130.1, 130.0, 129.0, 128.7, 128.0, 127.3, 76.0, 73.6, 72.5, 68.2, 61.7, 34.5, 28.8, 27.0, 19.4. HRMS (ESI) m/z: calcd. for C_30_H_34_O_5_SSiNa^+^ (M + Na)^+^ 557.1788, found 557.1777; ${\left[\alpha \right]}_{D}^{25}$ = +140.3 (c = 1.0, CHCl_3_).

*2-Methoxy-2-oxoethyl-1-thio-6-O*-*(tert-butyldiphenylsilyl)-3,4-O-carbonate-2-deoxy-α-D-galactopyranooside* (**5h**). The title compound was prepared according to the general procedure of 2-deoxythioglycosides synthesis and purified by flash column chromatography giving colorless oil 41.2 mg, yield 80%; ^1^H NMR (400 MHz, CDCl_3_) *δ* 7.67–7.70 (m, 4H, Ar-H), 7.54–7.37 (m, 6H, Ar-H), 5.50 (dd, *J* = 9.5, 6.6 Hz, 1H, H-1), 5.05 (ddd, *J* = 8.6, 4.0, 2.2 Hz, 1H, H-3), 4.88 (dd, *J* = 8.6, 1.7 Hz, 1H, H-4), 4.14–4.03 (m, 1H, H-5), 3.84 (dd, *J* = 10.2, 7.2 Hz, 1H, H-6), 3.77 (dd, *J* = 10.2, 6.4 Hz, 1H, H-6’), 3.67 (s, 3H, CH_2_COOC*H_3_*), 3.52 (d, *J* = 15.5 Hz, 1H, C*H*HCOOCH_3_), 3.17 (d, *J* = 15.5 Hz, 1H, CH*H*COOCH_3_), 2.61 (ddd, *J* = 15.8, 6.5, 3.5 Hz, 1H, H-2), 1.76 (ddd, *J* = 15.8, 9.5, 3.1 Hz, 1H, H-2’), 1.08 (s, 9H, Si-*^t^*Bu).^13^C NMR (100 MHz, CDCl_3_) *δ* 170.7, 154.2, 135.7, 135.6, 133.1, 132.8, 130.1, 130.0, 128.0, 77.4, 73.5, 72.3, 68.4, 61.5, 52.7, 31.5, 28.6, 26.9, 19.4. HRMS (ESI) m/z: calcd. for C_26_H_32_O_7_SSiNa^+^ (M + Na)^+^ 539.1530, found 539.1519; ${\left[\alpha \right]}_{D}^{25}$ = +28.0 (c = 1.0, CHCl_3_).

*3-S-(6-O-(tert-Butyldiphenylsilyl)-3,4-O-carbonate-2-deoxy-a-D-galactopyranosyl)-1,2,3,4-di-O-isopropylidene-β-D-glucofuranoside* (**5i**). The title compound was prepared according to the general procedure of 2-deoxythioglycosides synthesis and purified by flash column giving colorless oil 45.9 mg, yield 67%; ^1^H NMR (400 MHz, CDCl_3_) δ 7.66 (ddd, J = 7.8, 3.6, 1.7 Hz, 4H, Ar-H), 7.57–7.35 (m, 6H, Ar-H), 5.93 (d, J = 3.6 Hz, 1H, H-1b), 5.05 (dd, J = 9.6, 6.0 Hz, 1H, H-1a), 4.97–5.00 (m, 1H, H-3a), 4.85 (dd, J = 8.3, 1.7 Hz, 1H, H-4a), 4.45 (dd, J = 3.5, 1.8 Hz, 1H, H-2b), 4.30–4.33 (m, 1H, H-5b), 4.04–4.08 (m, 1H, H-5a), 3.92–3.65 (m, 5H, H-6a, 6’a, H-4b, H-7b, 7’b), 3.60 (dd, J = 4.5, 2.0 Hz, 1H, H-3b), 2.41 (ddd, J = 15.7, 5.6, 4.2 Hz, 1H, H-2a), 1.89 (ddd, J = 15.7, 6.4, 3.7 Hz, 1H, H-2’a), 1.57 (s, 3H, CH_3_), 1.49 (s, 3H, CH_3_), 1.45 (s, 3H, CH_3_), 1.31 (s, 3H, CH_3_), 1.06 (s, 9H, Si-^t^Bu).^13^C NMR (100 MHz, CDCl_3_) δ 154.4, 135.7, 135.6, 133.2, 132.9, 130.1, 130.0, 128.0, 112.3, 106.4, 95.21, 83.6, 83.2, 77.9, 73.8, 73.5, 72.1, 67.9, 67.7, 61.7, 47.5, 31.6, 29.7, 27.5, 27.1, 27.0, 26.6, 19.4. HRMS (ESI) m/z: calcd. for C_35_H_47_O_10_SSi^+^ (M + H)^+^ 687.2654, found 687.2670; ${\left[\alpha \right]}_{D}^{25}$ = +27.2 (c = 1.0, CHCl_3_).

*6-S-(6-O-(tert-butyldiphenylsilyl)-3,4-O-carbonate-2-deoxy-a-D-galactopyranosyl)-,2,3,4,6-tetra-O-acetyl-β-D-glucopyranoside* (**5j**). The title compound was prepared according to the general procedure of 2-deoxythioglycosides synthesis and purified by flash column chromatography giving white solid 53.0 mg, yield 69%; m.p.: 144.6–146.3 °C. ^1^H NMR (400 MHz, CDCl_3_) *δ* 7.84–7.56 (m, 4H, Ar-H), 7.65–7.35 (m, 6H, Ar-H), 5.56 (dd, *J* = 9.8, 6.4 Hz, 1H, H-1a), 5.12–5.17(m, 1H, H-3b), 5.09–5.02 (m, 2H, H-3a, H-2b), 5.02–4.97 (m, 2H, H-4a, H-4b), 4.61 (d, *J* = 10.1 Hz, 1H, H-1b), 4.11 (ddd, *J* = 8.0, 6.8, 1.5 Hz, 1H, H-5), 4.00 (dd, *J* = 12.5, 4.8 HZ, 1H, H-6a), 3.90 (dd, *J* = 12.5, 2.2 Hz, 1H, H-6’a), 3.90–3.79 (m, 2H, H-6, 6’b), 3.60 (ddd, *J* = 10.1, 4.7, 2.2 Hz, 1H, H-5b), 2.56 (ddd, *J* = 15.8, 6.4, 3.3 Hz, 1H, H-2a), 2.20–1.97 (m, 9H, OAc), 1.89–1.82 (m, 4H, H-2’a, OAc), 1.07 (s, 9H, Si-*^t^*Bu). ^13^C NMR (100 MHz, CDCl_3_) *δ* 170.6, 170.3, 169.5, 169.2, 154.1, 135.7, 135.6, 133.1, 132.7, 130.1, 130.0, 128.0, 83.3, 77.6, 76.4, 73.9, 73.1, 72.02, 71.1, 68.2, 67.9, 61.8, 60.7, 29.0, 26.9, 20.8, 20.7, 19.4. HRMS (ESI) m/z: calcd. for C_37_H_46_O_14_SSiNa^+^ (M + Na)^+^ 797.2270, found 797.2265; ${\left[\alpha \right]}_{D}^{25}$ = +21.1 (c = 1.0, CHCl_3_).

*Estronyl-1-thio-6-O*-*(tert-butyldiphenylsilyl)-3,4-O-carbonate-2-deoxy-α-D-galactopyranoside* (**7a**). The title compound was prepared according to the general procedure of 2-deoxythioglycosides synthesis and purified by flash column chromatography giving colorless oil 54.3 mg, yield 78%; ^1^H NMR (400 MHz, CDCl_3_) *δ* 7.80–7.64 (m, 4H, Ar-H), 7.52–7.36 (m, 6H, Ar-H), 7.26–7.10 (m, 3H, Ar-H), 5.51 (dd, *J* = 9.8, 6.5 Hz, 1H, H-1), 5.08 (ddd, *J* = 8.6, 4.1, 2.4 Hz, 1H, H-3), 4.94 (dd, *J* = 8.6, 1.6 Hz, 1H, H-4), 4.20 (ddd, *J* = 7.6, 5.9, 1.6 Hz, 1H, H-5), 3.88 (dd, *J* = 10.2, 7.8 Hz, 1H, H-6), 3.79 (dd, *J* = 10.2, 6.0 Hz, 1H, H-6’), 2.83–2.74 (m, 2H, PhC*H_2_*), 2.68 (ddd, *J* = 15.9, 6.5, 3.4 Hz, 1H, H-2), 2.52 (dd, *J* = 18.9, 8.6 Hz, 1H, PhC*H*), 2.34–2.38 (m, 1H, COC*H*H), 2.22–2.23 (m, 1H, COCH*H*), 2.23–2.04 (m, 3H, CH_2,_ C*H*H), 2.04–1.86 (m, 3H, H-2’, CH_2_), 1.71–1.53 (m, 3H, CH_2_, CH*H*), 1.50–1.37 (m, 2H, C*H*C*H*), 1.09 (s, 9H, Si-*^t^*Bu), 0.91 (s, 3H, CH_3_). ^13^C NMR (100 MHz, CDCl_3_) *δ* 220.9, 154.2, 140.1, 137.5, 135.7, 135.6, 133.3, 133.2, 132.8, 130.3, 130.1, 130.0, 129.9, 128.0, 126.2, 80.7, 73.4, 72.4, 68.5, 61.5, 50.6, 48.1, 44.4, 38.0, 36.0, 31.7, 29.3, 28.9, 26.9, 26.4, 25.7, 21.7, 19.4, 13.9. HRMS (ESI) m/z: calcd. For C_41_H_48_O_5_SsiNa^+^ (M + Na)^+^ 719.2833, found 719.2830; ${\left[\alpha \right]}_{D}^{25}$ = +114.6 (c = 1.0, CHCl_3_).

*L-Menthyl-1-thio-6-O-(tert-butyldiphenylsilyl)-3,4-O-carbonate-*2*-deoxy-α-D-galactopyranoside***(7b).** The title compound was prepared according to the general procedure of 2-deoxythioglycosides synthesis and purified by flash column chromatography giving colorless oil 34.9 mg, yield 60%; ^1^H NMR (400 MHz, CDCl_3_) *δ* 7.83–7.55 (m, 4H, Ar-H), 7.55–7.34 (m, 6H, Ar-H), 5.32 (dd, *J* = 9.7, 6.4 Hz, 1H, H-1), 5.06 (ddd, *J* = 8.7, 3.1 Hz, 1H, H-3), 5.01–5.08 (m, 1H, H-4), 4.14 (ddd, *J* = 9.5, 5.5, 1.5 Hz, 1H, H-5), 3.84–3.88 (m, 1H, H-6), 3.74 (dd, *J* = 9.6, 5.5 Hz, 1H, H-6’), 3.36–3,37 (m, 1H, SC*H*), 2.55 (ddd, *J* = 15.8, 6.4, 3.5 Hz, 1H, H-2), 1.88–1.95 (m, 2H, CH, CH), 1.83 (ddd, *J* = 15.8, 9.7, 2.8 Hz, 1H, H-2’), 1.77–1.69 (m, 2H, CH_2_), 1.52–1.43 (m, 1H, C*H*H), 1.25–1.13 (m, 1H, CH*H*), 1.08 (s, 9H, Si-*^t^*Bu), 1.05–0.99 (m, 2H, CH, C*H*H), 0.88–0.92 (m, 4H, CH_3,_ CH*H*), 0.83 (d, *J* = 6.6 Hz, 3H, CH_3_), 0.67 (d, *J* = 6.5 Hz, 3H, CH_3_).^13^C NMR (100 MHz, CDCl_3_) *δ* 154.5, 135.6, 135.5, 133.2, 132.8, 130.1, 130.0, 128.0, 127.9, 75.4, 73.5, 72.6, 67.5, 61.0, 48.3, 44.5, 40.3, 35.4, 29.9, 29.5, 26.9, 26.6, 26.4, 22.2, 21.0, 20.4, 19.4. HRMS (ESI) m/z: calcd. for C_33_H_46_O_5_SSiNa^+^ (M + Na)^+^ 605.2727, found 605.2728; ${\left[\alpha \right]}_{D}^{25}$ = +56.7 (c = 1.0, CHCl_3_).

*Zingerone-1-thio-6-O*-*(tert-butyldiphenylsilyl)-3,4-O-carbonate-2-deoxy-α-D-galactopyranoside* (**7c**). The title compound was prepared according to the general procedure of 2-deoxythioglycosides synthesis and purified by flash column chromatography giving colorless oil 41.2 mg, yield 72%; ^1^H NMR (400 MHz, CDCl_3_) *δ* 7.81–7.63 (m, 4H), 7.52–7.34 (m, 6H, Ar-H), 7.33–7.14 (m, 1H, Ar-H), 6.69 (d, *J* = 1.6 Hz, 1H, Ar-H), 6.60 (dd, *J* = 7.8, 1.7 Hz, 1H, Ar-H), 5.61 (dd, *J* = 9.6, 6.5 Hz, 1H, H-1), 5.08–5.10 (m, 1H, H-3), 4.95 (dd, *J* = 8.7, 1.6 Hz, 1H, H-4), 4.22 (ddd, *J* = 7.9, 5.9, 1.6 Hz, 1H, H-5), 3.94–3.78 (m, 4H, H-6, OMe), 3.70 (dd, *J* = 10.0, 6.0 Hz, 1H, H-6’), 2.82–2.86 (m, 2H, C*H_2_*CH_2_), 2.77–2.63 (m, 3H, H-2, CH_2_C*H_2_*), 2.14 (s, 3H, CH_2_COC*H_3_*), 1.92 (ddd, *J* = 15.8, 9.6, 3.0 Hz, 1H, H-2’), 1.07 (s, 9H, Si-*^t^*Bu).^13^C NMR (100 MHz, CDCl_3_) *δ* 207.7, 158.4, 154.3, 143.3, 135.7, 135.6, 134.5, 133.2, 132.8, 130.0, 129.9, 128.0, 121.0, 118.1, 111.3, 78.6, 73.5, 72.5, 68.23, 61.4, 55.9, 45.0, 30.2, 29.8, 28.9, 26.9, 19.3. HRMS (ESI) m/z: calcd. for C_34_H_40_O_7_SSiNa^+^ (M + Na)^+^ 643.2165, found 643.2154; ${\left[\alpha \right]}_{D}^{25}$ = +99.8 (c = 1.0, CHCl_3_).

## Figures and Tables

**Table 1:** Condition optimization for the 2-deoxythioglycosylation ^a^.

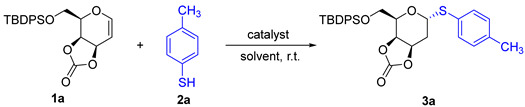			
Entry	Catalyst	Solvent	Yield (%) ^b^
1	Cu(OTf)_2_	DCM	32
2	Hg(OTf)_2_	DCM	59
3	Yb(OTf)_3_	DCM	30
4	Fe(OTf)_3_	DCM	64
5	Ni(OTf)_2_	DCM	N.R.
6	Sc(OTf)_3_	DCM	50
7	Dy(OTf)_3_	DCM	N.R.
8	AgOTf	DCM	80
9	AgOTf	toluene	43
10	AgOTf	THF	N.R.
11	AgOTf	MeCN	N.R.
12	AgOTf	DMC	57
13	AgOTf	DEC	33
14	AgOTf	DMF	N.R.
15	AgOTf	DMSO	N.R.

^a^ Unless otherwise specified, all reactions were carried out with 0.1 mmol of **1a**, 0.11 mmol of **2a**, and 10 mol% catalyst in 2 mL solvent, at room temperature for 24 h under N_2_ atmosphere. ^b^ Isolated yield, α/β > 20:1 by ^1^H NMR. N.R. = No Reaction.

## Data Availability

Data are included within the manuscript or the supplementary data file.

## Supplementary Materials

The following supporting information can be downloaded at: https://www.mdpi.com/article/10.3390/molecules27227979/s1, general information, NMR spectra and X-ray crystal structure and data, Figure S1: Synthesis of estrone thiol, Figure S2: Synthesis of L-menthol thiol, Figure S3: Synthesis of zingerone thiol, Figure S4: ORTEP drawing of compound 3c showing thermal ellipsoids at the 50% probability level (CCDC: 2212881), Table S1: Crystal data and structure refinement for **3c**. The data for known compounds were checked in comparison with literature for consistency [38,39,40,41].

## Sample Availability

Samples of the compounds are available from the authors.
