# Supplementary material for: Stereoselective Synthesis of 2-Deoxythiosugars from Glycals
Source: Molecules. 2022 Nov 17;27(22):7979. doi: 10.3390/molecules27227979 (PMC9696349; doi:10.3390/molecules27227979)
Supplement: Supplementary file 1 [file molecules-27-07979-s001.zip › molecules-2035297-supplementary.pdf]

Supporting Information

Stereoselective Synthesis of 2-Deoxythiosugars from Glycals

Xueying You [1], Yifei Cai [1], Chenyu Xiao [1], Lijuan Ma [1], Yong Wei [2], Tianpeng Xie [2], Lei Chen [2,]* and Hui Yao [1,]*

[1] Hubei Key Laboratory of Natural Products Research and Development, Key Laboratory of Functional Yeast (China National Light Industry), College of Biological and Pharmaceutical Sciences, China Three Gorges University, Yichang 443002, Hubei, P. R. China

[2] Yichang Humanwell Pharmaceutical Co., Ltd., Yichang 443000, Hubei, P. R. China

* Correspondence: yaohui@ctgu.edu.cn (H.Y.); chenleiyf@renfu.com.cn (L.C.)

Supporting Information

Table of Contents **Page**

Section A: General Information **S3**

Section B: General Experimental Procedures **S4**

Section C: NMR Spectra **S6**

Section D: X-ray Crystal Structure and Data **S34**

Section A: General Information

Unless otherwise specified, all reactions for 2-deoxythioglycosides synthesis were performed in sealed Schlenk tubes under N_2 atmosphere. Starting materials, reagents, catalysts and solvents were all purchased commercially and used as received. Thin layer chromatography (TLC) with TLC silica gel GF254 plate was used to check reaction progress. UV light was used to visualize compounds on TLC plates under 254 nm. Flash column chromatography with silica gel (200-300 mesh) and gradient solvent system (petroleum ether/ethyl acetate as eluent) was used to isolate products. ^{1}H and ^{13}C NMR spectra were obtained using 400 MHz Bruker Avance 400 spectrometer. All ^{1}H-NMR chemical shifts were recorded relative to the TMS peak at 0.00 ppm or the solvent residual peak ($CDCl_3$ at 7.26 ppm). ^{13}C NMR chemical shifts are reported relative to the TMS peak at 0.0 ppm or the solvent residual peak ($CDCl_3$ at 77.16 ppm). Multiplicities were reported as: s (singlet), d (doublet), t (triplet), q (quartet), quin (quintuplet), sext (sextet), sep (septet), m (multiplet or unsolved), br s (broad singlet), dd (doublet of doublets), doublet of triplets (dt), triplet of doublets (td) or doublet of doublet of doublets (ddd). The number of protons (n) corresponding to a resonance signal was indicated by nH and spin-spin coupling constants (*J* value) recorded in Hz. Characterization data for known compounds were checked in comparison with literature for consistency and not presented in this report.

Section B: General Experimental Procedures

Synthesis of estrone thiol (6a)

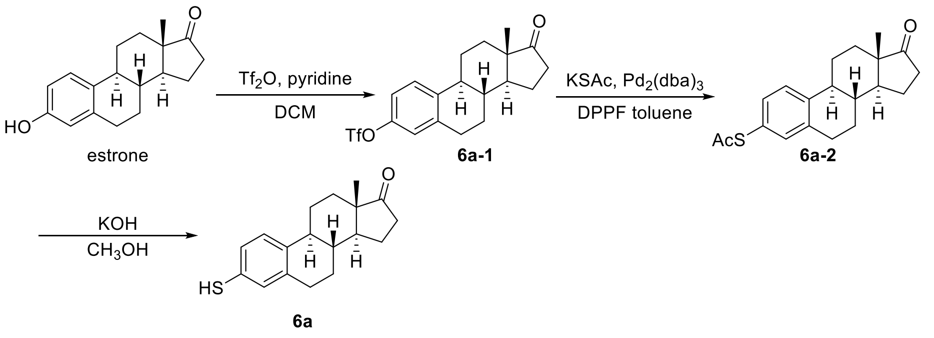

Figure S1. Synthesis of estrone thiol.

Estrone (3.7 mmol) and pyridine (7.4 mmol) were added to DCM (10 mL), trifluoromethanesulfonic anhydride (3.7 mmol) was added at 0 °C under nitrogen gas, and the reaction solution was warmed to room temperature and stirred for 2 h. The resulting solution was washed successively with HCl (2N, 10 ml x2), dried over Na_2SO_4, and subjected to column chromatography on silica gel to obtain the desired product **6a-1** with a yield of 89%.

To a mixture of **6a-1** (1.24 mmol), $Pd_2(dba)_3$ (0.031mmol), dppf (0.062 mmol) and KSAc (2.48 mmol) were added 10 mL anhydrous toluene under nitrogen gas. The reaction was stirred at 110 °C for 24 hours. Then the reaction mixture was quenched with water, extracted by ethyl acetate, dried over Na_2SO_4 and subjected to column chromatography on silica gel to obtain the desired product **6a-2** with a yield of 39%.

To a solution of **6a-2** (0.48 mmol) in 5 mL methanol, KOH (1.44 mmol) was added and stirred at room temperature for 1 hour. The reaction was quenched with saturated ammonium chloride and extracted with dichloromethane, dried over Na_2SO_4 and submitted to column chromatography on silica gel to obtain the desired product **6a** with a yield of 80%. ^{1}H NMR (400 MHz, $CDCl_3$) δ 7.18 (dd, J = 8.1, 1.0 Hz, 1H), 7.13–7.03 (m, 2H), 3.38 (s, 1H), 2.88 (dd, J = 9.0, 4.2 Hz, 2H), 2.64–2.47 (m, 1H), 2.47–2.35 (m, 1H), 2.23–2.09 (m, 1H), 2.09–1.93 (m, 3H), 1.72–1.38 (m, 6H), 0.93 (s, 3H). ^{13}C NMR (100 MHz, $CDCl_3$) δ 220.7, 137.6, 130.1, 127.4, 127.2, 126.2, 50.4, 48.0, 44.1, 38.1, 35.9, 31.5, 29.2, 26.4, 25.7, 21.6, 13.9. The data was consistent with the reported literature[1-3].

Synthesis of L-menthol thiol (6b)

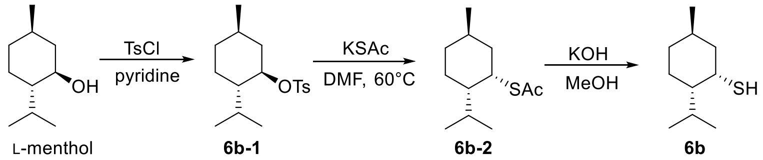

Figure S2. Synthesis of L-menthol thiol.

p-Toluenesulfonyl chloride (10.8 mmol) was added to a solution of L-menthol (6.4 mmol) in pyridine (10 mL) at 0 °C under nitrogen gas. After10 minutes, the mixture was warmed to room temperature and stirred for 16 hours. The resulting solution was washed successively with HCl (2N), dried over Na_2SO_4, and subjected to column chromatography on silica gel to obtain the desired product **6b-1** with a yield of 78%.

To the mixture of **6b-1** (8.4 mmol), KSAc (16.8mmol) was added DMF (15 mL) and stirred at 60 °C for 36

h. The reaction was quenched by water and extracted with ethyl acetate, dried over Na_2SO_4 and submitted to column chromatography on silica gel to provide the desired product **6b-2** with a yield of 62%.
To the solution of **6b-2** (5.2 mmol) in 15 mL methanol, was added to KOH (15.6 mmol) and stirred at room temperature for 1 hour. The reaction was quenched with saturated ammonium chloride and extracted with dichloromethane, dried over Na_2SO_4 and subjected to column chromatography on silica gel to obtain the desired product **6b** with a yield of 77%. ^{1}H NMR (400 MHz, $CDCl_3$) δ 3.23-3.26 (m, 1H), 2.30-2.36 (m, 1H), 1.86-1.87 (m, 1H), 1.83–1.65 (m, 4H), 1.25–1.12 (m, 3H), 1.00 (d, J = 6.6 Hz, 3H), 0.97–0.85 (m, 7H). ^{13}C NMR (100 MHz, $CDCl_3$) δ 52.6, 48.8, 39.8, 35.5, 30.0, 26.2, 25.9, 22.2, 21.2, 20.6. The data was consistent with the reported literature[4].

Synthesis of zingerone thiol (6c)

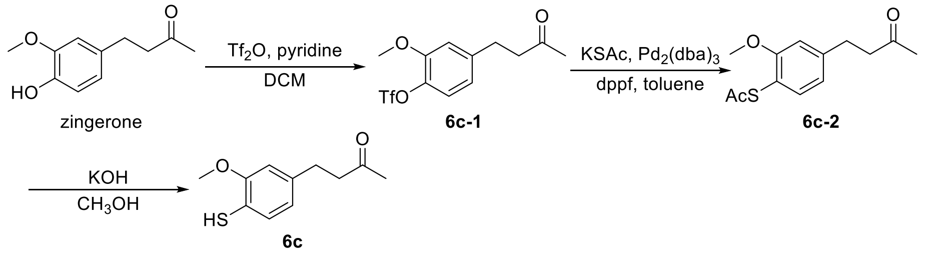

Figure S3. Synthesis of zingerone thiol.

To the mixture of zingerone (3.7 mmol) and pyridine (7.4 mmol) were added trifluoromethanesulfonic anhydride (3.7 mmol) in DCM (10 mL) at 0 °C under nitrogen gas. Then the reaction solution was warmed to room temperature and stirred for 2 h. The resulting solution was washed successively with HCl (2N, 10 ml x2), dried on Na_2SO_4, and subjected to column chromatography on silica gel to obtain the desired product **6c-1** with a yield of 90%.
To a mixture of **6c-1** (1.24 mmol), $Pd_2(dba)_3$ (0.031mmol), dppf (0.062 mmol) and KSAc (2.48mmol) were added 10 mL anhydrous toluene under nitrogen gas. The reaction was stirred at 110 °C for 24 hours. Then the reaction mixture was quenched with water and extracted by ethyl acetate, dried over Na_2SO_4, and subjected to column chromatography on silica gel to give the desired product **6c-1** with a yield of 33%.
To a solution of **6c-2** (0.4 mmol) in 5 mL methanol, KOH (1.22 mmol) was added and stirred at room temperature for 1 hour. The reaction was quenched with saturated ammonium chloride, extracted with dichloromethane, dried over Na_2SO_4 and subjected to column chromatography on silica gel to generate the desired product **6c** with a yield of 81%. ^{1}H NMR (400 MHz, $CDCl_3$) δ 7.20–7.12 (m, 1H), 6.70 (d, J = 7.2 Hz, 2H), 3.90 (s, 3H), 3.74 (s, 1H), 3.10–2.81 (m, 2H), 2.81–2.68 (m, 2H), 2.16 (s, 3H). ^{13}C NMR (100 MHz, $CDCl_3$) δ 207.8, 155.0, 139.9, 129.5, 120.8, 117.6, 111.0, 55.8, 45.2, 30.2, 29.7. HRMS (ESI) m/z: calcd. for $C_{11}H_{14}O_2SNa^+$ (M + Na)$^+$ 233.0607, found 233.0614.

Section C: NMR Spectra

^{1}H NMR of 6a (400 MHz, $CDCl_3$)

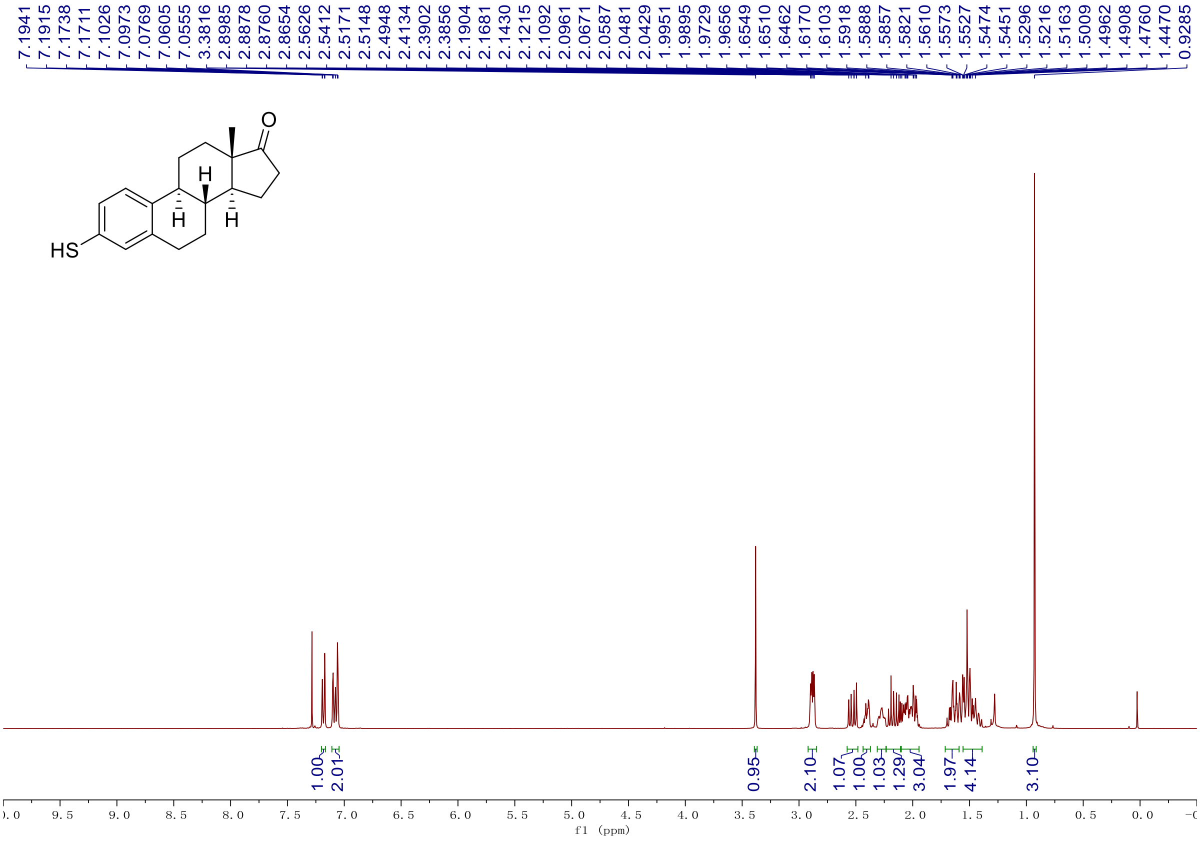

^{13}C NMR of 6a (100 MHz, $CDCl_3$)

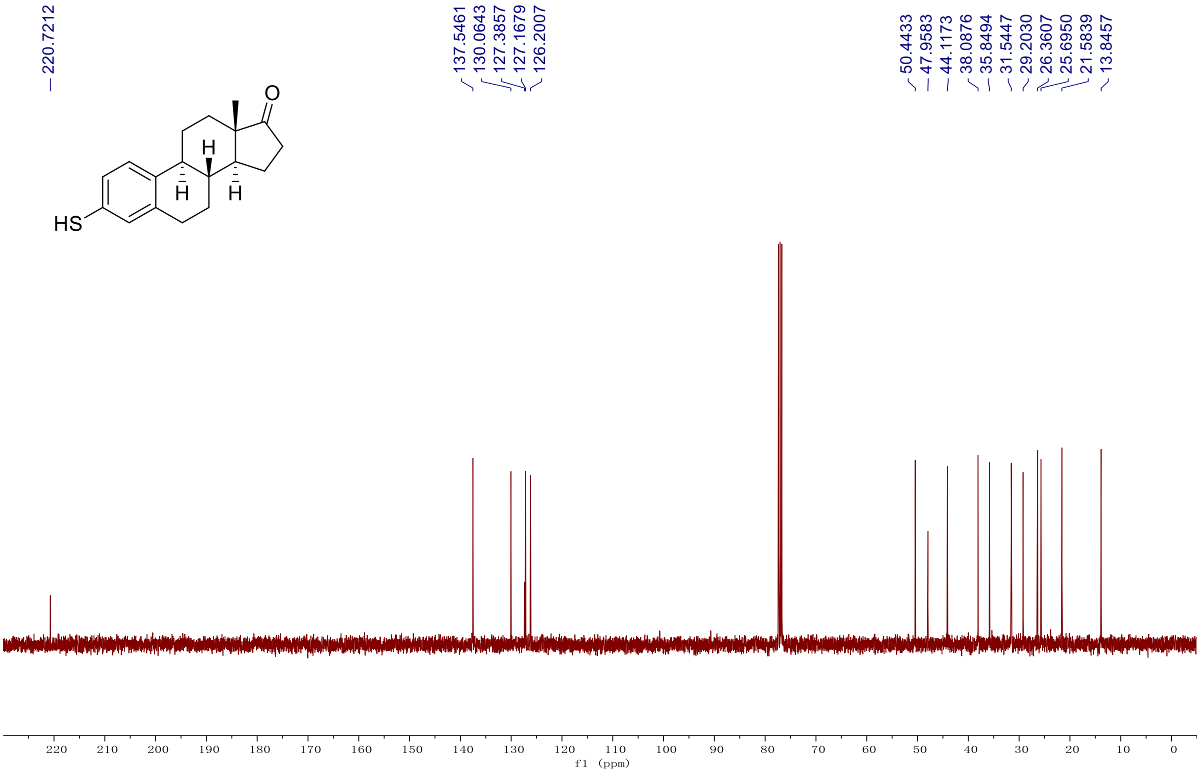

^{1}H NMR of 6b (400 MHz, $CDCl_3$)

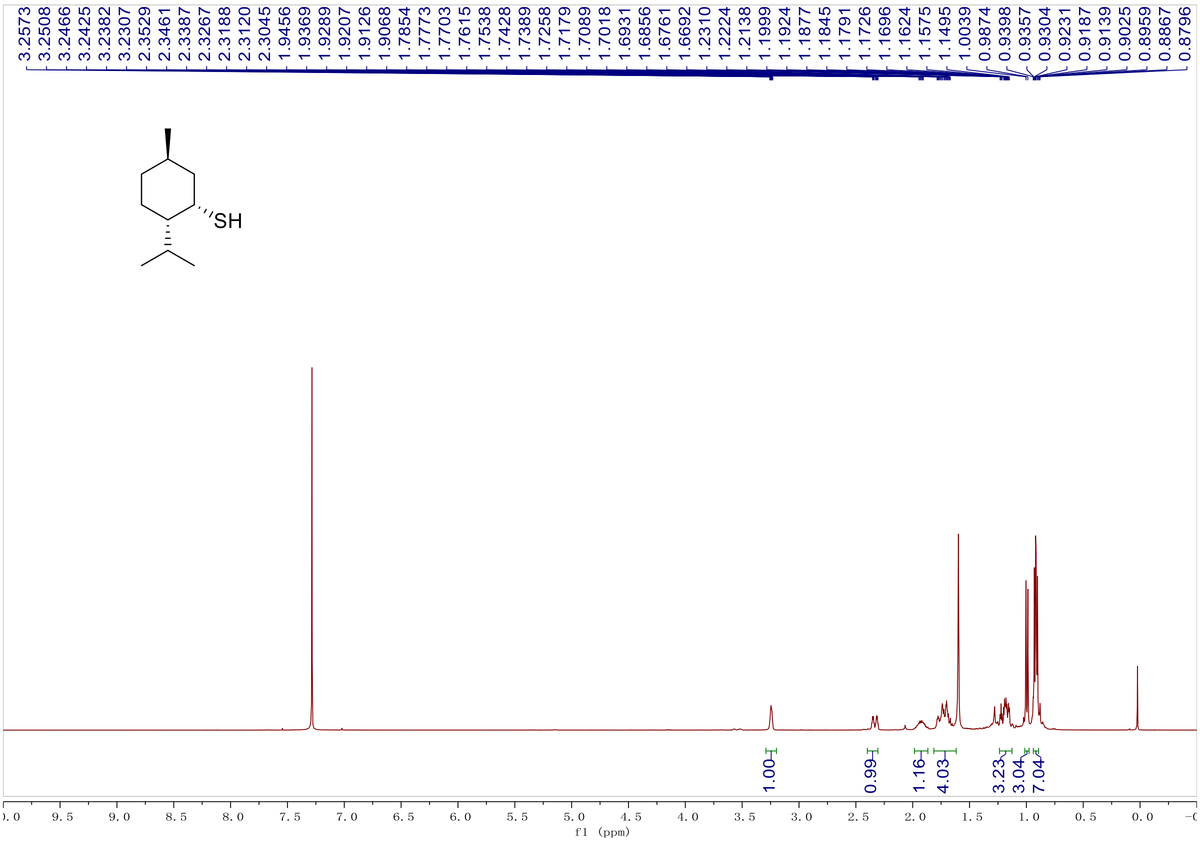

^{13}C NMR of 6b (100 MHz, $CDCl_3$)

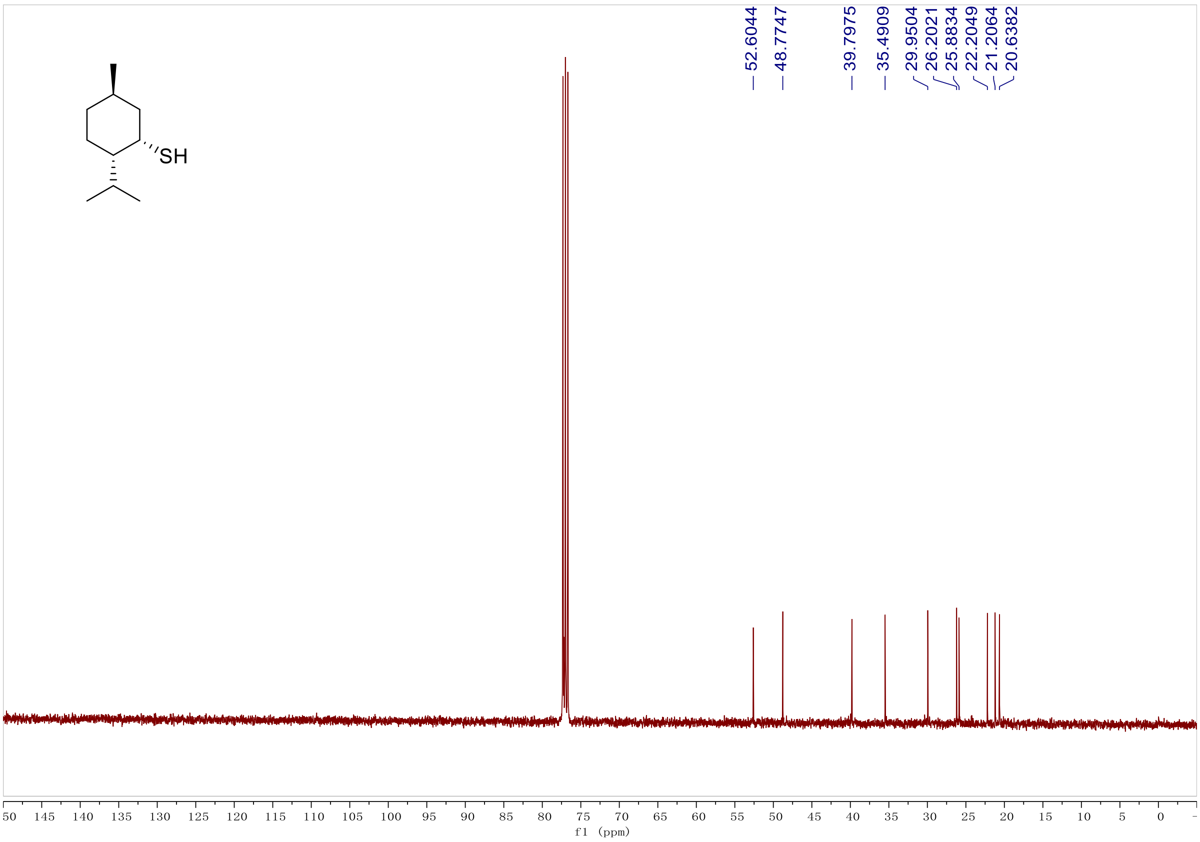

^{1}H NMR of 6c (400 MHz, $CDCl_3$)

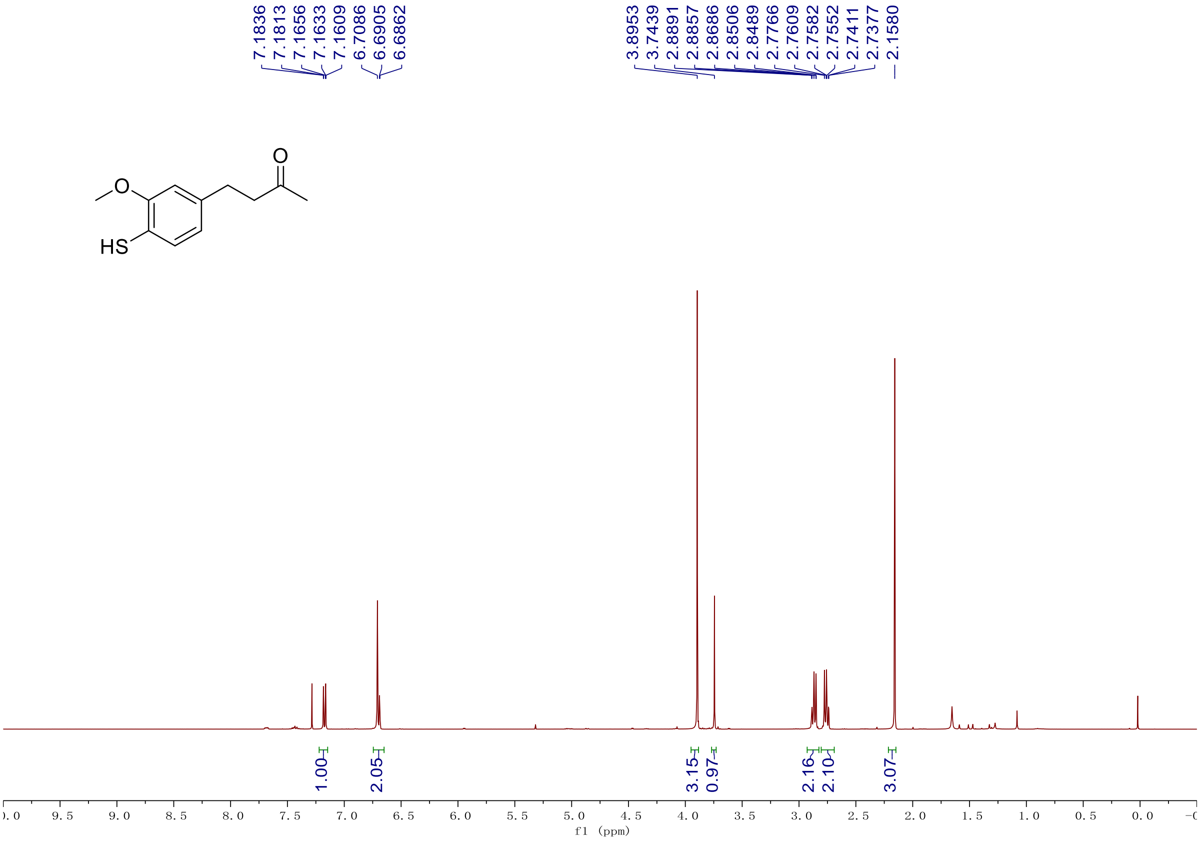

^{13}C NMR of 6c (100 MHz, $CDCl_3$)

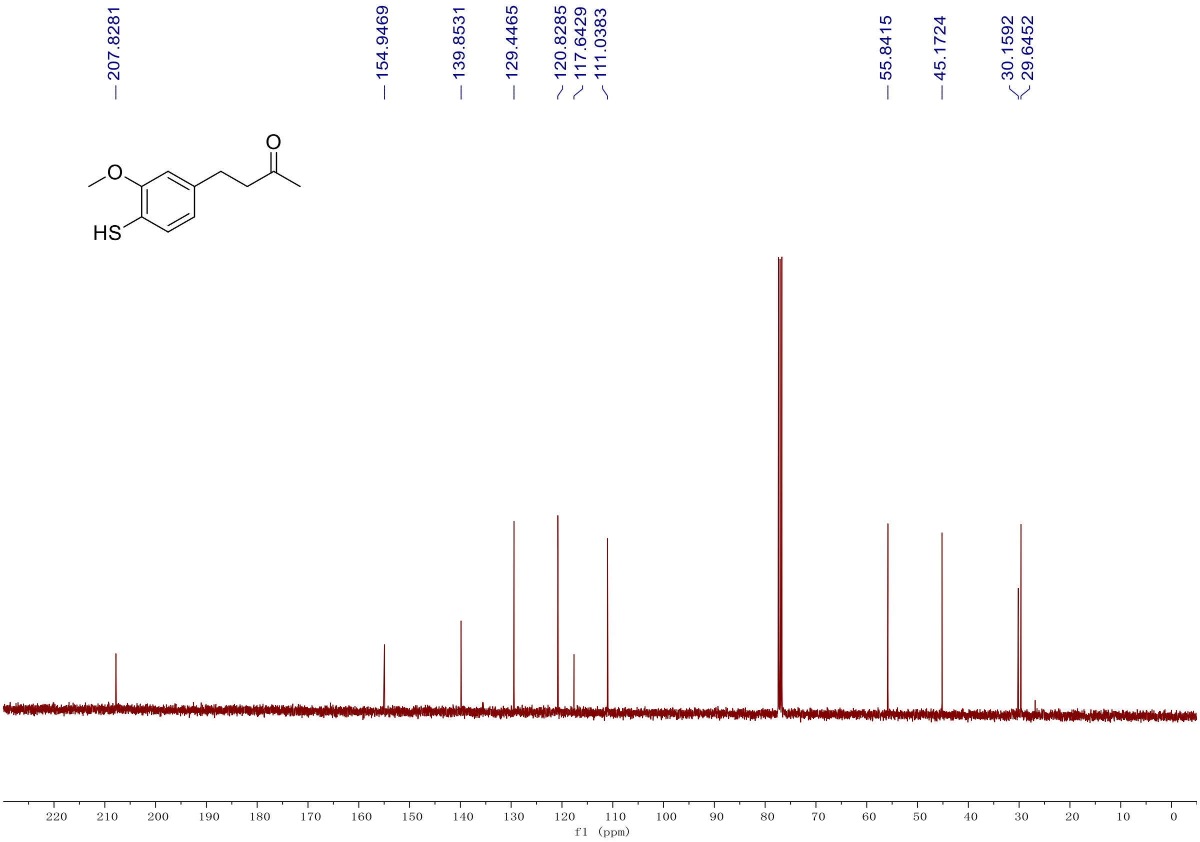

^{1}H NMR of 3a (400 MHz, $CDCl_3$)

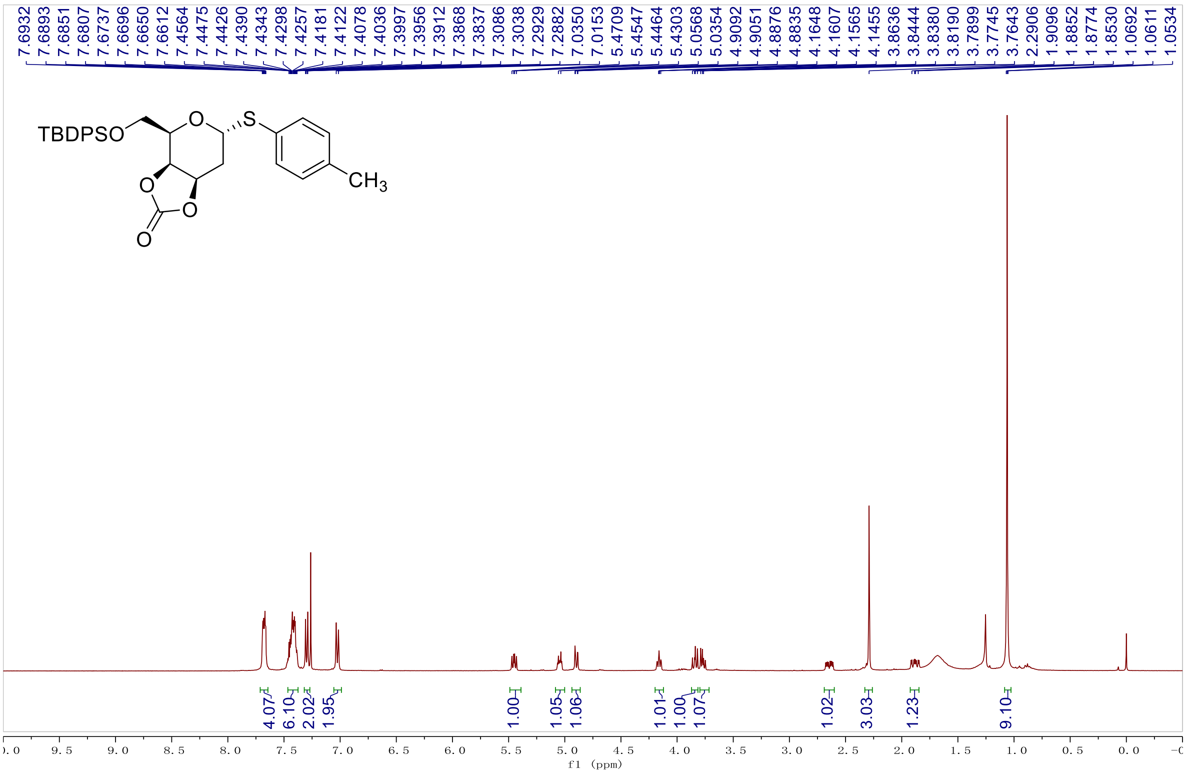

^{13}C NMR of 3a (100 MHz, $CDCl_3$)

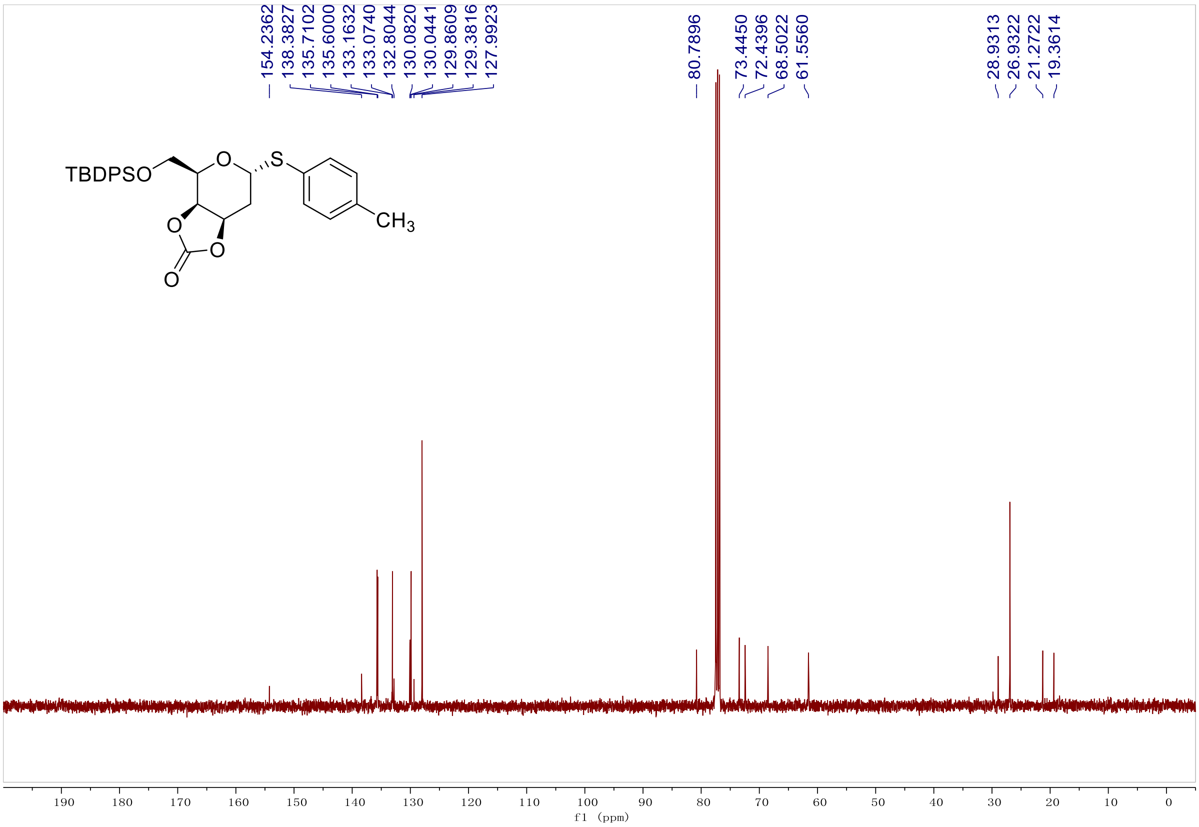

2D COSY NMR of 3a

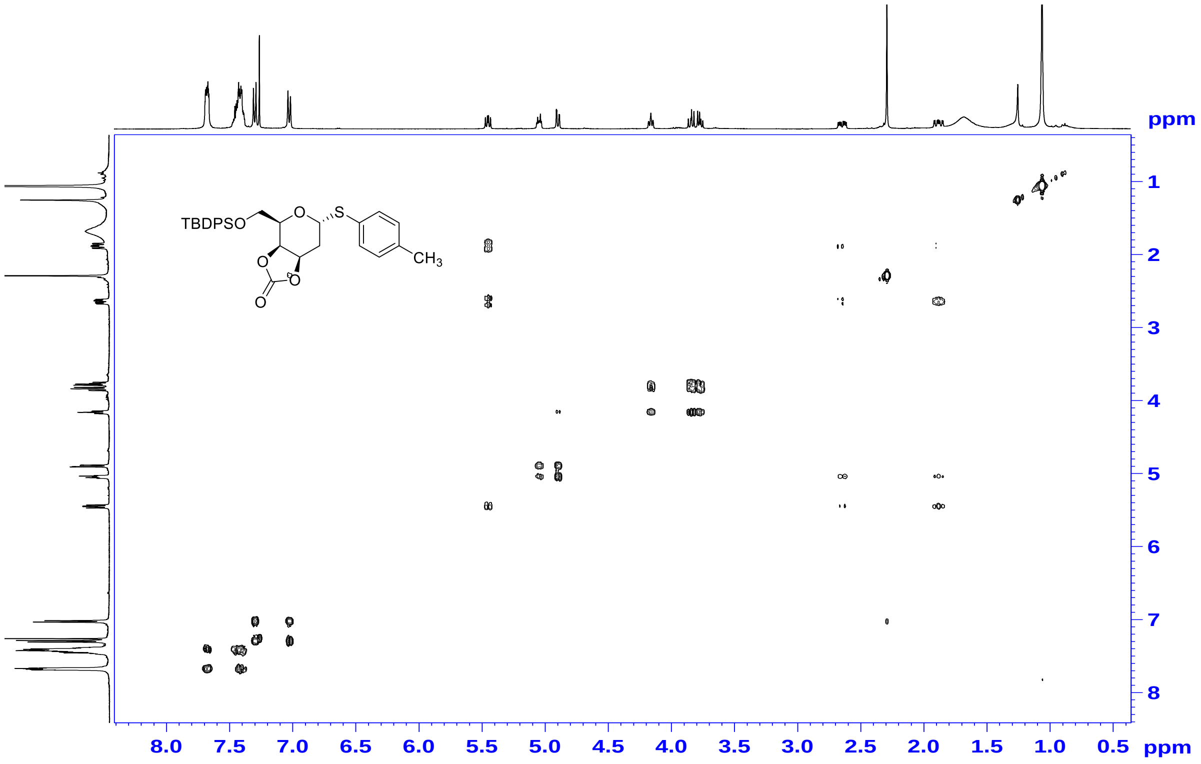

2D HSQC NMR of 3a

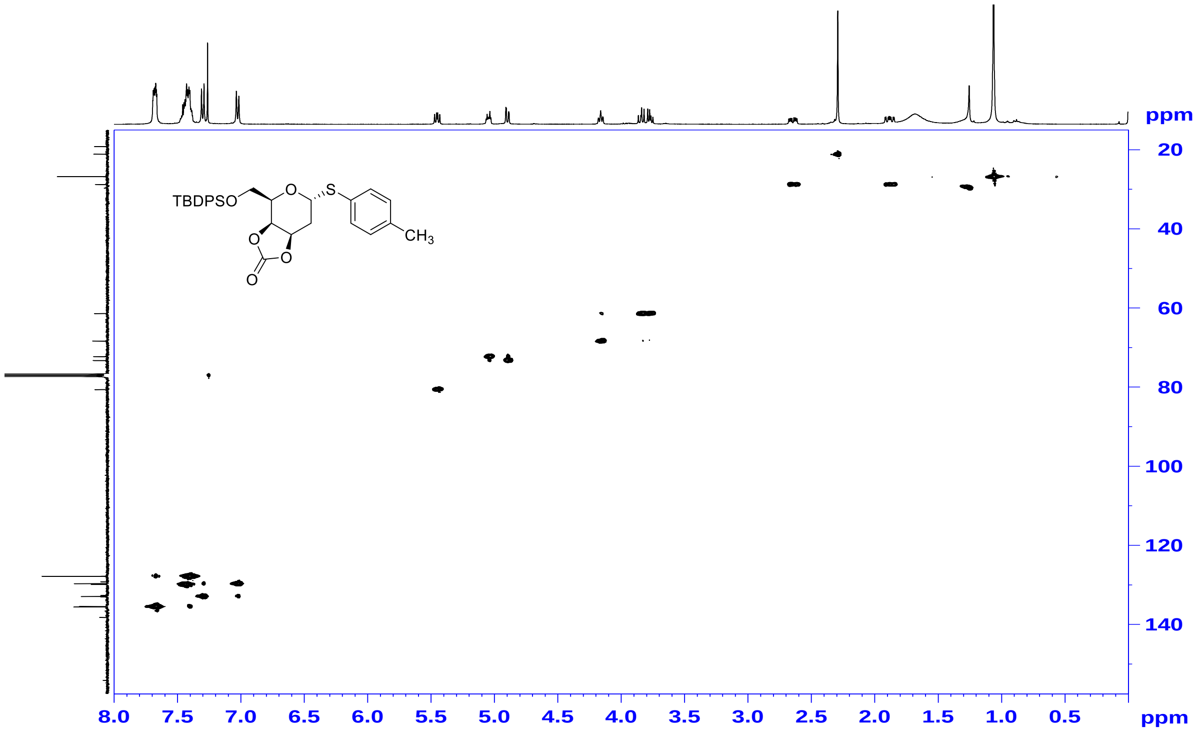

2D NOESY NMR of 3a

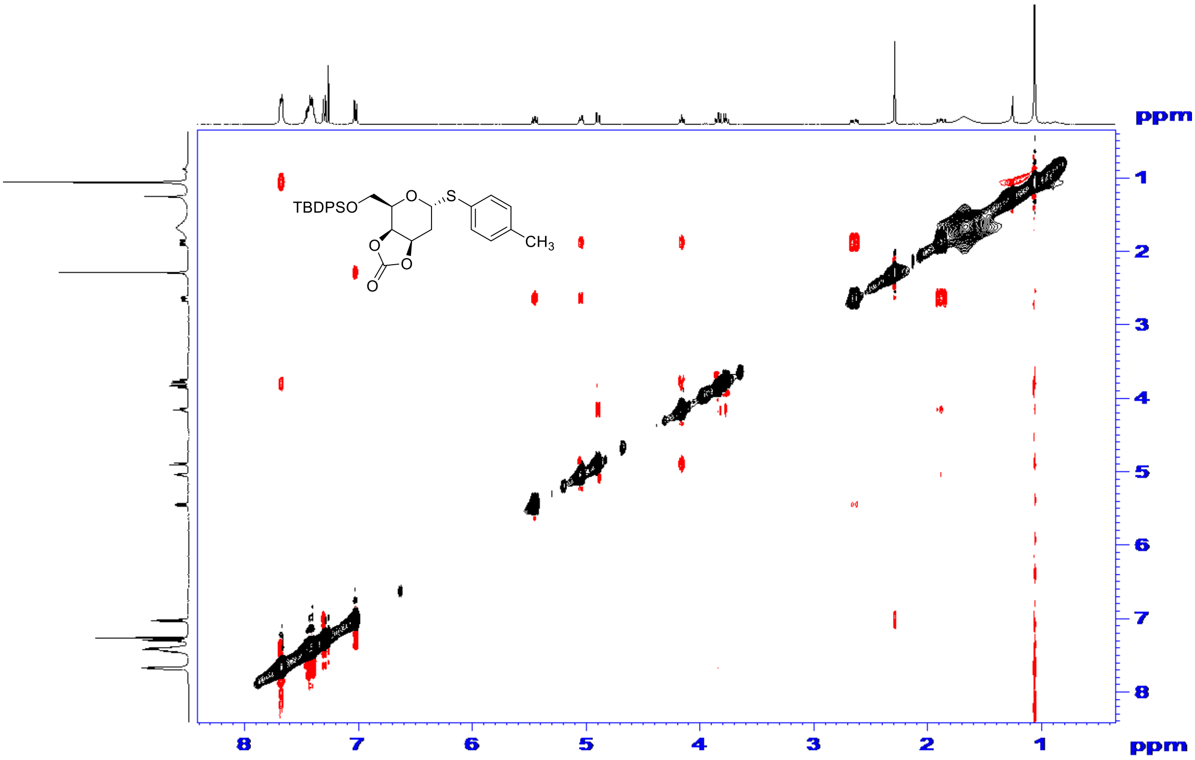

^{1}H NMR of 3b (400 MHz, $CDCl_3$)

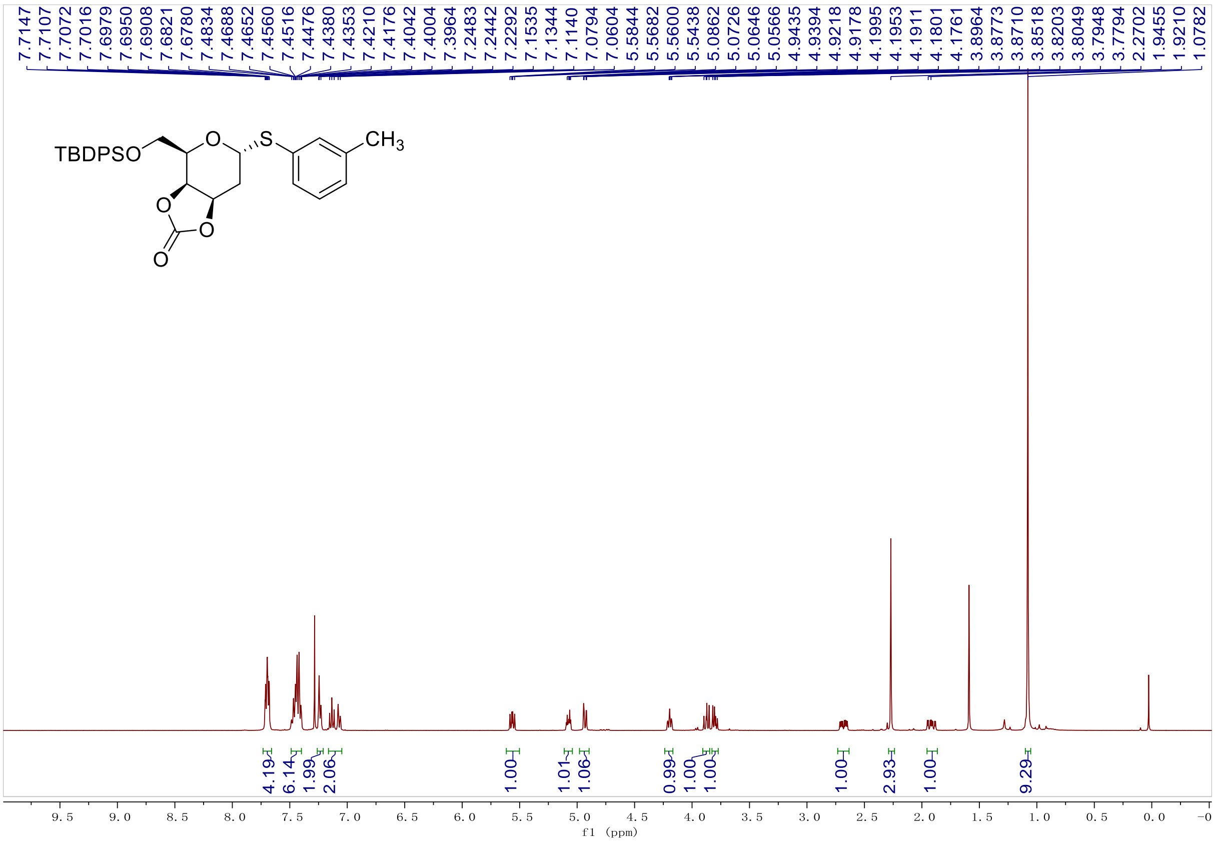

^{13}C NMR of 3b (100 MHz, $CDCl_3$)

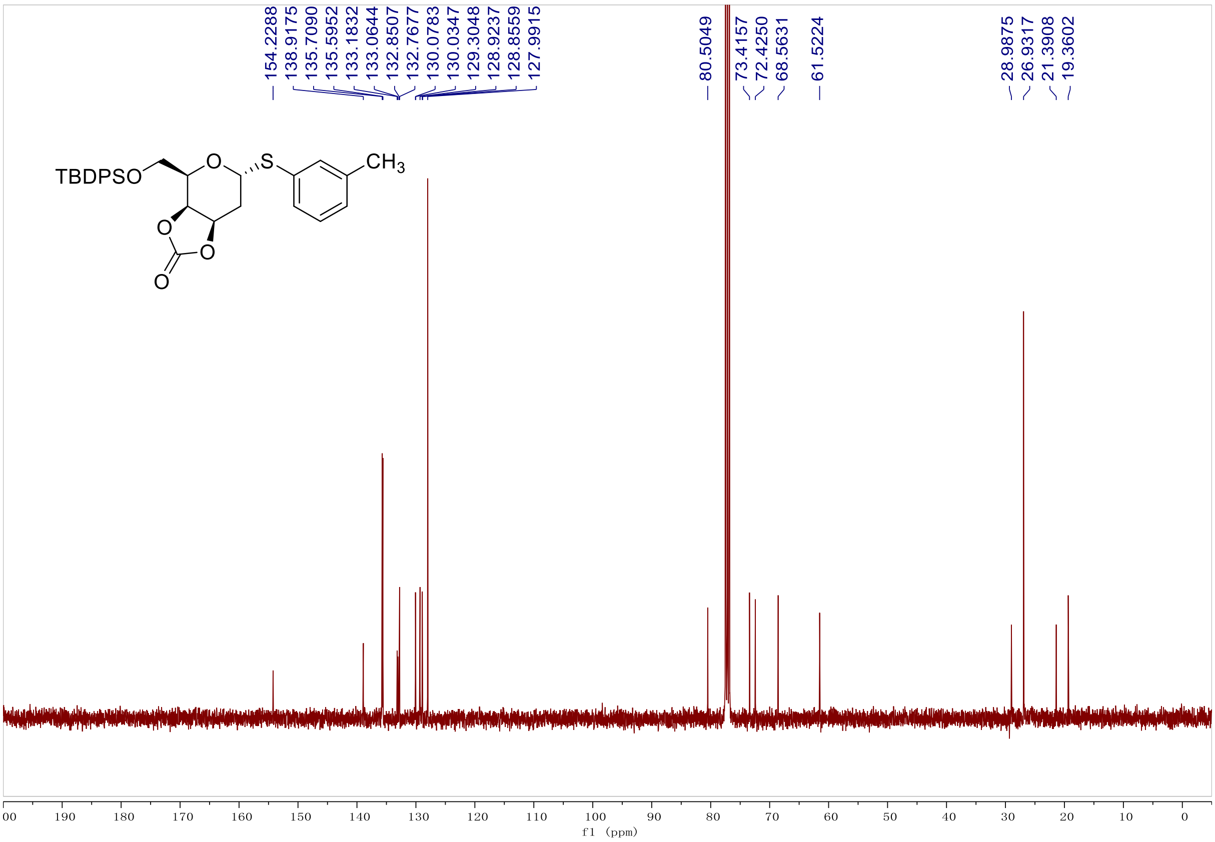

^{1}H NMR of 3c (400 MHz, $CDCl_3$)

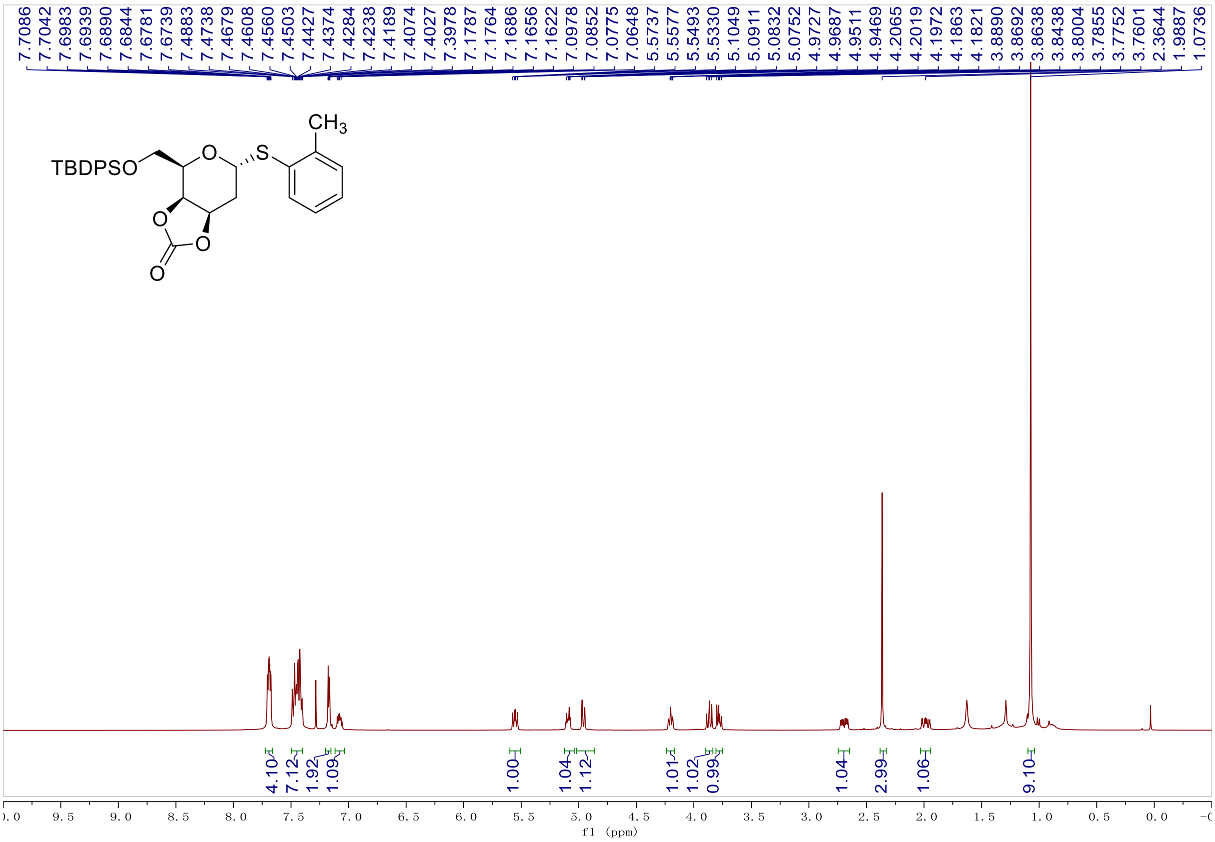

^{13}C NMR of 3c (100 MHz, $CDCl_3$)

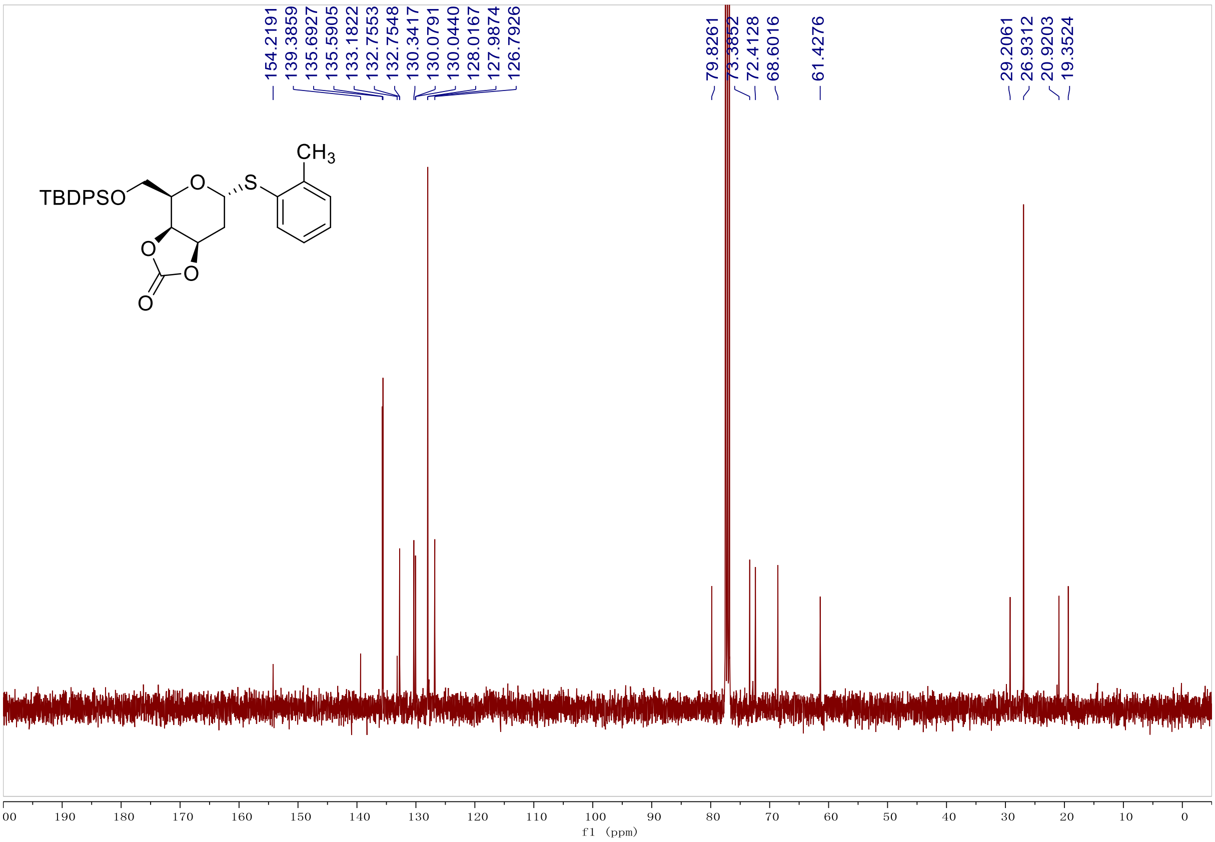

^{1}H NMR of 3d (400 MHz, $CDCl_3$)

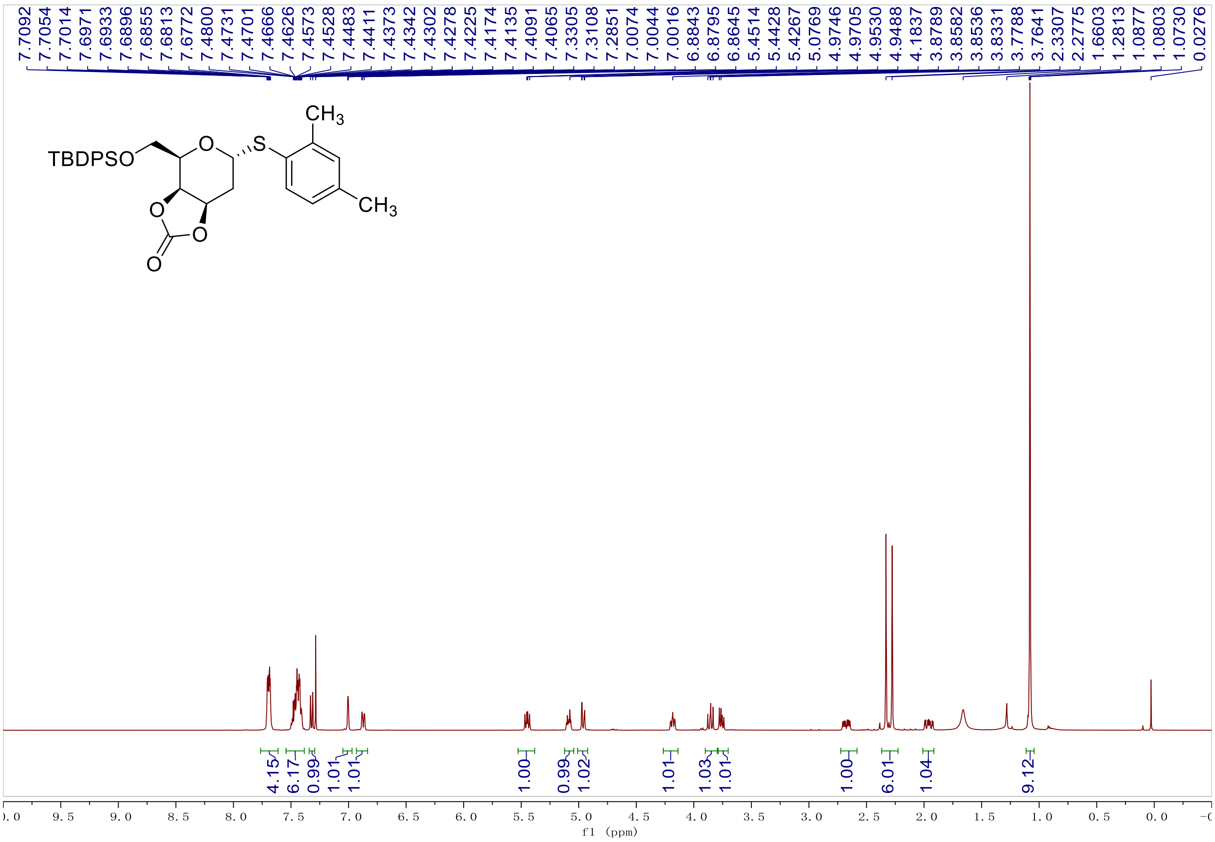

^{13}C NMR of 3d (100 MHz, $CDCl_3$)

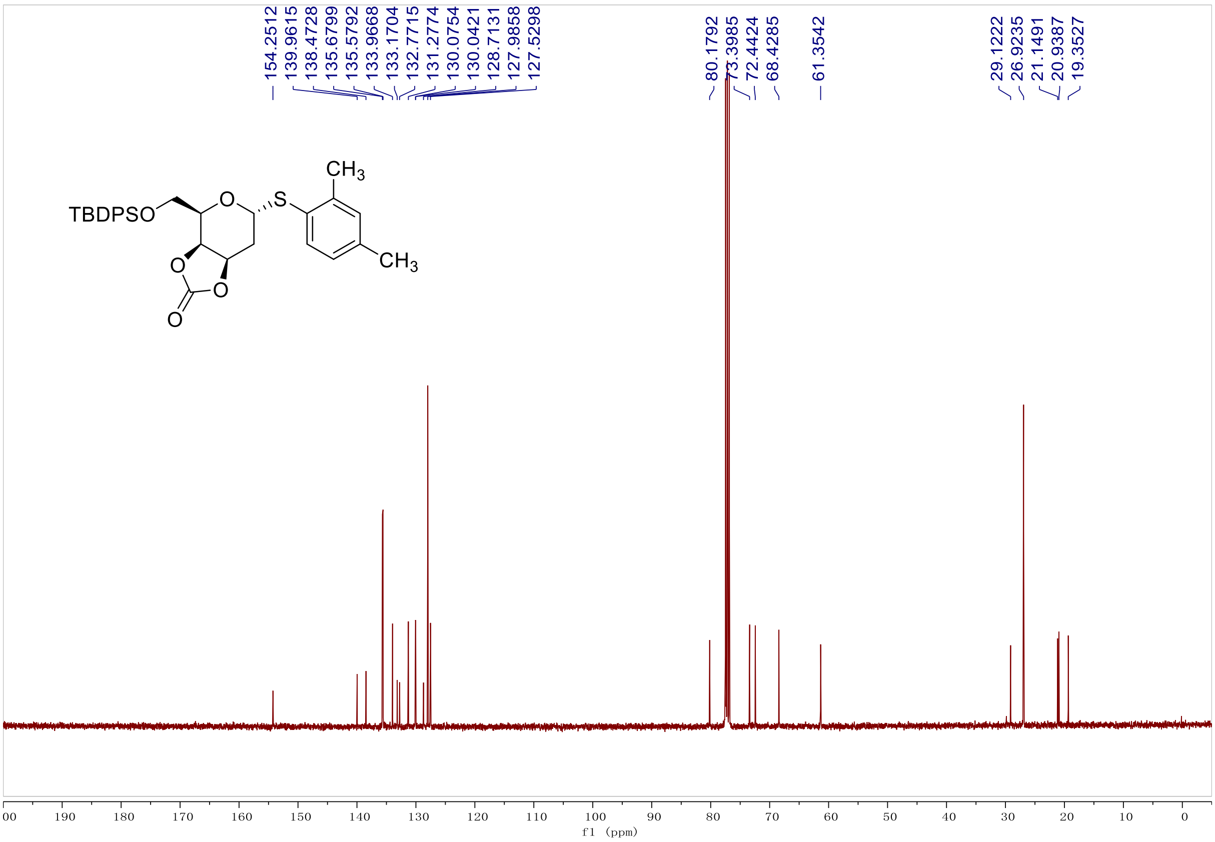

^{1}H NMR of 3e (400 MHz, $CDCl_3$)

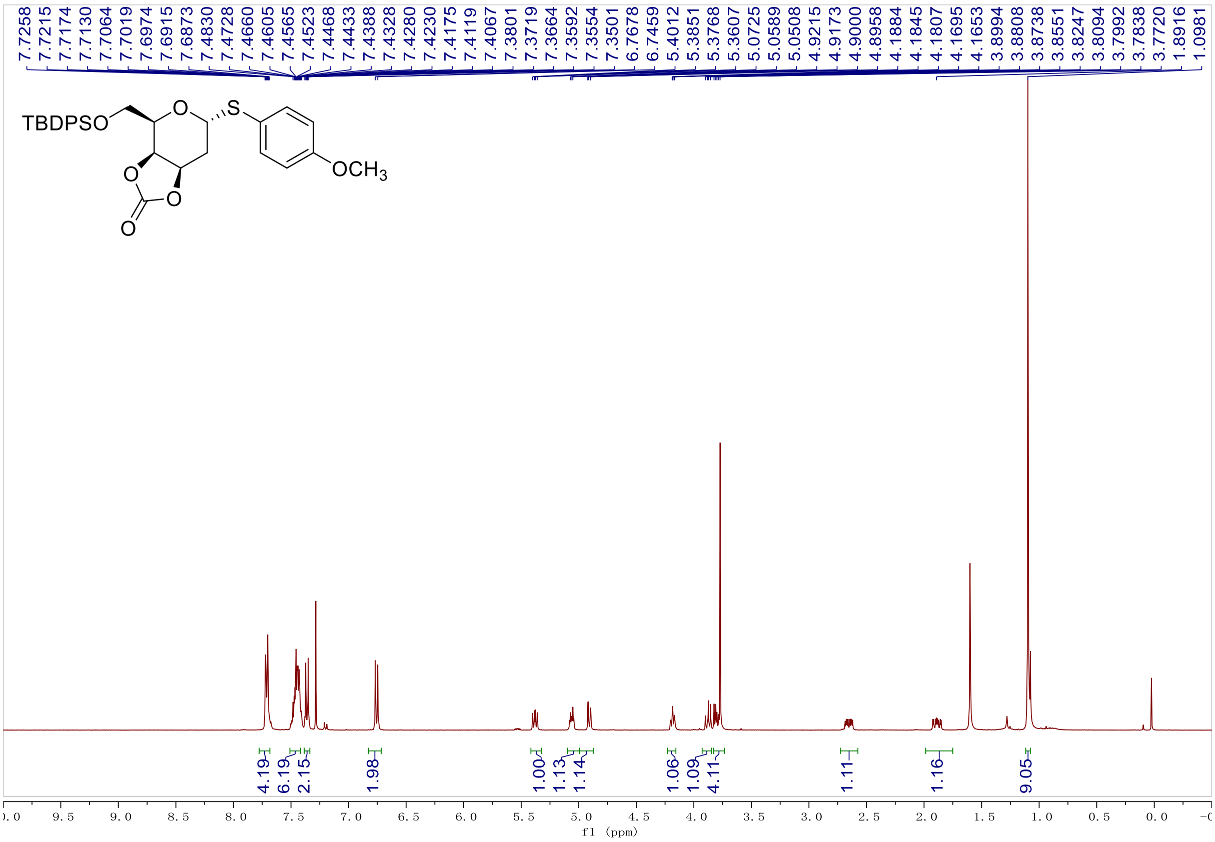

^{13}C NMR of 3e (100 MHz, $CDCl_3$)

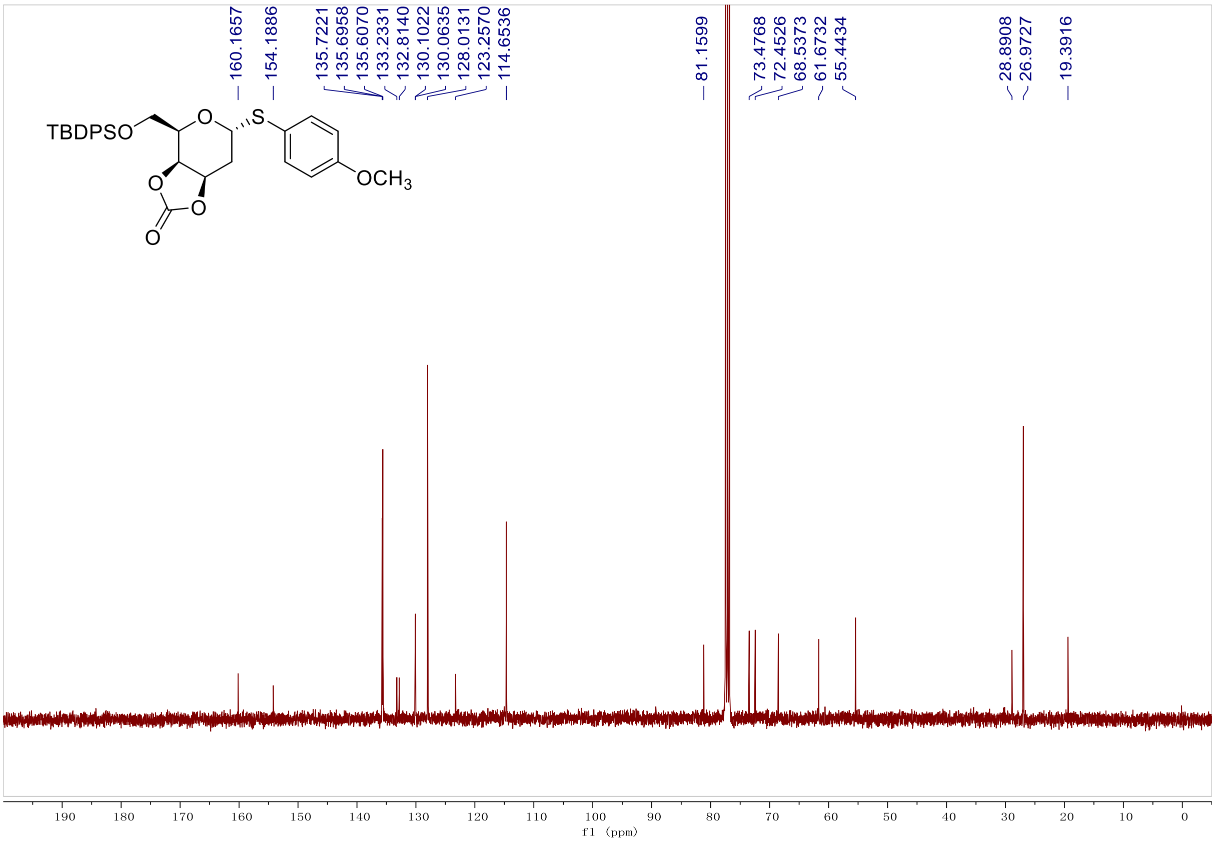

^{1}H NMR of 3f (400 MHz, $CDCl_3$)

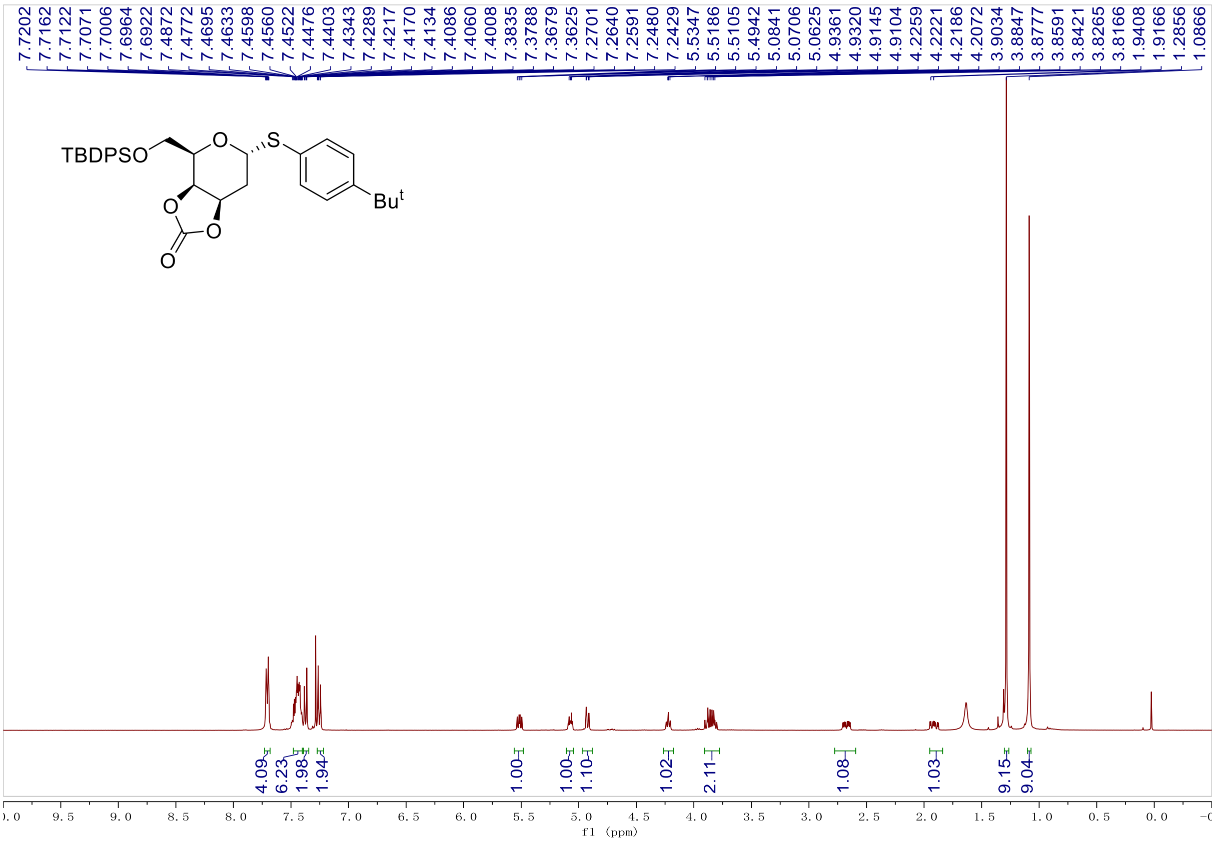

^{13}C NMR of 3f (100 MHz, $CDCl_3$)

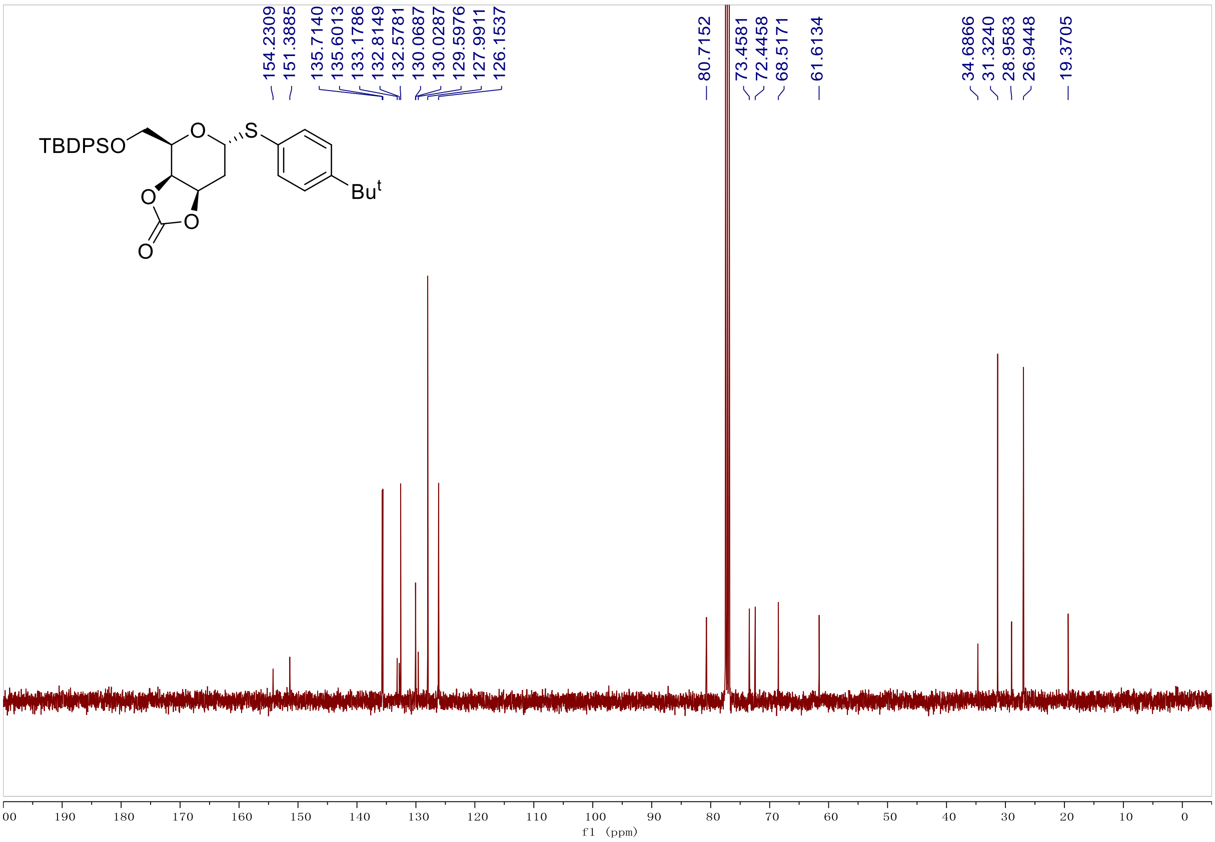

^{1}H NMR of 3g (400 MHz, $CDCl_3$)

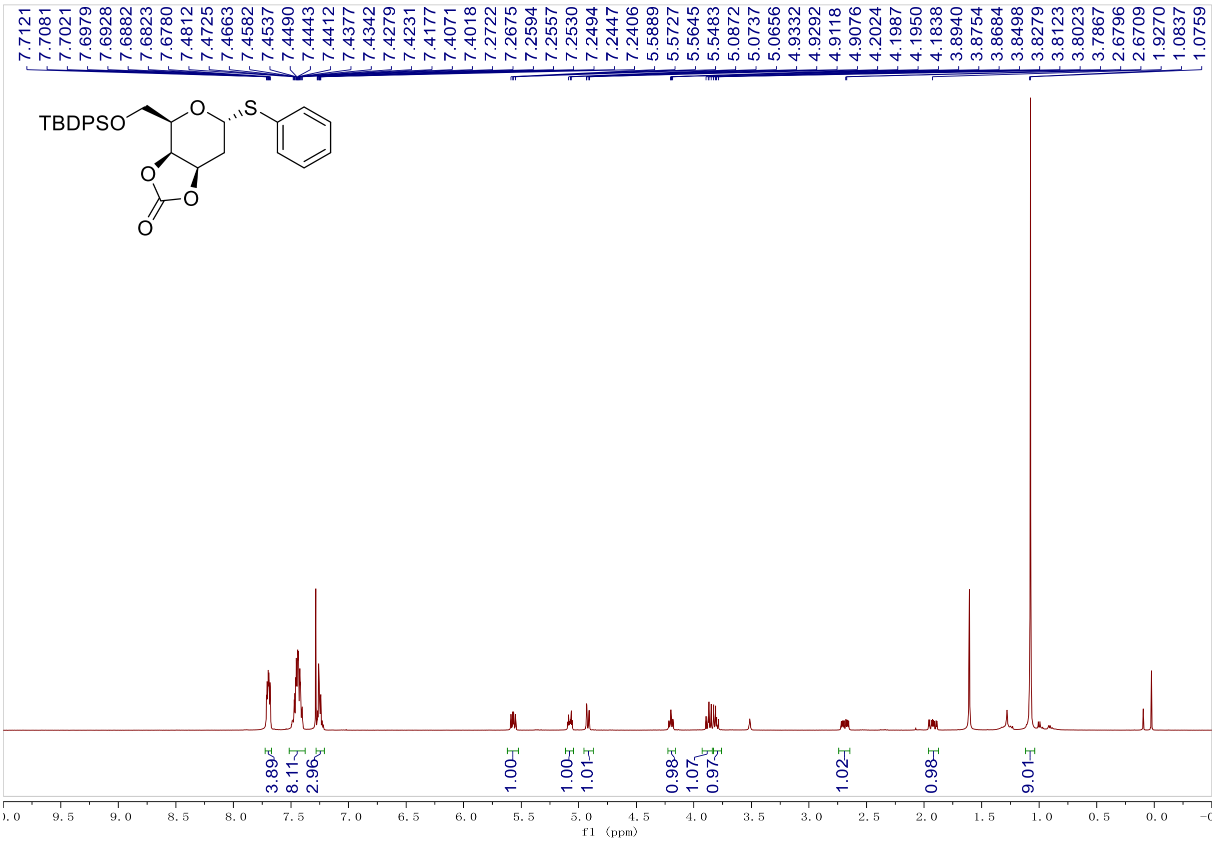

^{13}C NMR of 3g (100 MHz, CDCl$_3$)

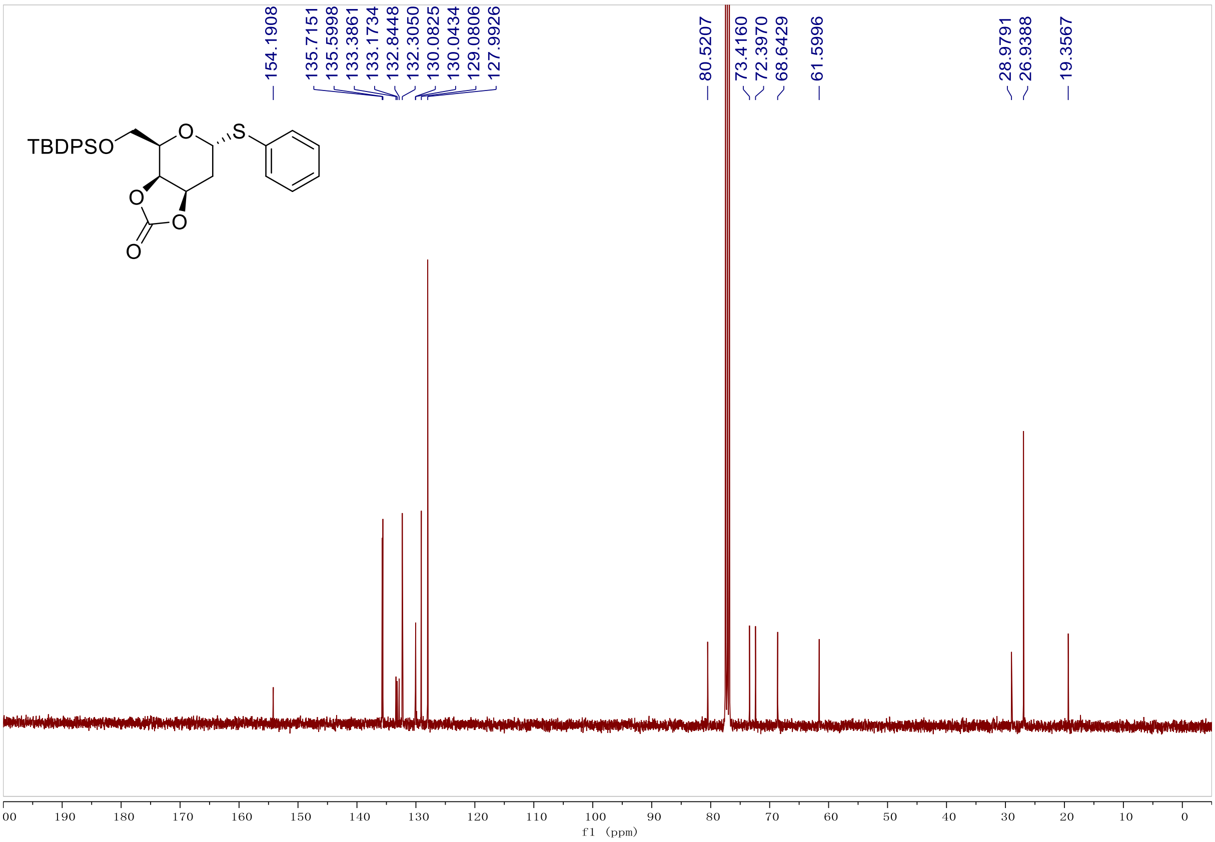

^{1}H NMR of 3h (400 MHz, CDCl$_3$)

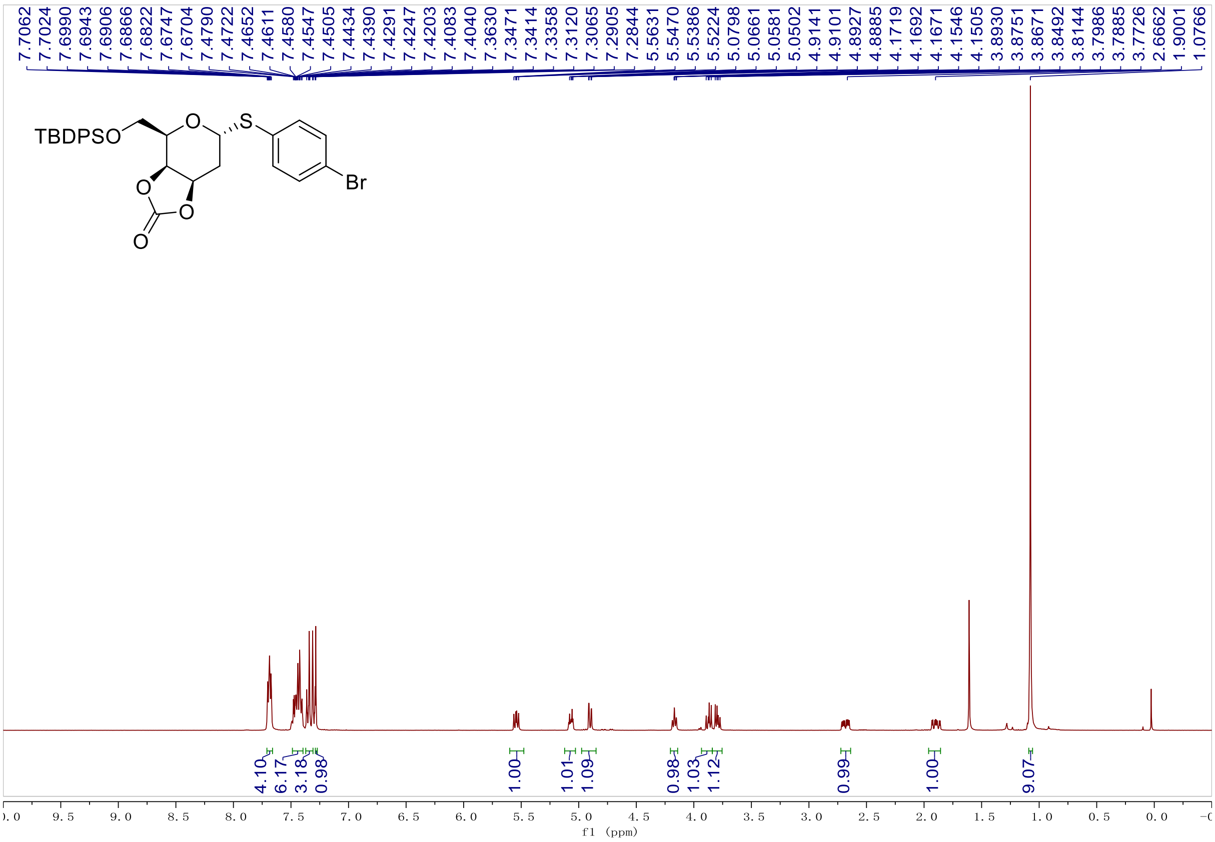

^{13}C NMR of 3h (100 MHz, $CDCl_3$)

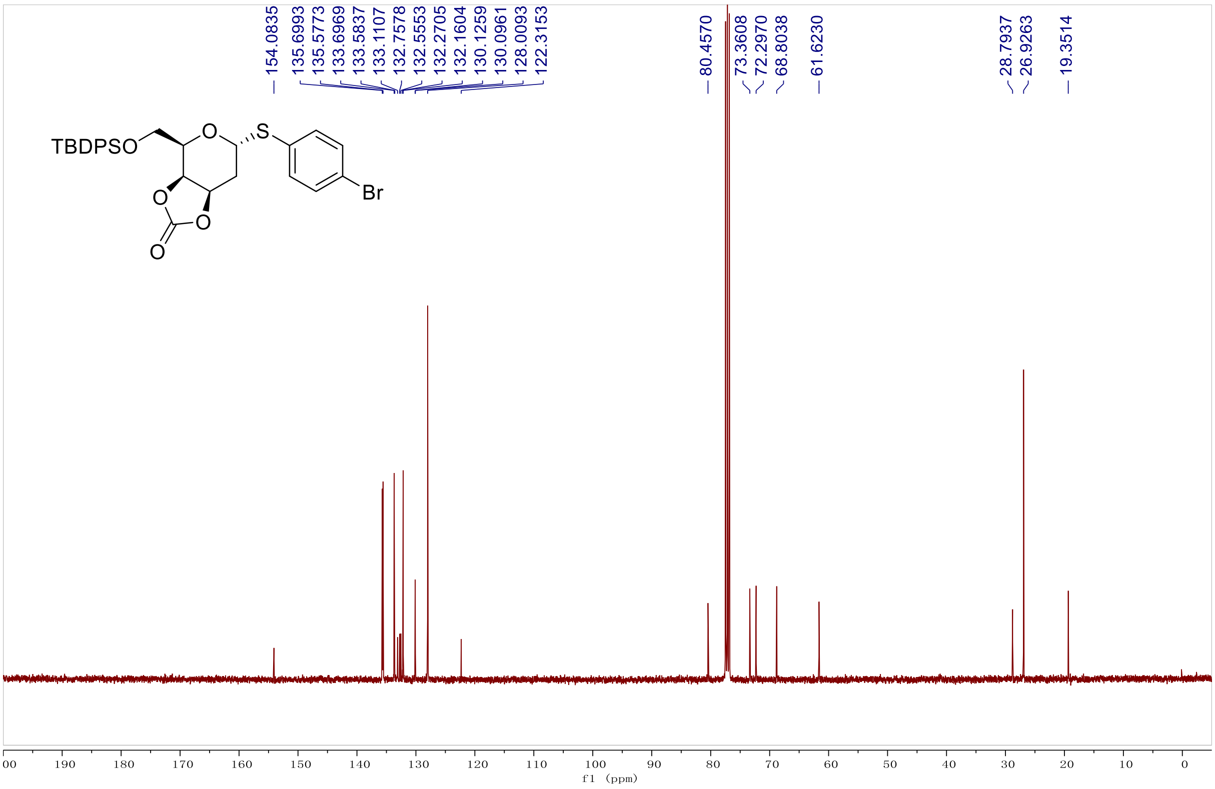

^{1}H NMR of 3i (400 MHz, $CDCl_3$)

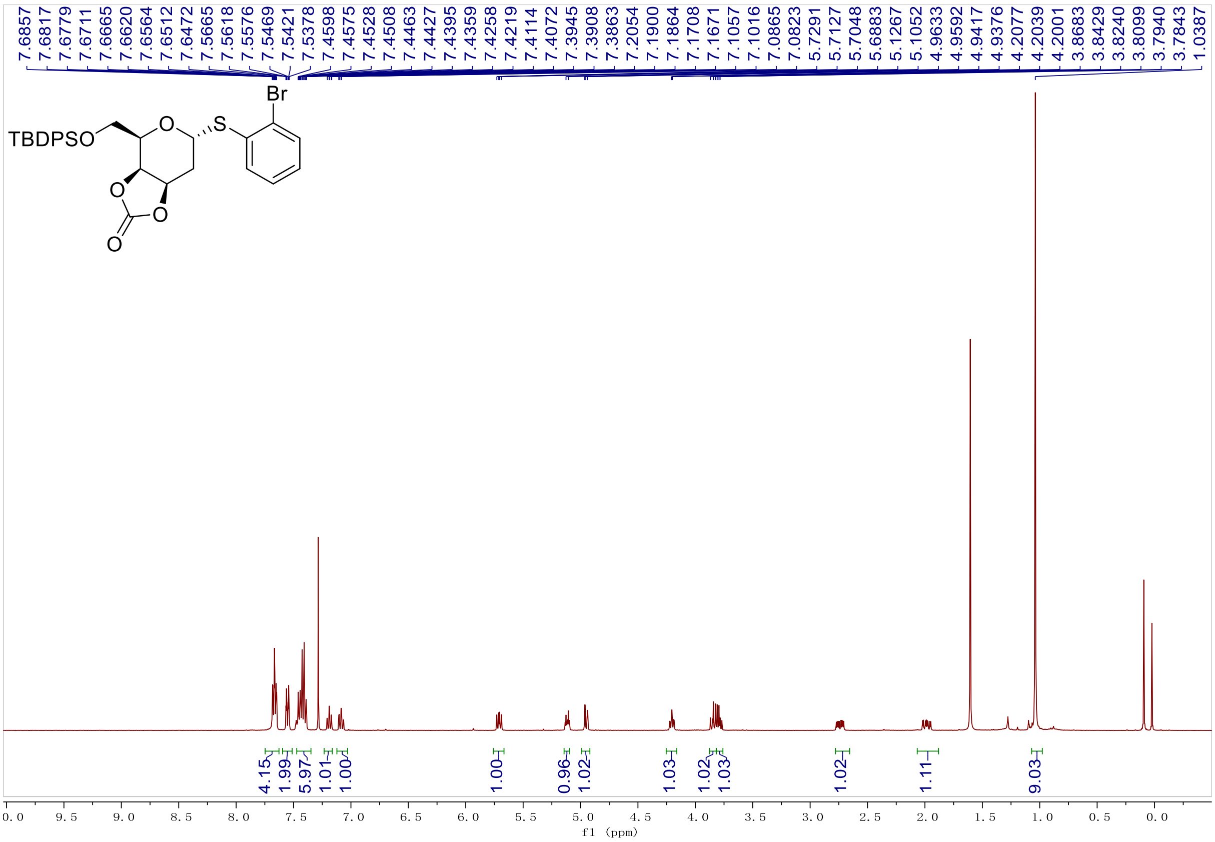

^{13}C NMR of 3i (100 MHz, $CDCl_3$)

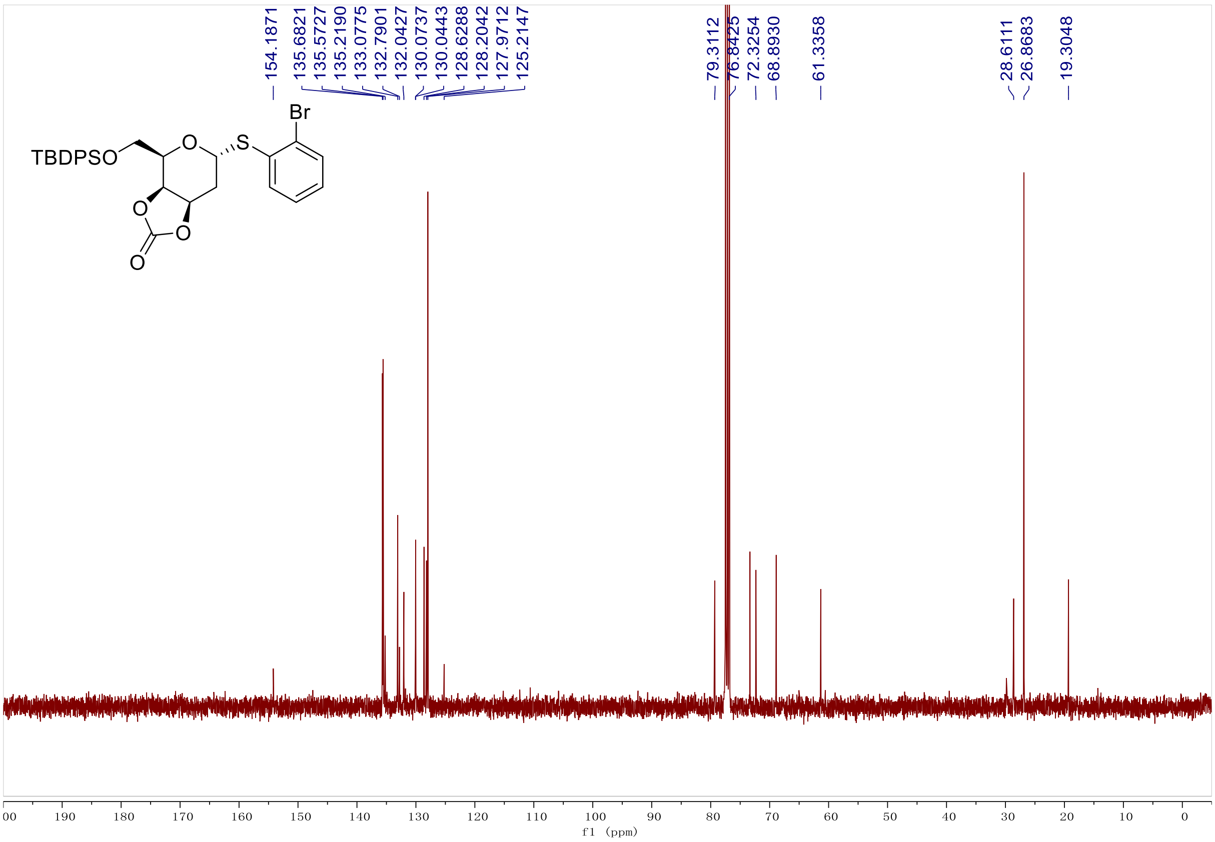

^{1}H NMR of 3j (400 MHz, $CDCl_3$)

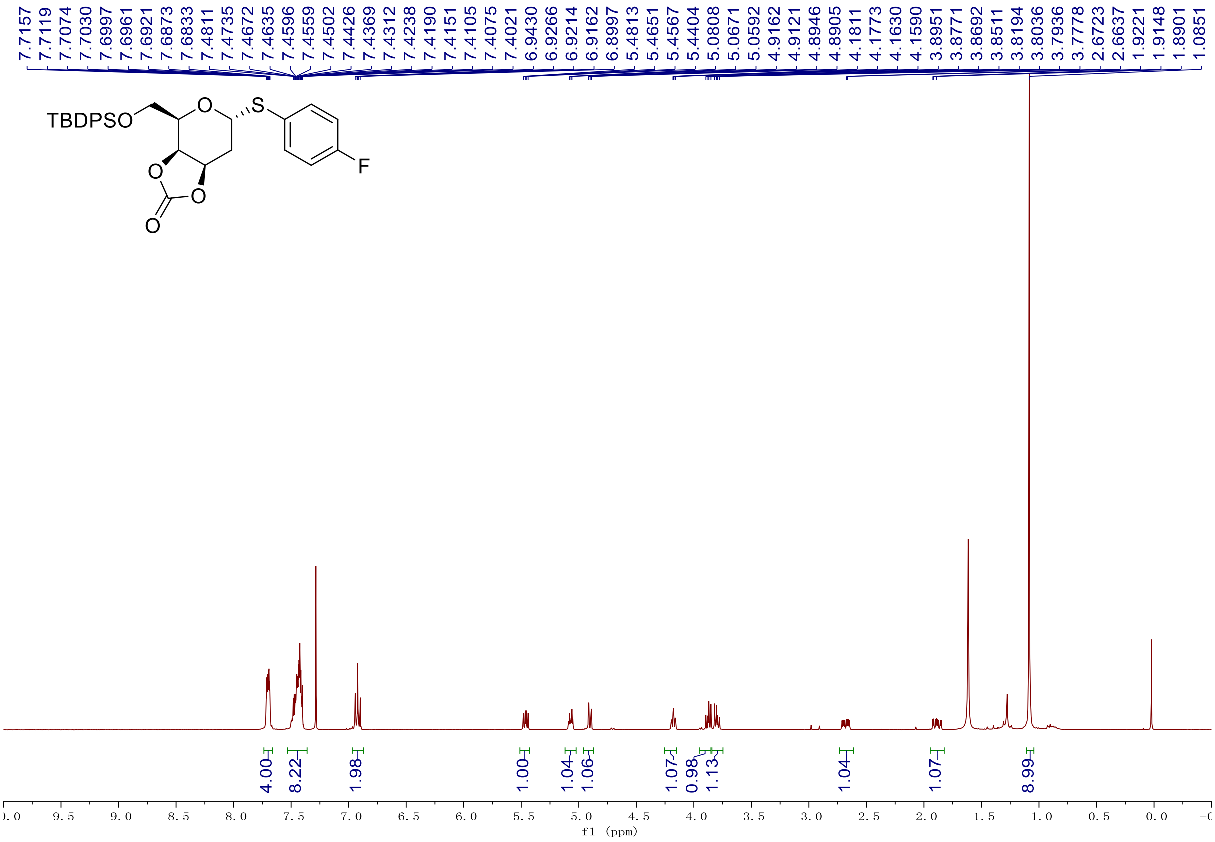

^{13}C NMR of 3j (100 MHz, $CDCl_3$)

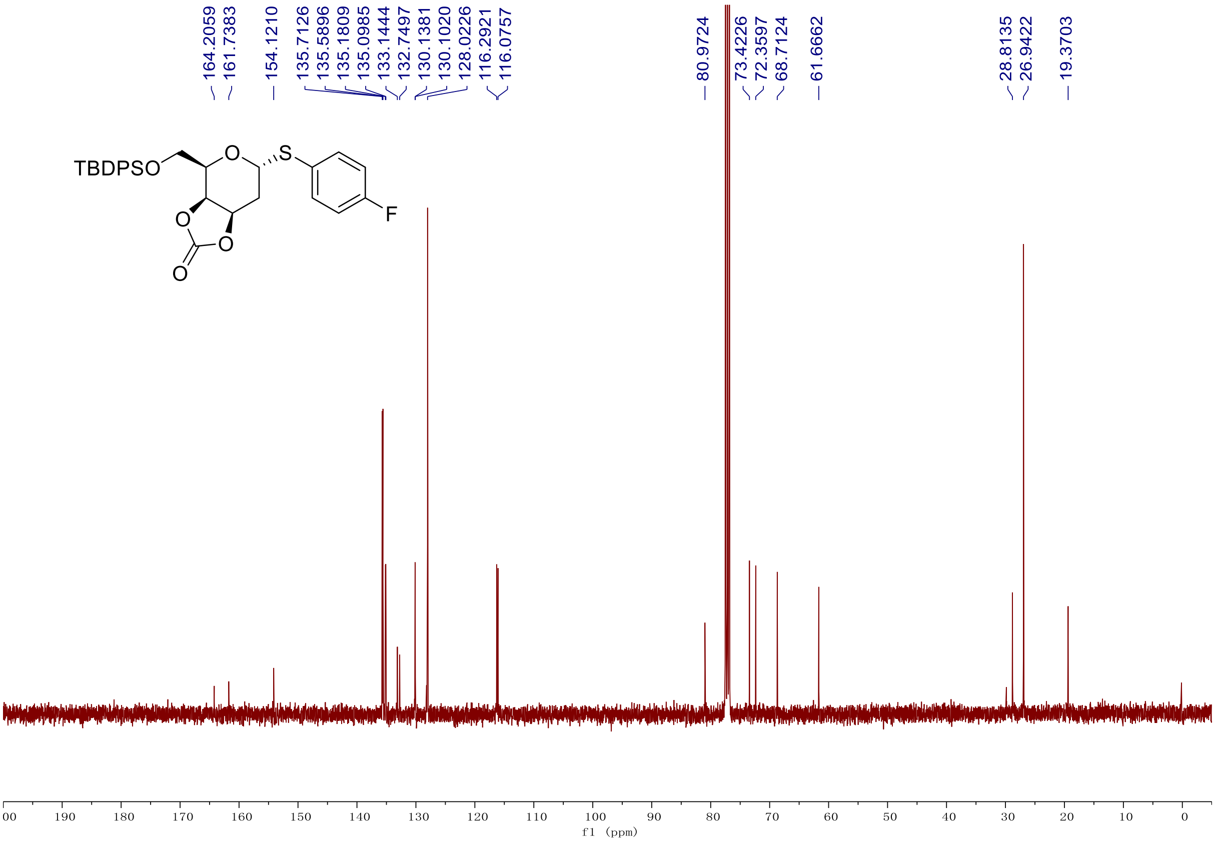

^{1}H NMR of 5a (400 MHz, $CDCl_3$)

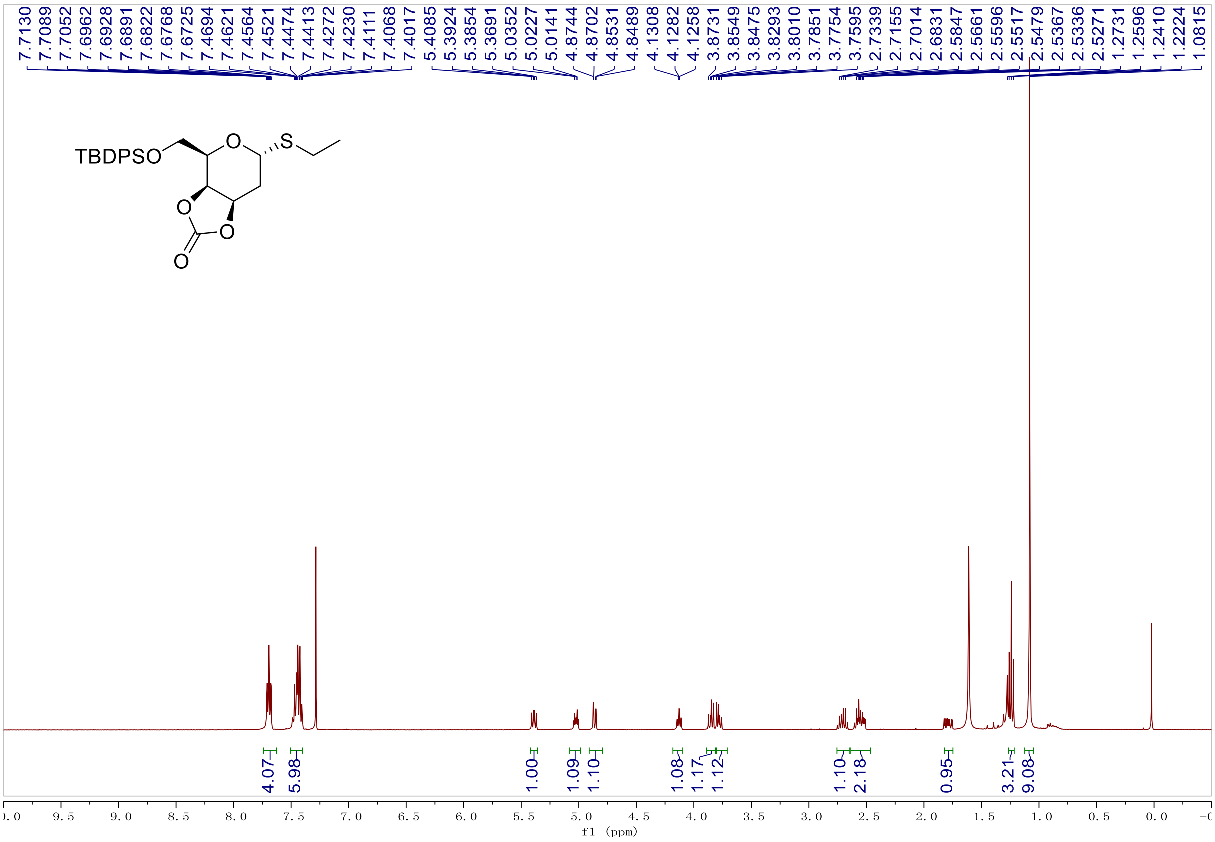

^{13}C NMR of 5a (100 MHz, $CDCl_3$)

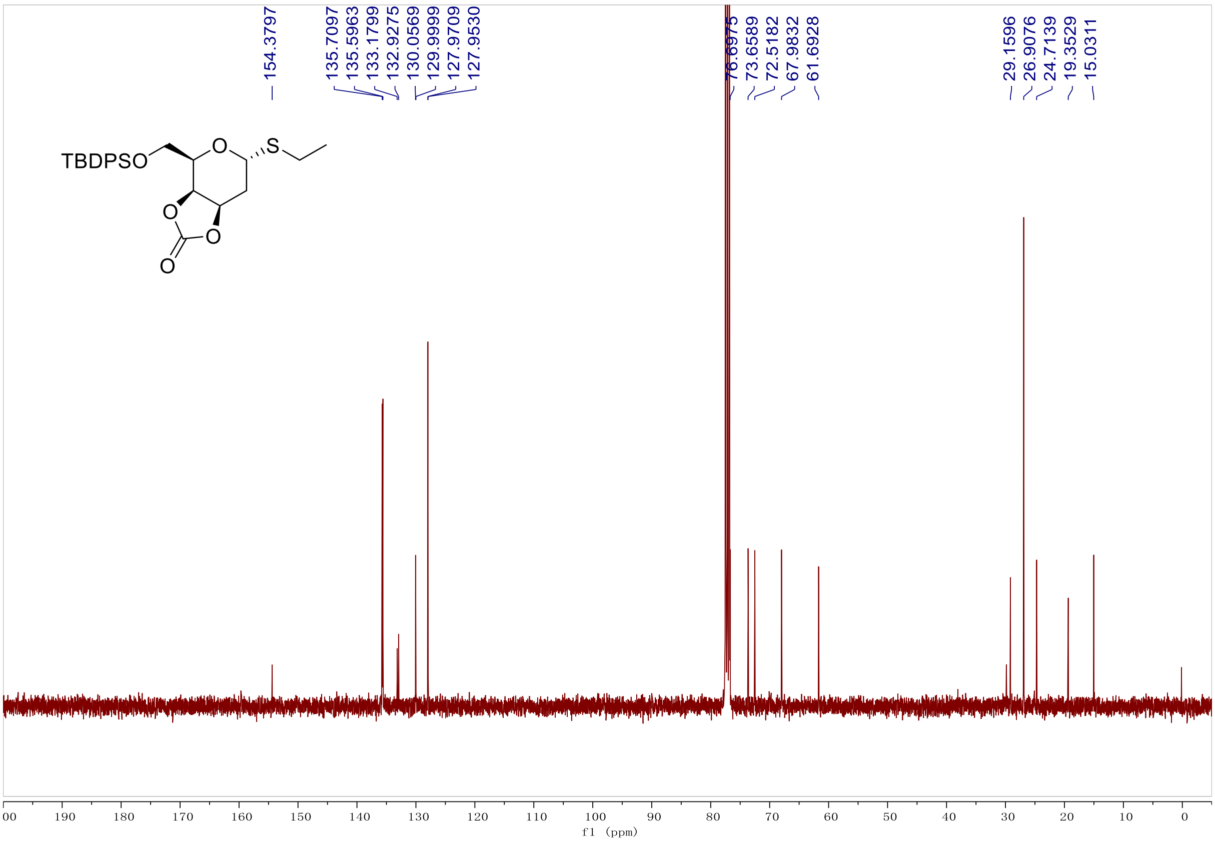

^{1}H NMR of 5b (400 MHz, $CDCl_3$)

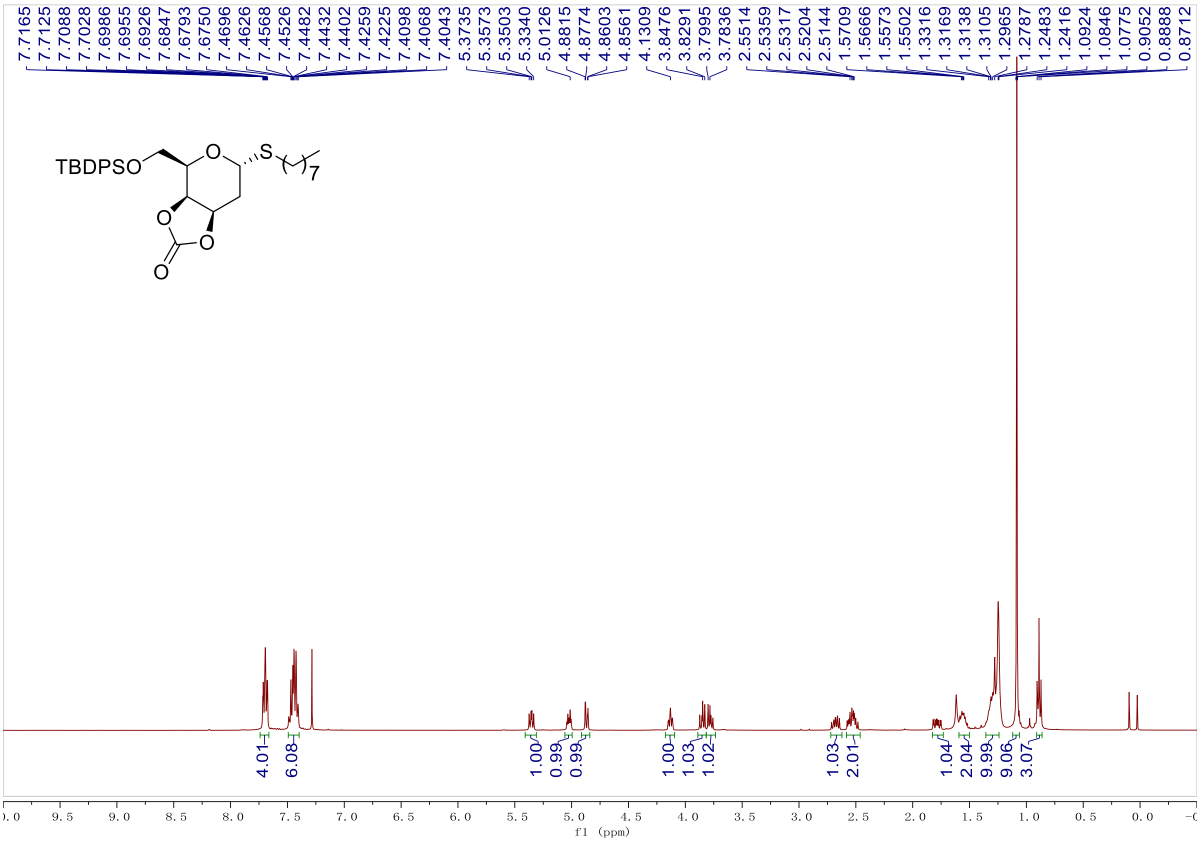

^{13}C NMR of 5b (100 MHz, $CDCl_3$)

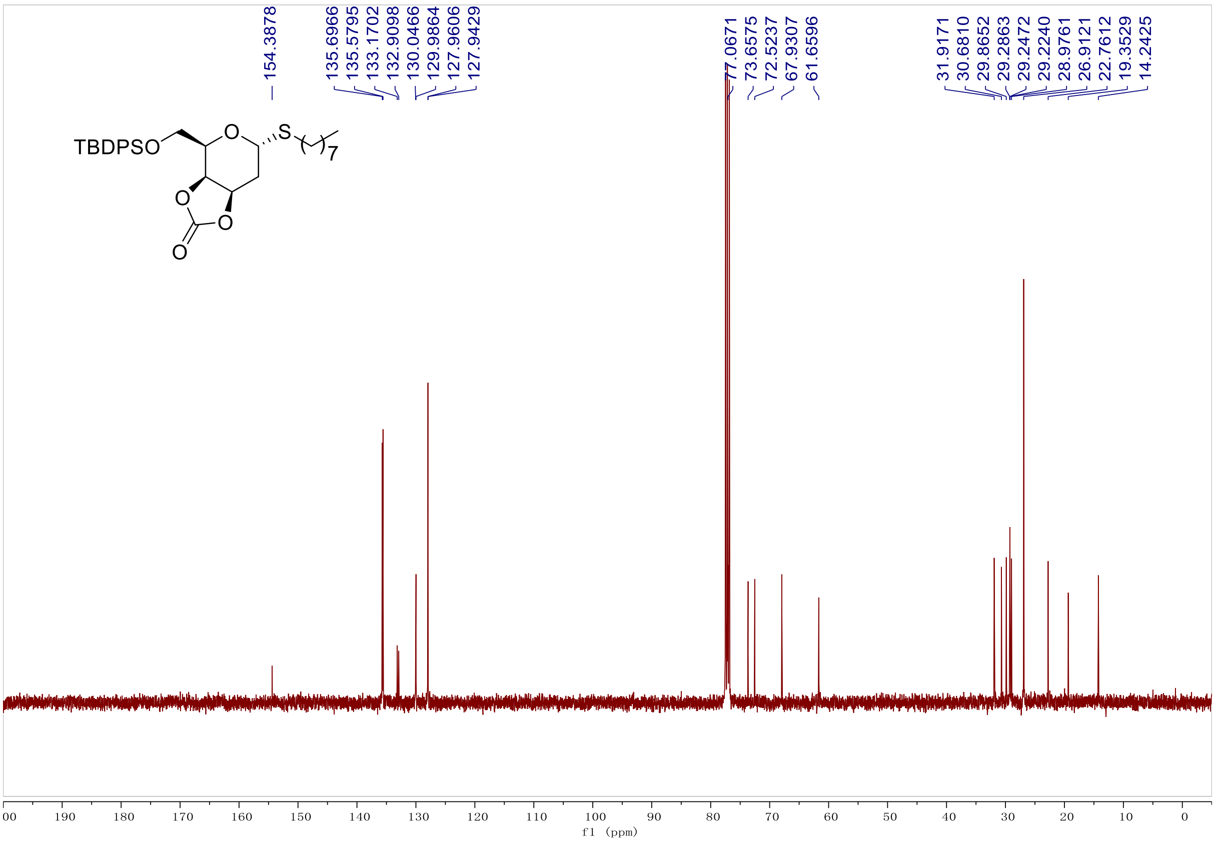

^{1}H NMR of 5c (400 MHz, $CDCl_3$)

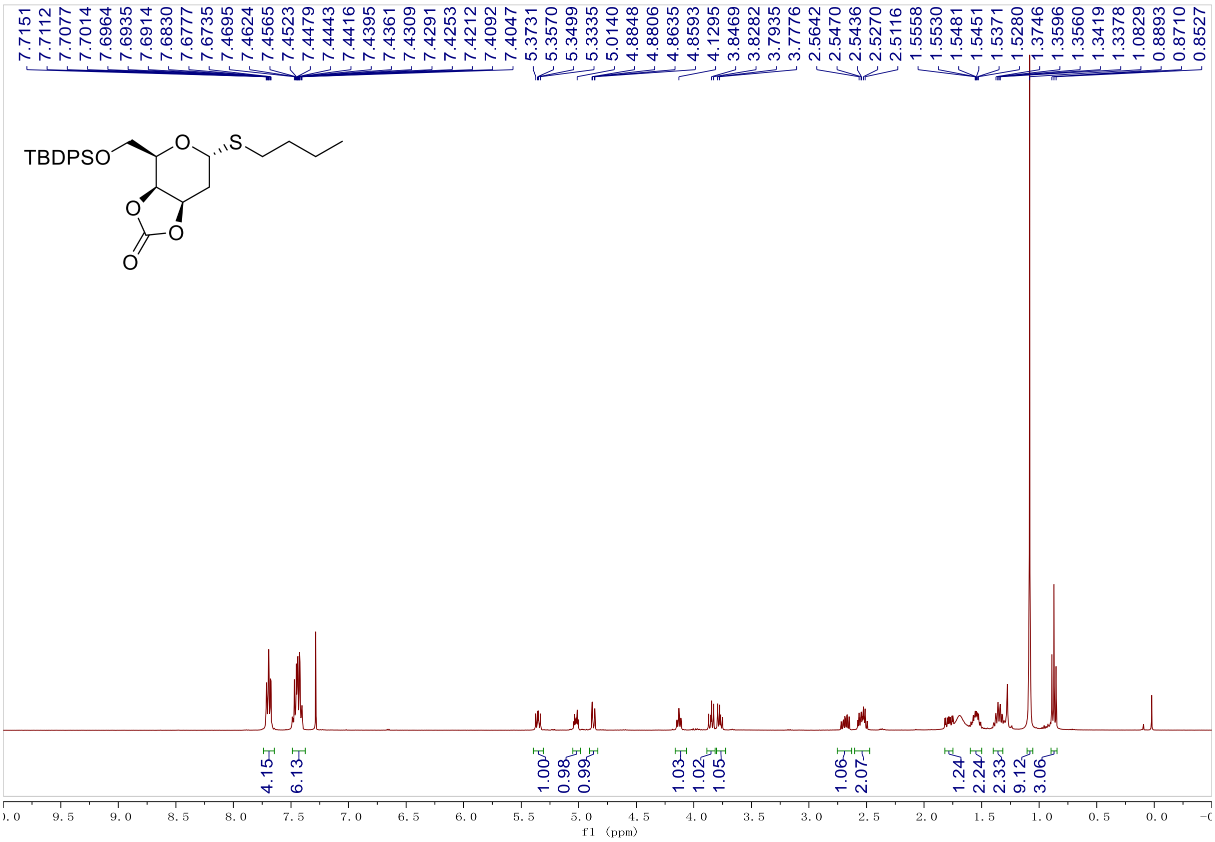

^{13}C NMR of 5c (100 MHz, CDCl$_3$)

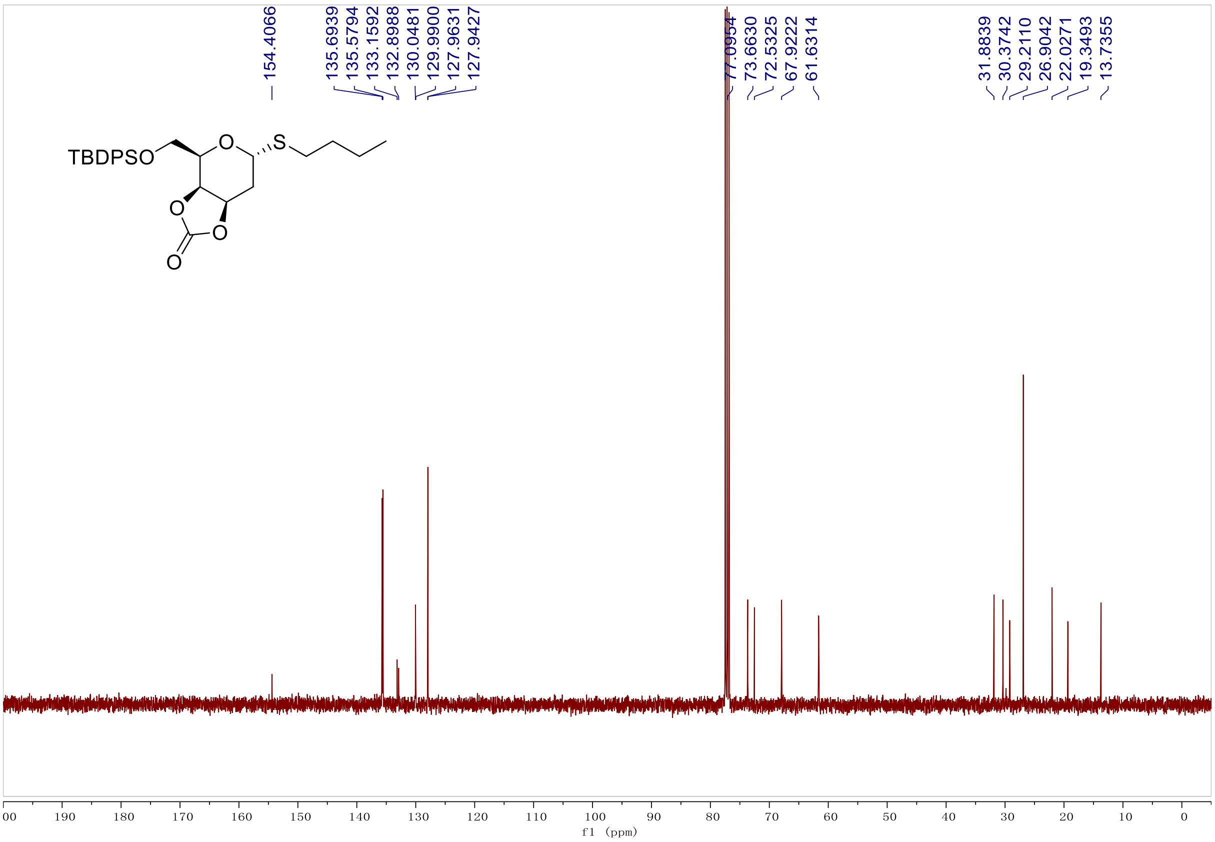

^{1}H NMR of 5d (400 MHz, CDCl$_3$)

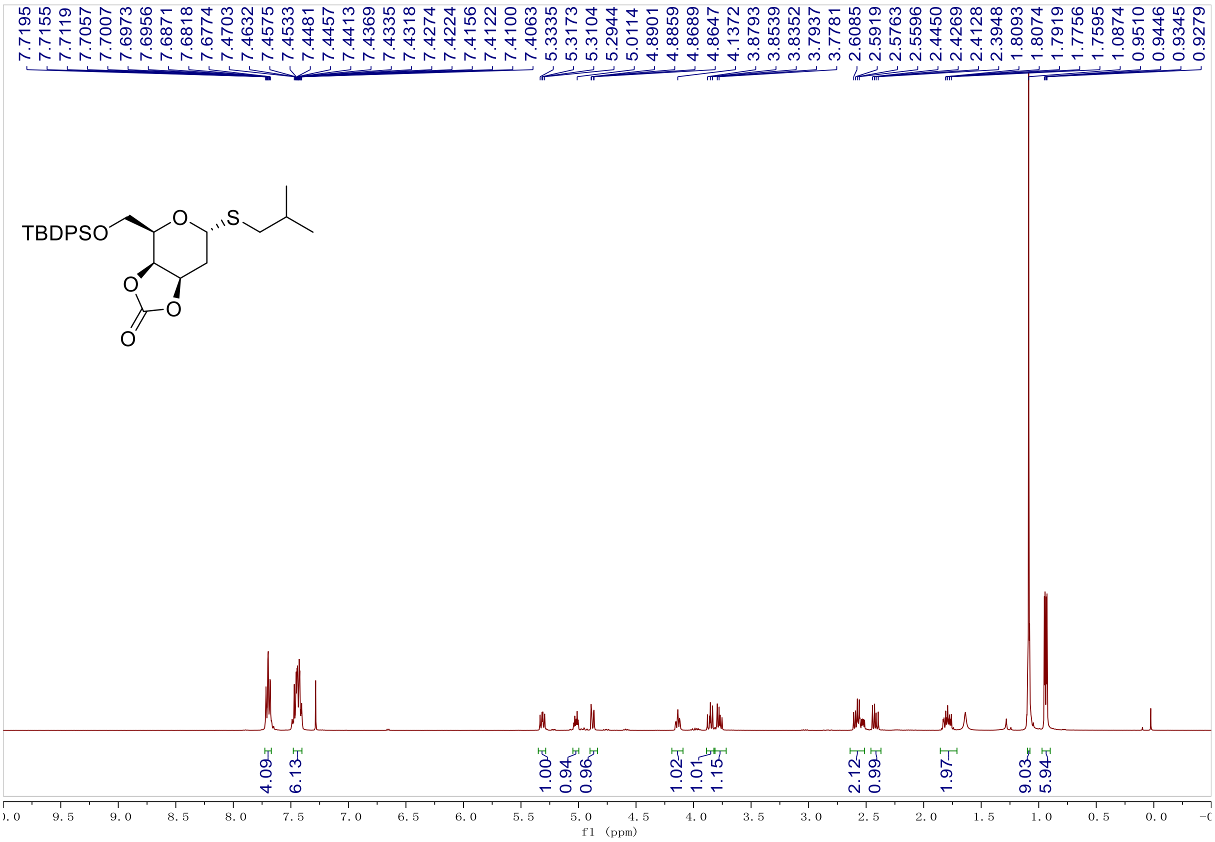

^{13}C NMR of 5d (100 MHz, $CDCl_3$)

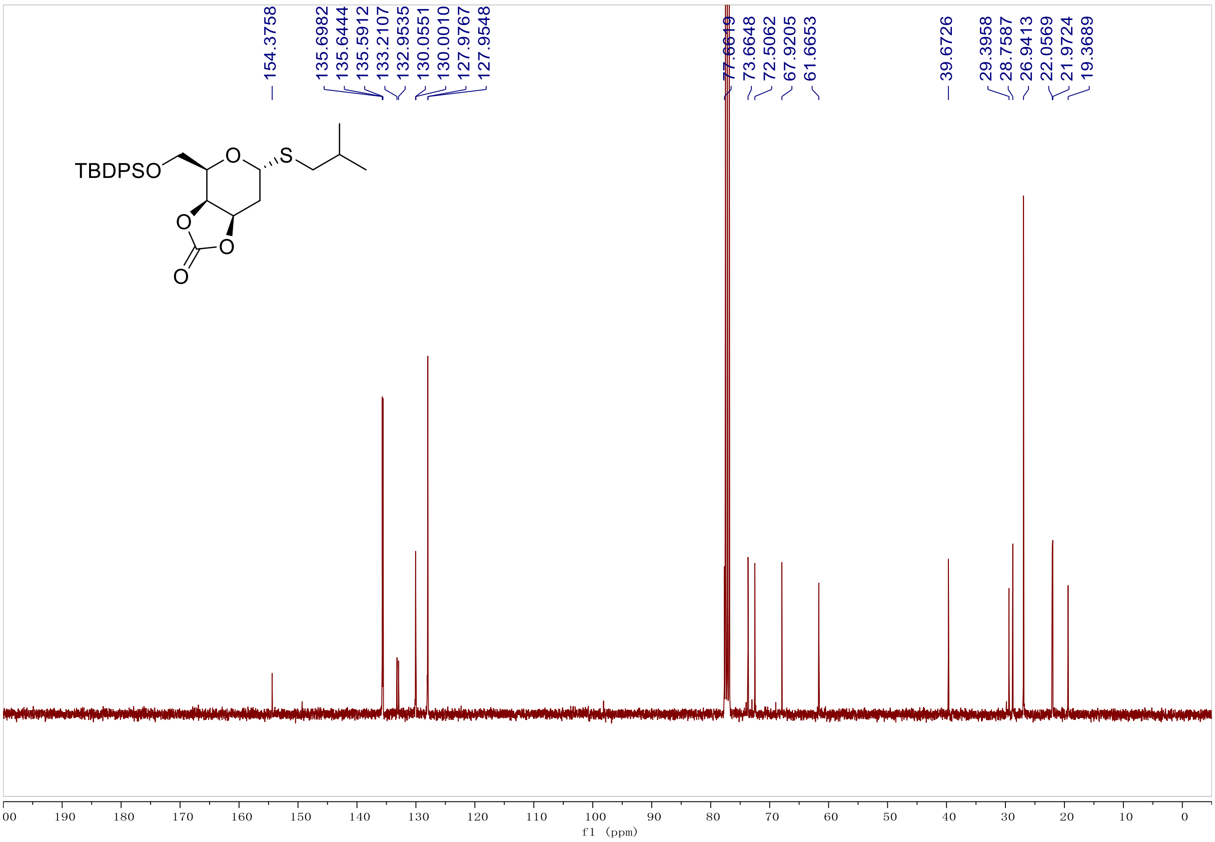

^{1}H NMR of 5e (400 MHz, $CDCl_3$)

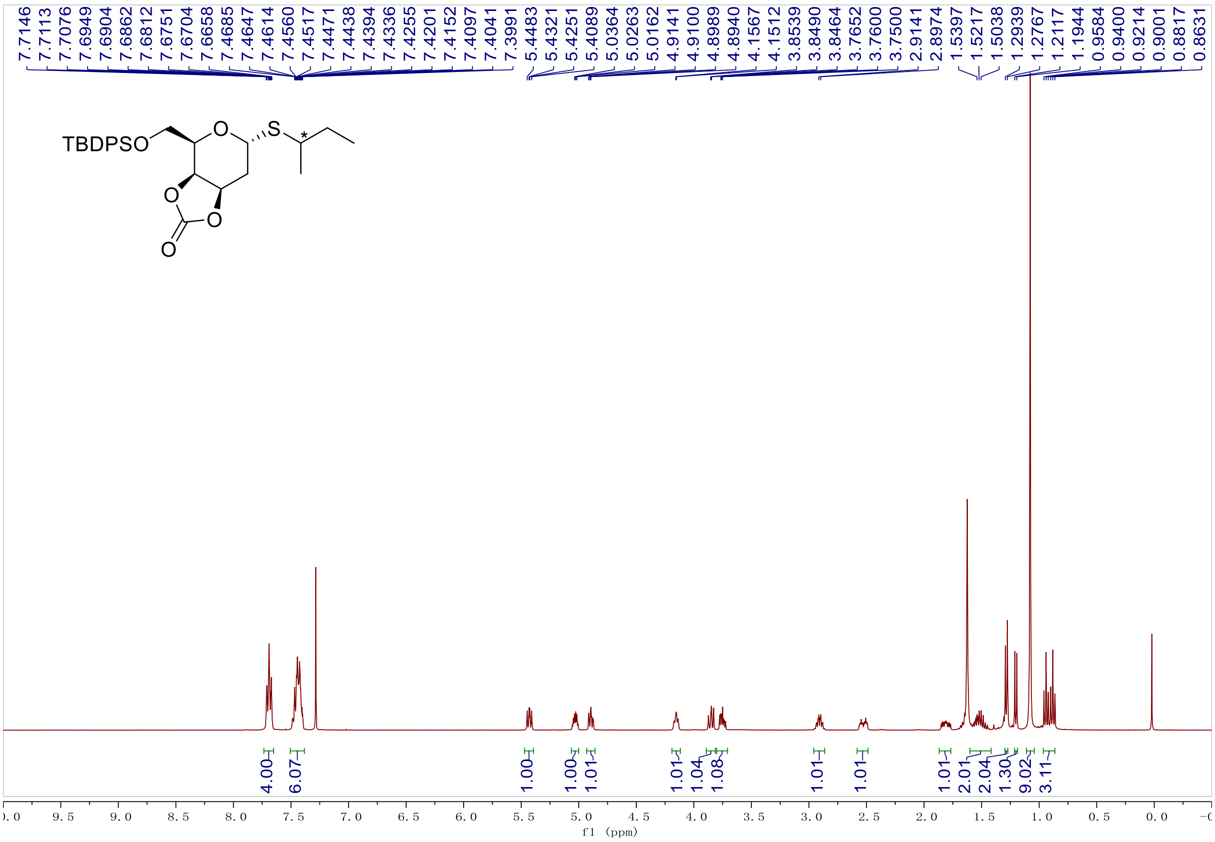

^{13}C NMR of 5e (100 MHz, $CDCl_3$)

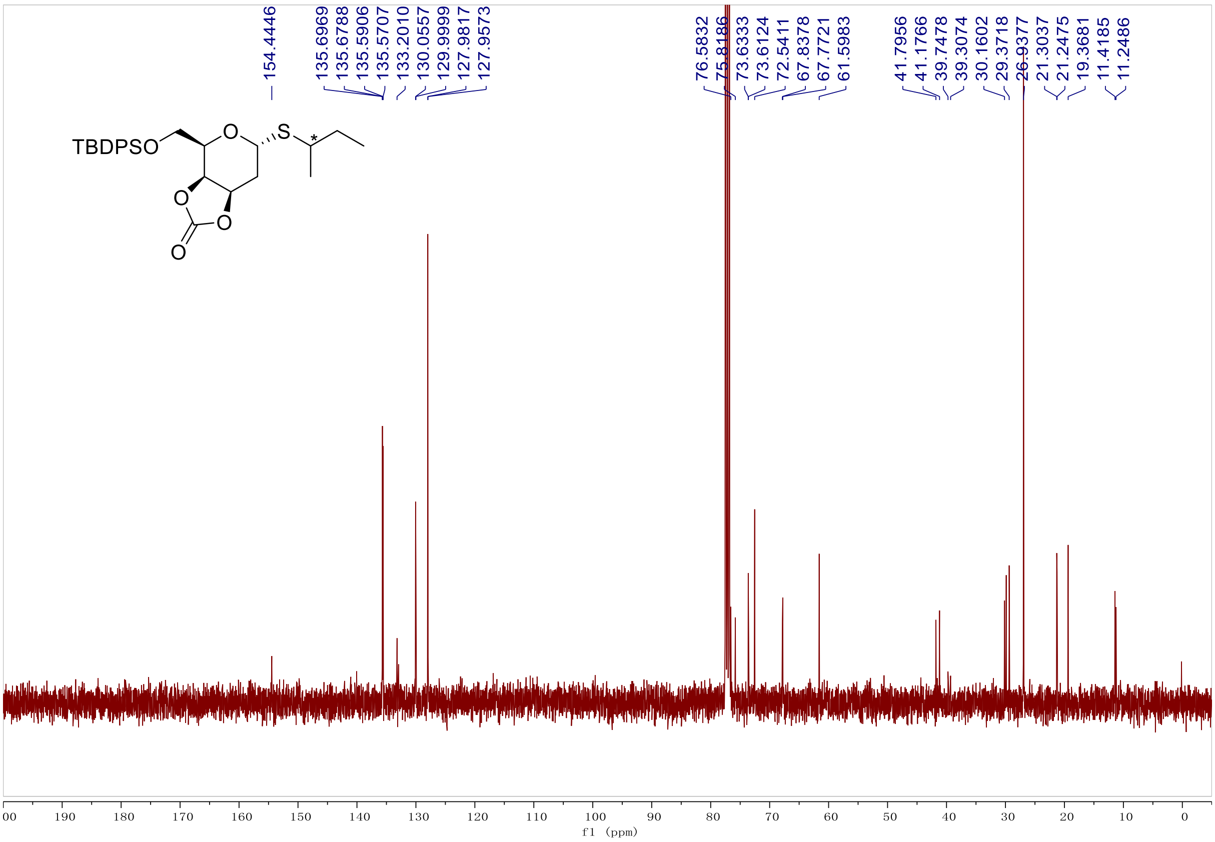

^{1}H NMR of 5f (400 MHz, $CDCl_3$)

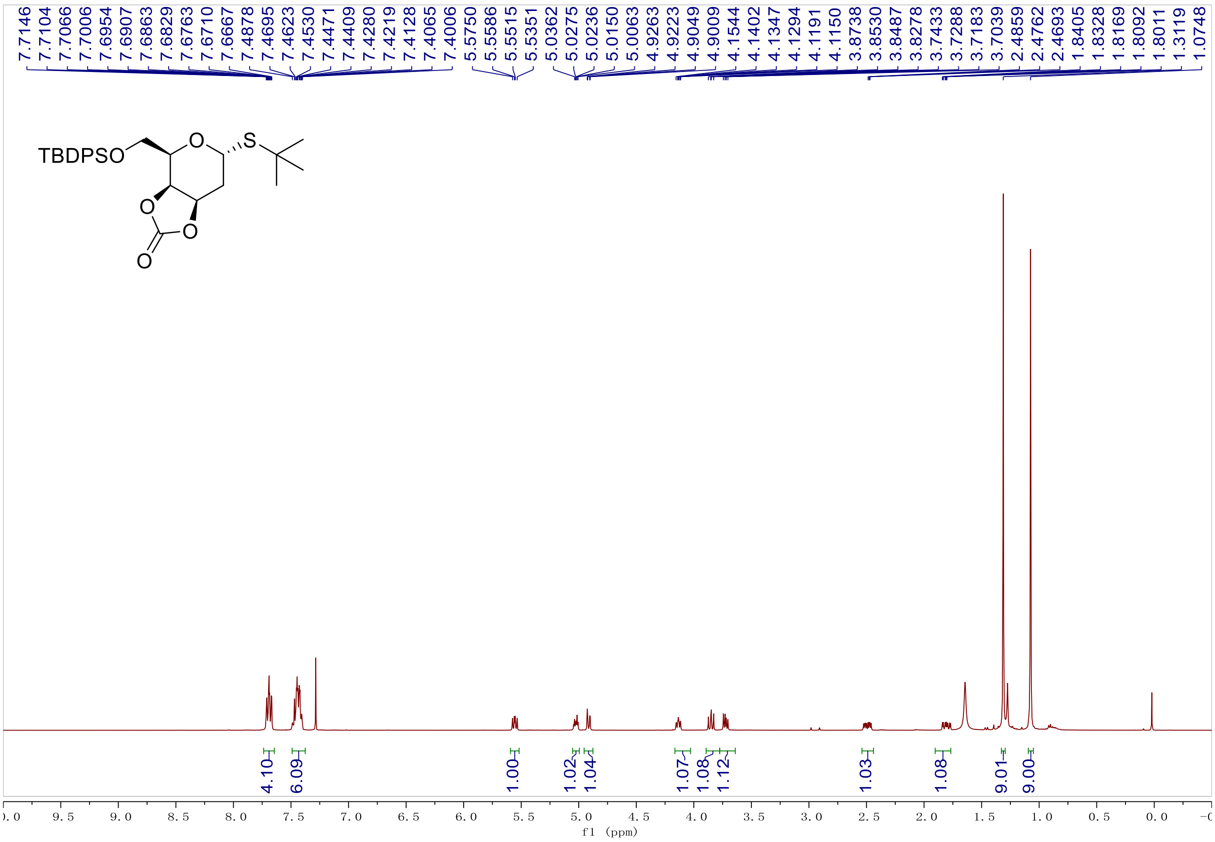

^{13}C NMR of 5f (100 MHz, $CDCl_3$)

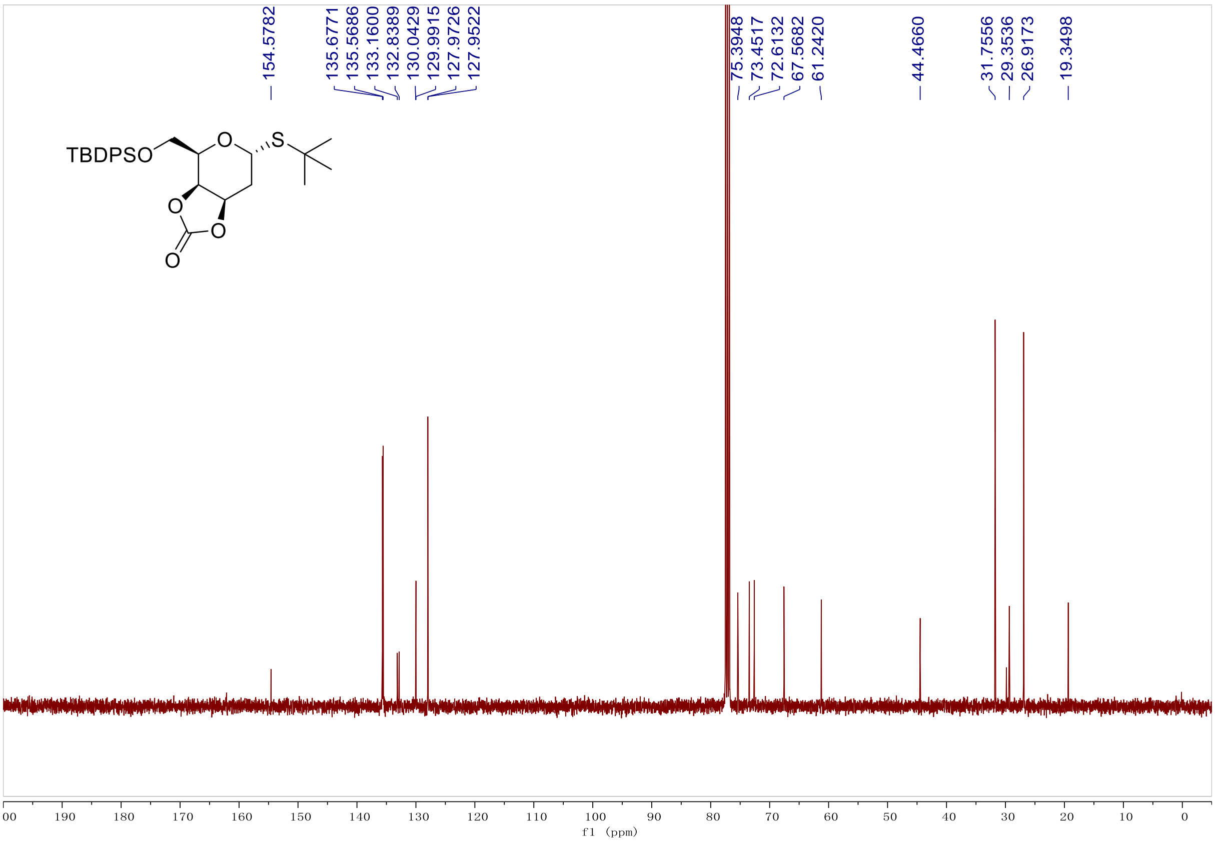

^{1}H NMR of 5g (400 MHz, $CDCl_3$)

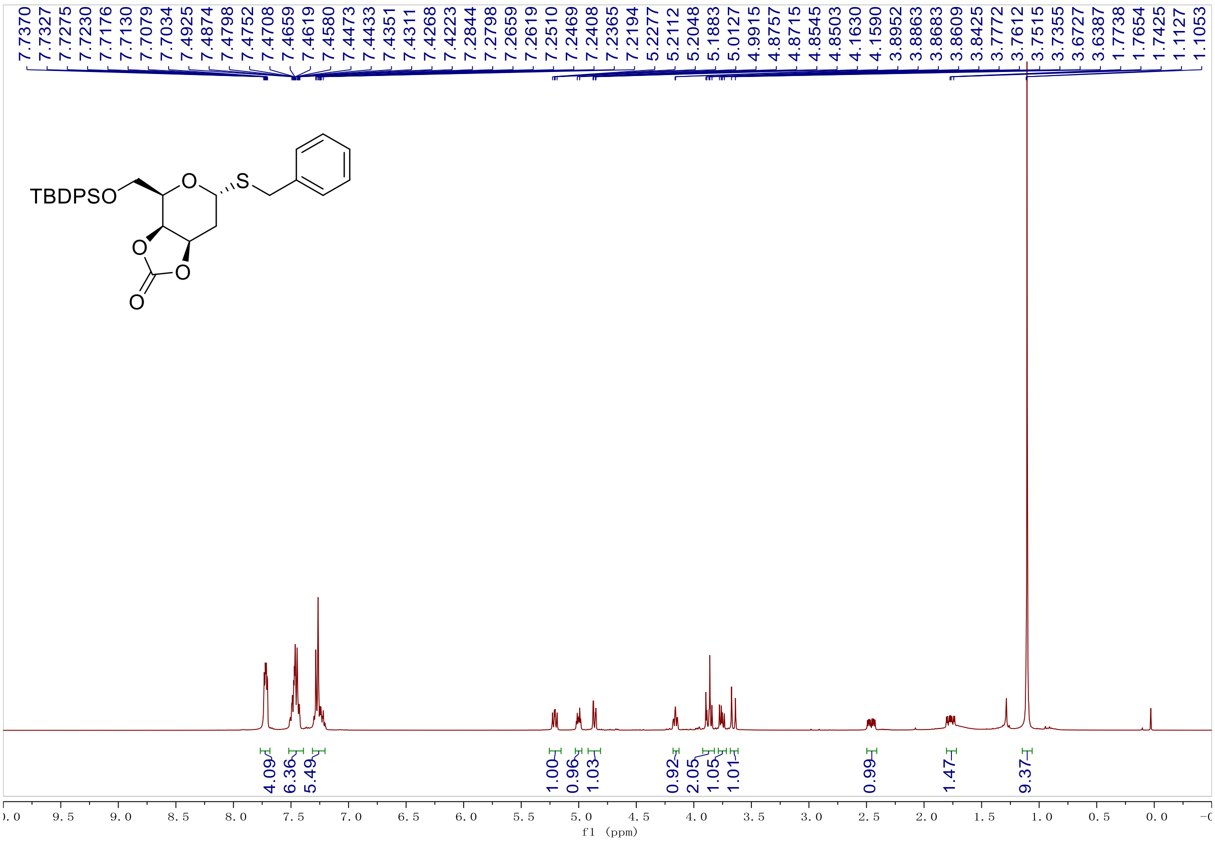

^{13}C NMR of 5g (100 MHz, $CDCl_3$)

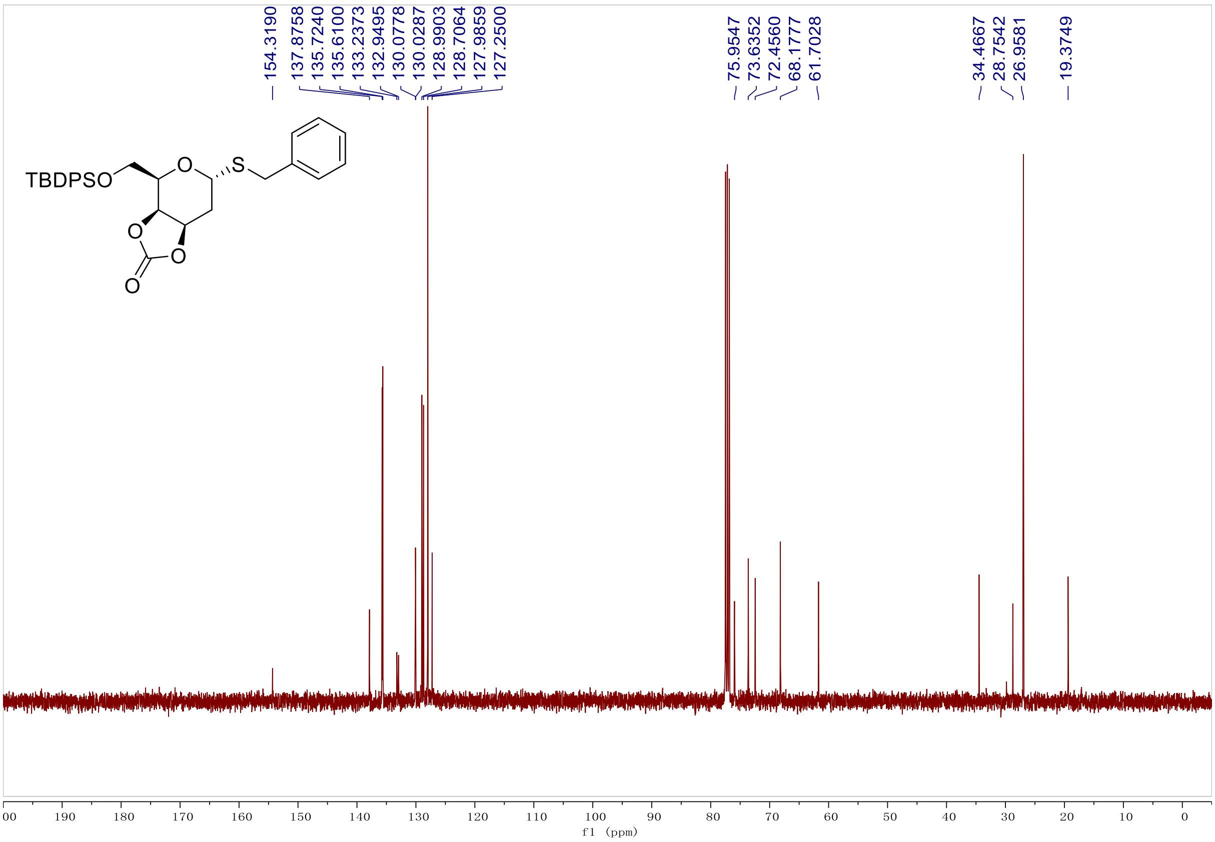

^{1}H NMR of 5h (400 MHz, $CDCl_3$)

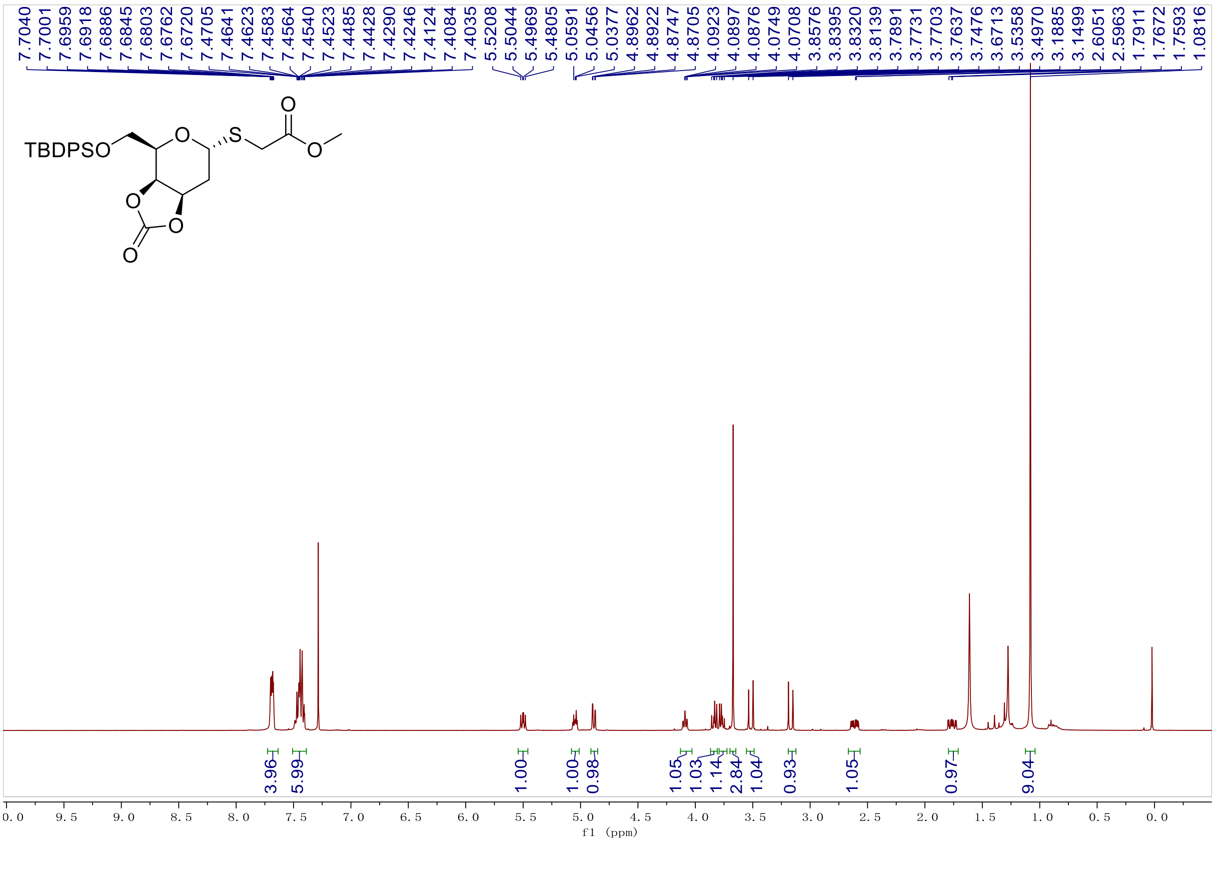

^{13}C NMR of 5h (100 MHz, $CDCl_3$)

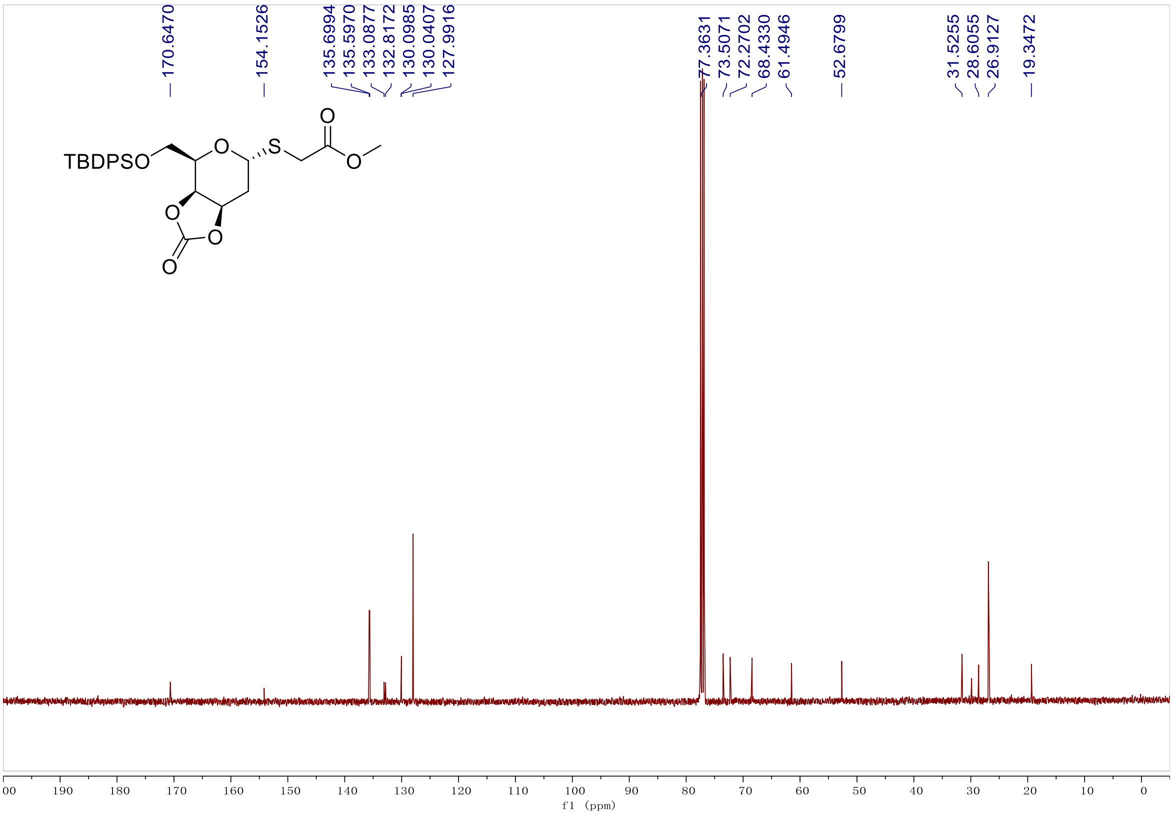

^{1}H NMR of 5i (400 MHz, $CDCl_3$)

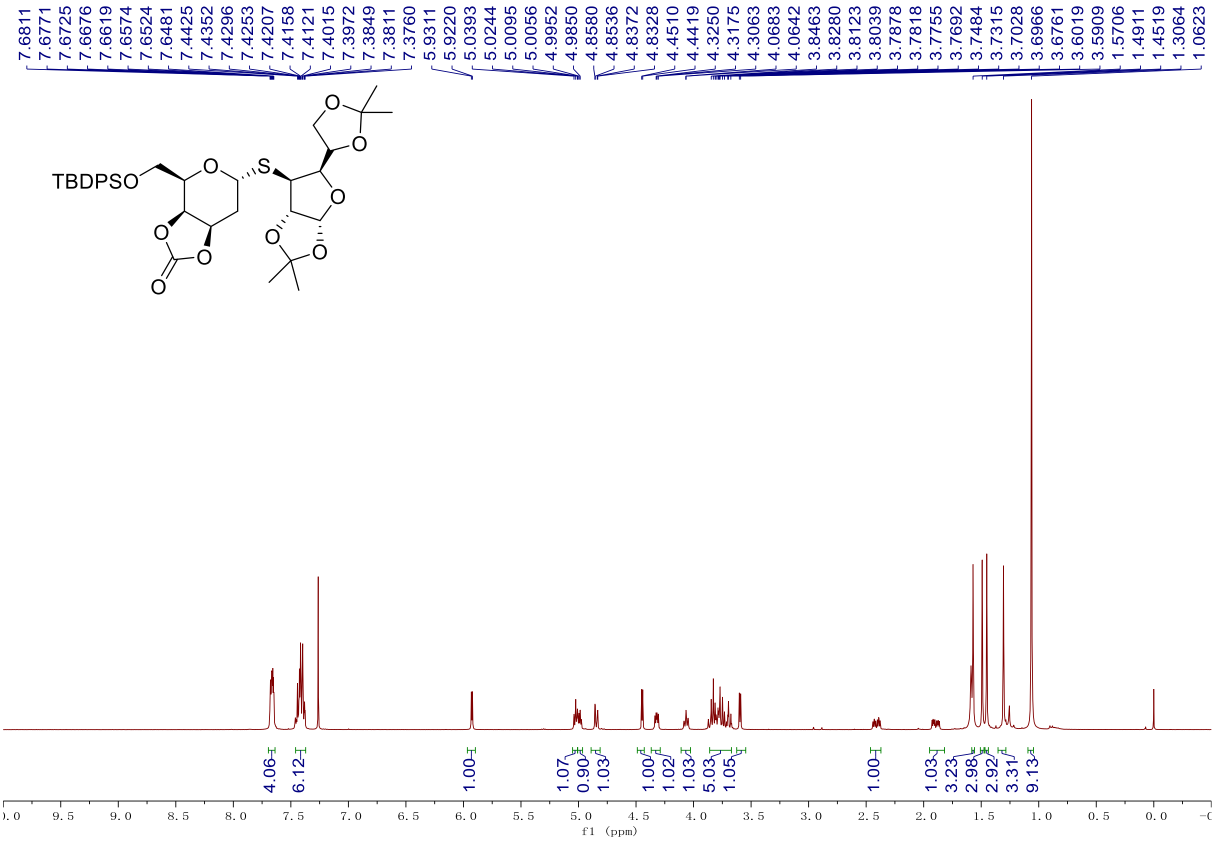

^{13}C NMR of 5i (100 MHz, $CDCl_3$)

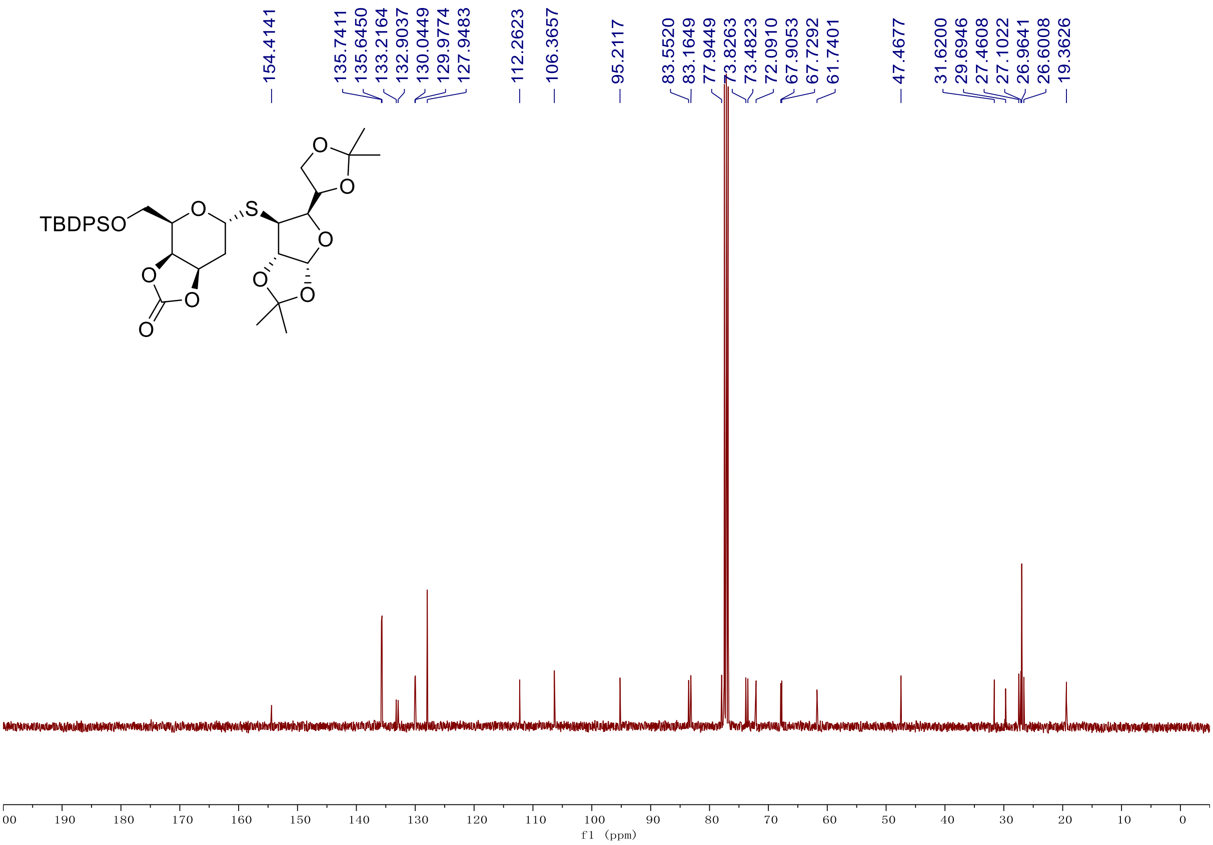

^{1}H NMR of 5j (400 MHz, $CDCl_3$)

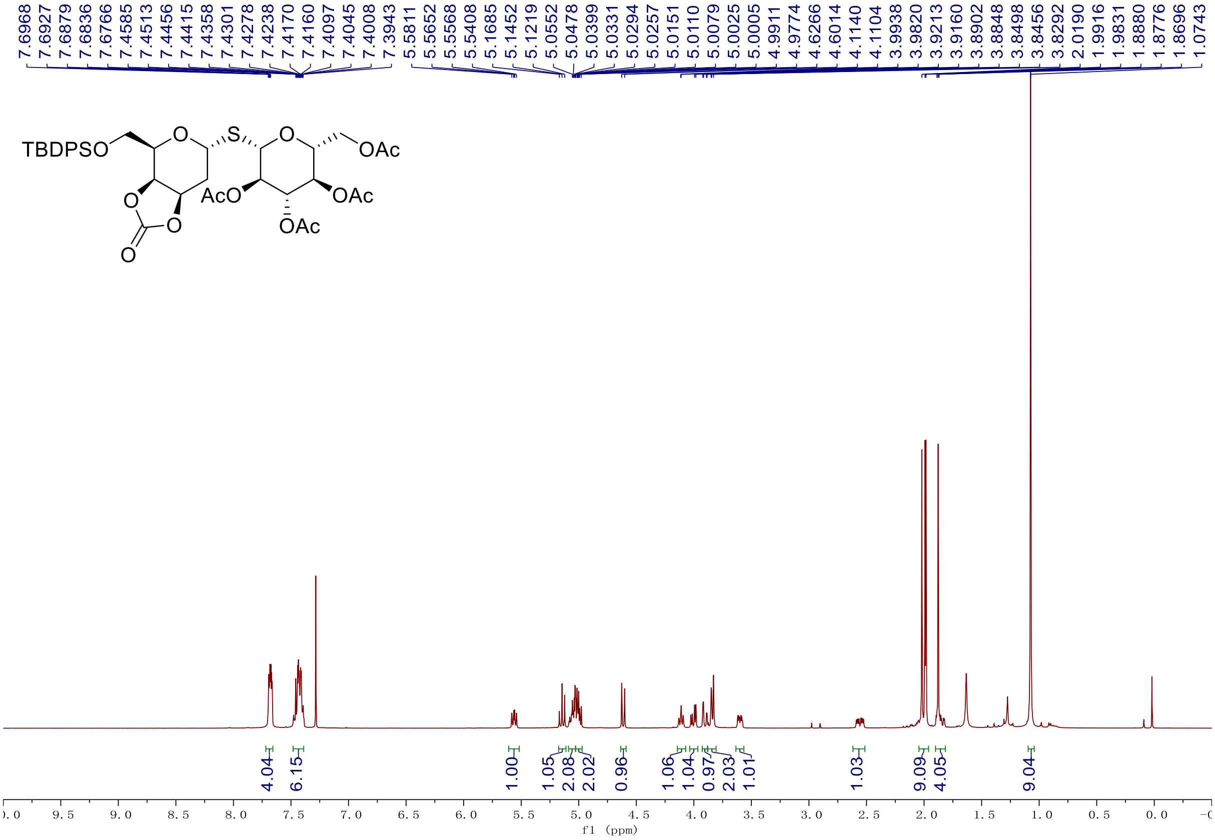

^{13}C NMR of 5j (100 MHz, $CDCl_3$)

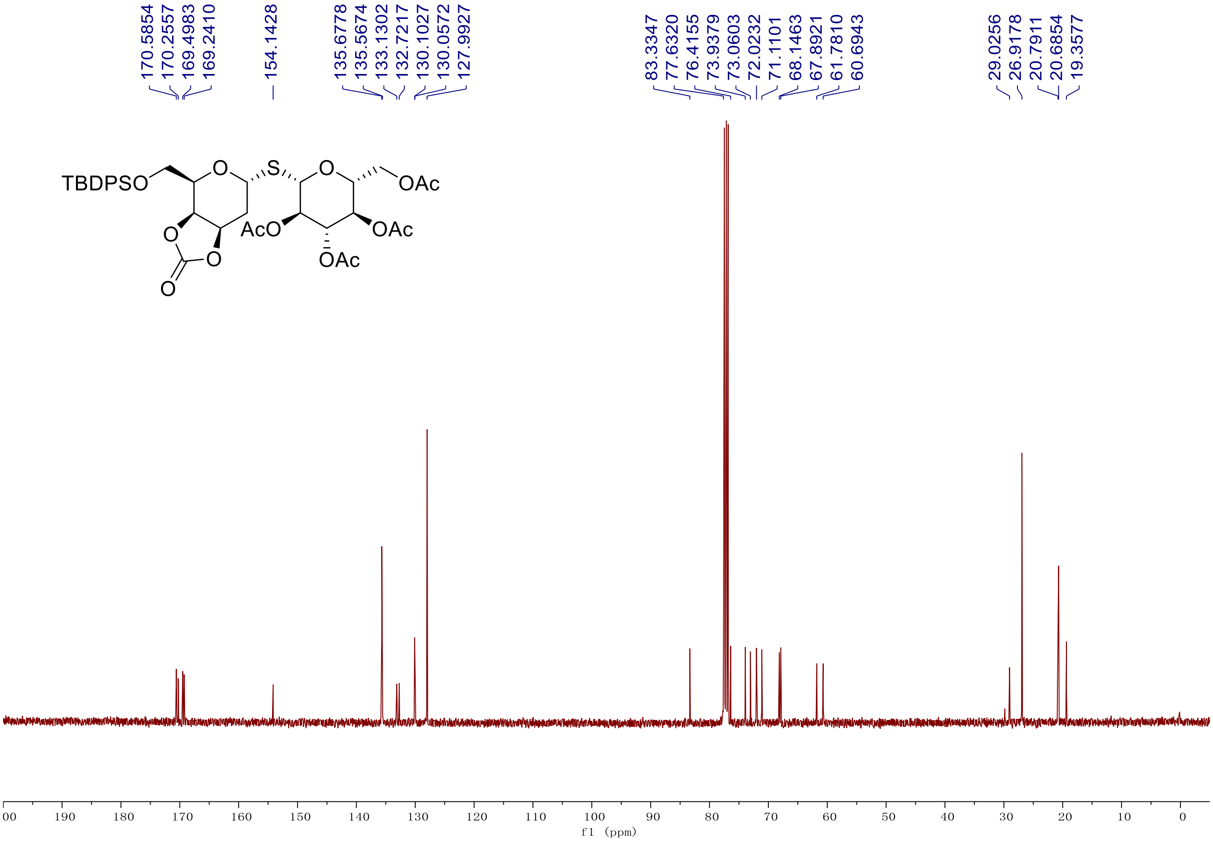

^1H NMR of 7a (400 MHz, $CDCl_3$)

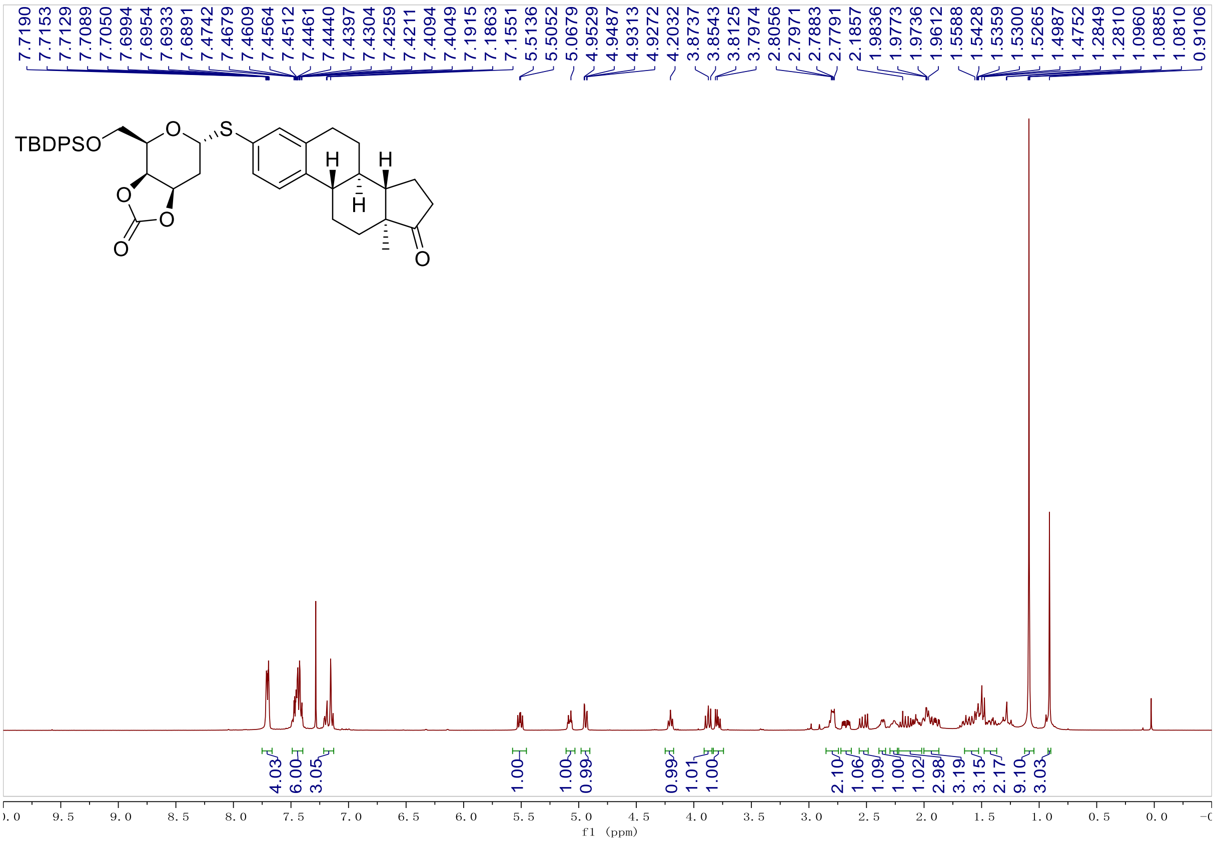

^{13}C NMR of 7a (100 MHz, $CDCl_3$)

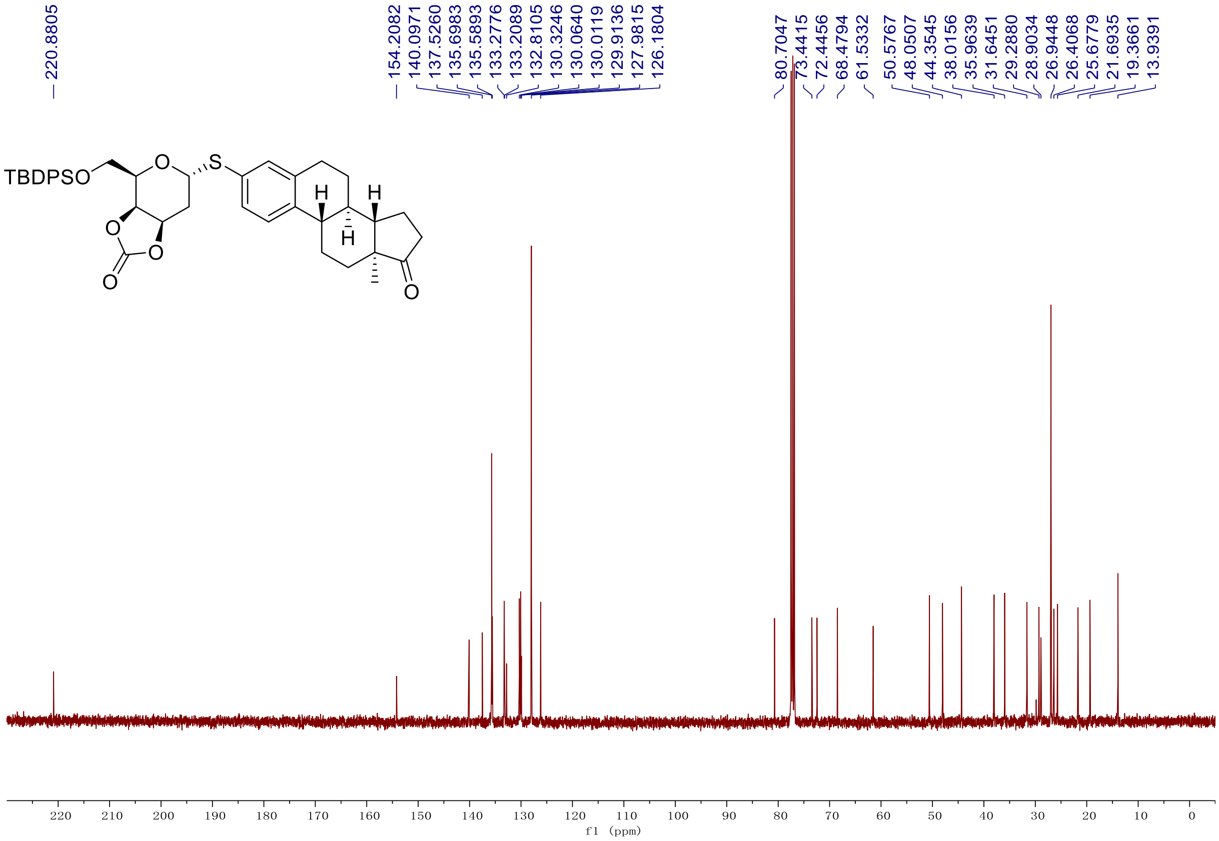

^{1}H NMR of 7b (400 MHz, $CDCl_3$)

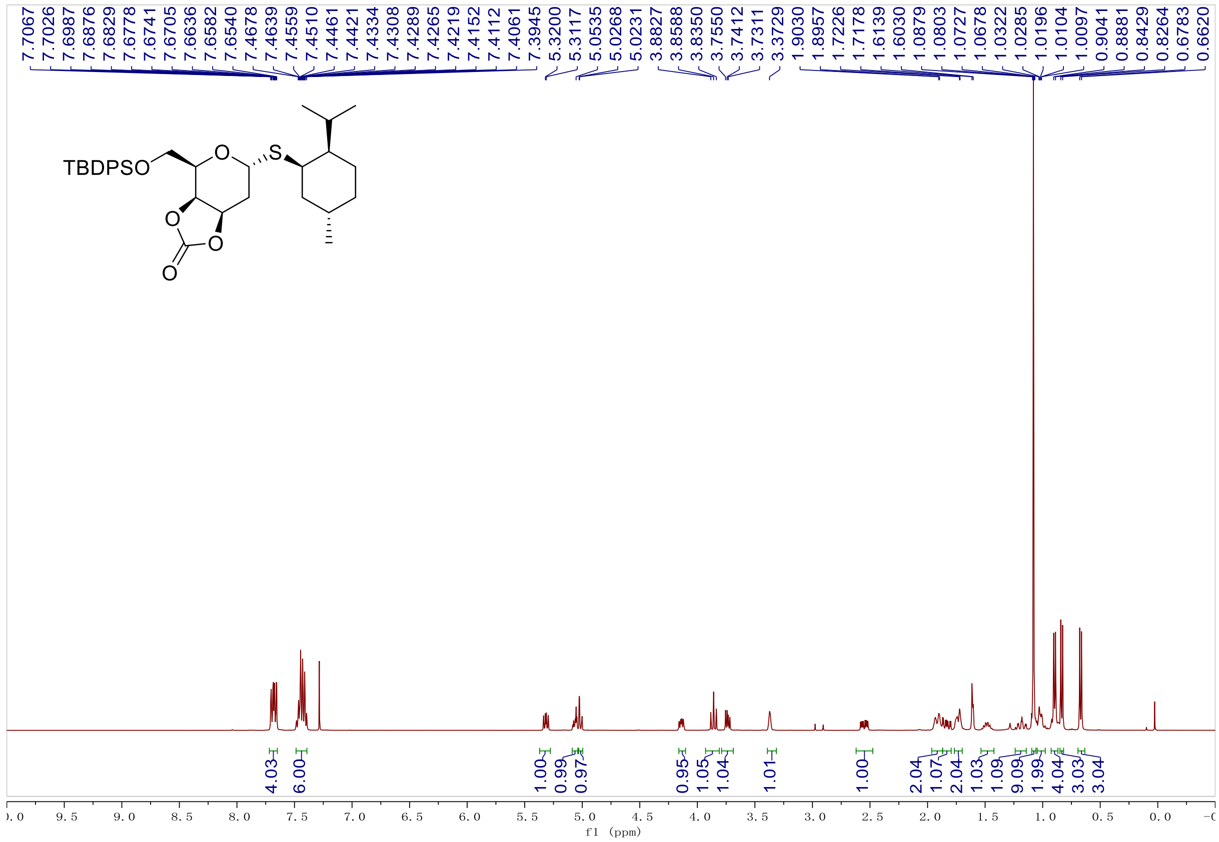

^{13}C NMR of 7b (100 MHz, $CDCl_3$)

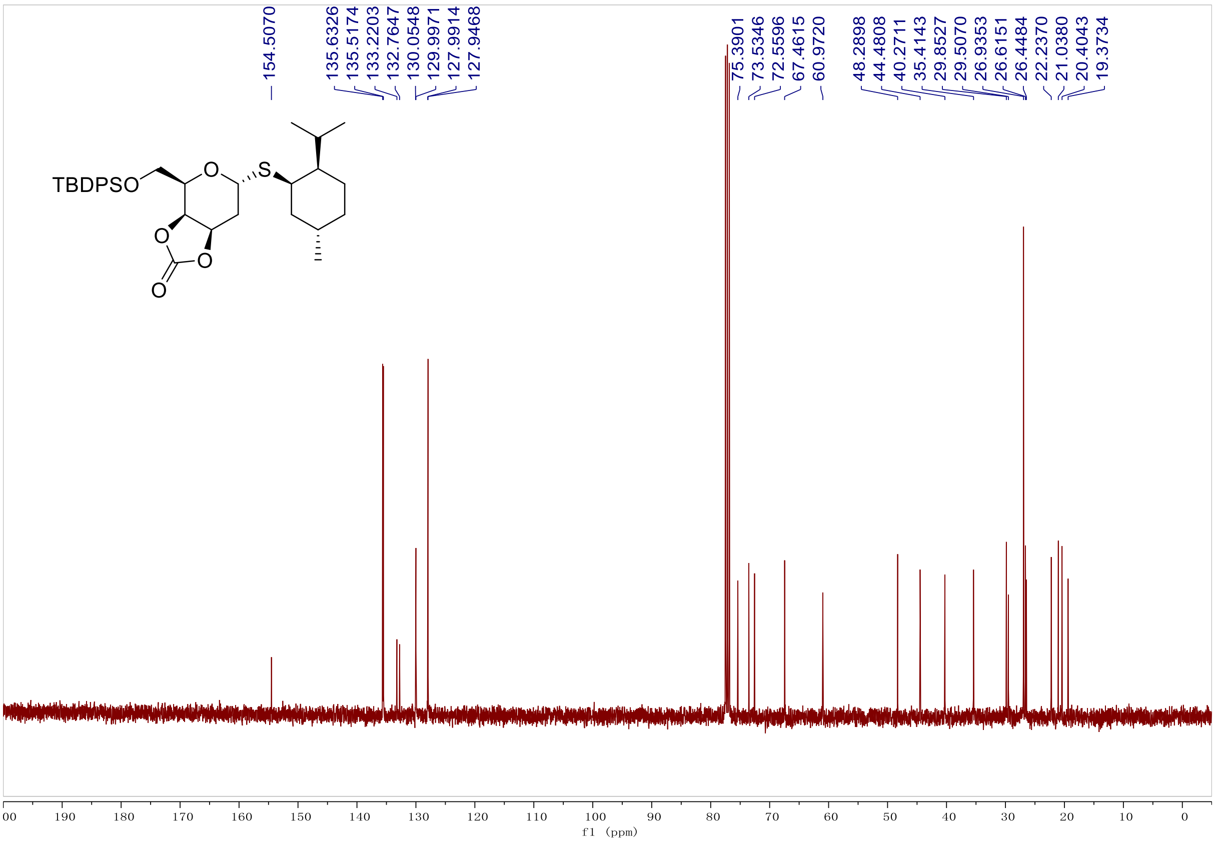

^1H NMR of 7c (400 MHz, $CDCl_3$)

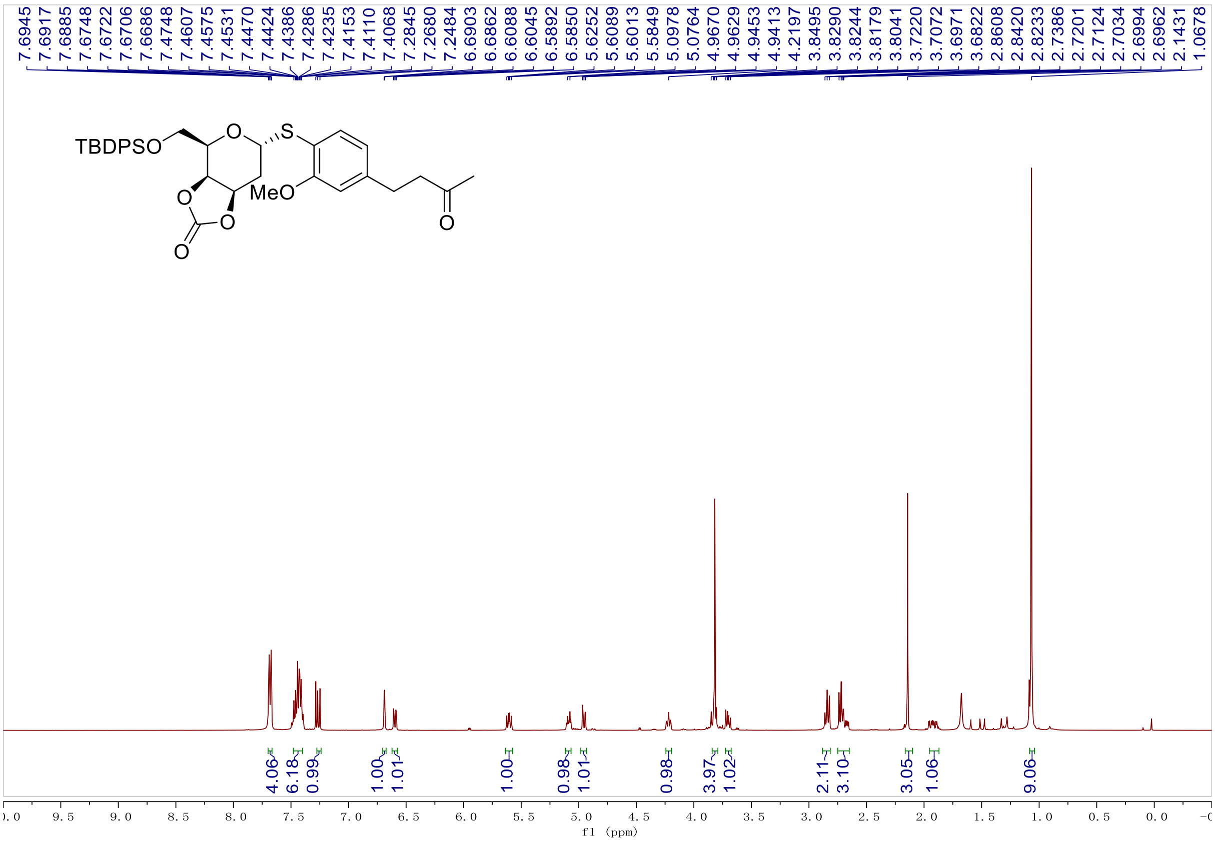

^{13}C NMR of 7c (100 MHz, $CDCl_3$)

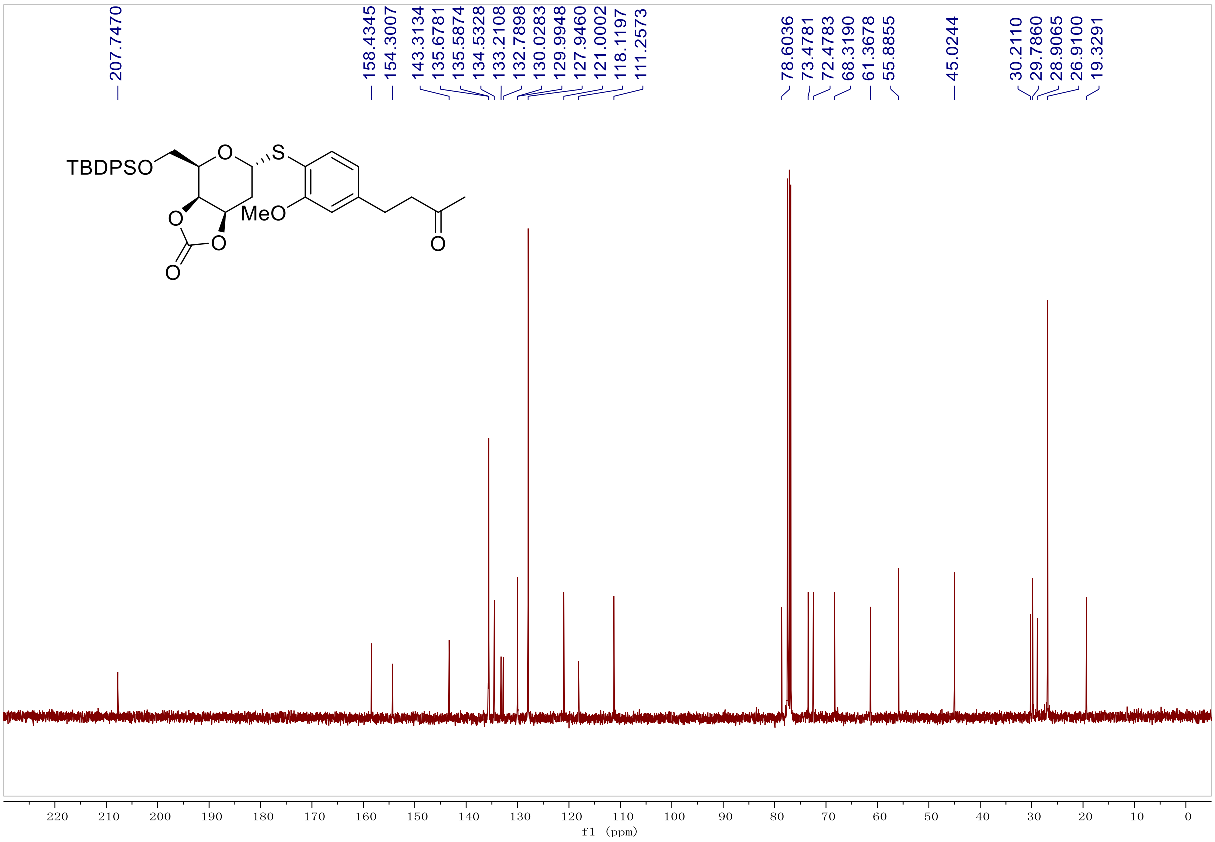

Section D: X-ray Crystal Structure and Data of **3c**

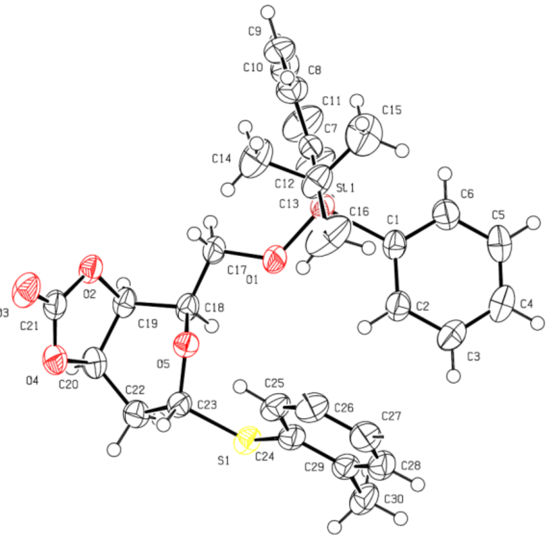

Figure S4. ORTEP drawing of compound **3c** showing thermal ellipsoids at the 50% probability level (CCDC: 2212881).

Table S1. Crystal data and structure refinement for **3c**.

Compound	**3c**
Empirical formula	$C_{30}H_{34}O_5SSi$
Formula weight	534.72
Temperature/K	149.99(10)
Crystal system	monoclinic
Space group	$P2_1$
a/Å	10.5384(4)
b/Å	9.4637(3)
c/Å	14.2365(5)
α/°	90
β/°	100.057(3)
γ/°	90
Volume/$Å^3$	1398.02(9)
Z	2
$\rho_{calc}g/cm^3$	1.270
μ/mm^{-1}	1.742
F(000)	568.0
Crystal size/mm^3	0.12 × 0.1 × 0.08
Radiation	Cu Kα (λ = 1.54184)
2Θ range for data collection/°	6.306 to 148.906
Index ranges	$-12 \le h \le 13$, $-11 \le k \le 11$, $-17 \le l \le 17$
Reflections collected	18594
Independent reflections	5309 [R_{int} = 0.0757, R_{sigma} = 0.0529]
Data/restraints/parameters	5309/1/338
Goodness-of-fit on F^2	1.039
Final R indexes [I>=2σ (I)]	R_1 = 0.0524, wR_2 = 0.1356
Final R indexes [all data]	R_1 = 0.0580, wR_2 = 0.1441
Largest diff. peak/hole / e $Å^{-3}$	0.43/-0.34
Flack/Hooft parameter	-0.021(18)/-0.028(13)
